# Parental Risk and Protective Factors Associated with Bullying Victimization in Children and Adolescents: A Systematic Review and Meta-analysis

**DOI:** 10.1007/s10567-024-00473-8

**Published:** 2024-05-08

**Authors:** Diana Ioana Grama, Raluca Diana Georgescu, Iulia Maria Coşa, Anca Dobrean

**Affiliations:** 1 Doctoral School “Evidence-Based Assessment and Psychological Interventions”, Babeş-Bolyai University, Cluj-Napoca-Napoca, Romania; 2 The International Institute for the Advanced Studies of Psychotherapy and Applied Mental Health, Babeş-Bolyai University, Cluj-Napoca-Napoca, Romania; 3 Department of Clinical Psychology and Psychotherapy, Babeş-Bolyai University, Republicii Street 37, 400015 Cluj-Napoca-Napoca, Romania

**Keywords:** Bullying victimization, Child, Adolescent, Parental factors, Meta-analysis

## Abstract

The main objective of this meta-analysis was to investigate how modifiable parental factors are related to traditional and cyberbullying victimization in children and adolescents. A systematic literature search of modifiable parental factors associated with bullying victimization was conducted using PubMed, PsycINFO, Scopus, and Web of Science electronic databases. Meta-analyses were performed to assess the mean effect sizes of the associations between the broader categories of parental factors (risk and protective) and bullying victimization (traditional and cyber), as well as between specific parental factors and bullying victimization (traditional and cyber). The differential impact of maternal and paternal factors (risk and protective) was examined. Age and gender were tested as moderators. Out of the 13,171 records identified, 158 studies met the inclusion criteria. Larger evidence was found for the association between parental risk (i.e., authoritarian parenting, aversiveness, inter-parental conflict, over-involvement, permissive parenting, and withdrawal) and protective (i.e., authoritative parenting, autonomy granting, warmth, and monitoring) factors, respectively, and traditional bullying victimization, with parental warmth, aversiveness, and withdrawal being the only common related predictors for traditional and cyberbullying victimization. The effect sizes were generally small. Maternal and paternal factors showed similar patterns of association with both types of bullying victimization. Age had a moderating effect on the association between parental protective factors and cyberbullying victimization. Overall, the present findings suggest that parental factors are relevant in protecting or putting children at risk for bullying victimization, especially in the offline context.

**Supplementary Information:**

The online version contains supplementary material available at 10.1007/s10567-024-00473-8.

## Introduction

Bullying is recognized as a global critical health problem, with an average of 30.5% of children being bullied over the course of a month (Biswas et al., 2020). Recent estimates on the prevalence of both traditional and cyberbullying victimization have indicated a similar mean occurrence, with 28.9% of children being bullied, 17.7% reporting being a victim of traditional bullying, 5.1% reporting being a victim of cyberbullying, and 6.1% reporting being a victim of both types of bullying (Chudal et al., 2022). In its traditional forms, bullying victimization takes place mainly in schools, while cyberbullying victimization occurs through the use of technology. Regardless of the context in which bullying victimization takes place, bullied children are prone to a range of adverse psychosocial and academic outcomes, such as low self-esteem, anxiety, depression, suicidal thoughts and behaviors, poorer school performance, and absenteeism (Halliday et al., 2021; Holt et al., 2015; Kowalski et al., 2014; Moore et al., 2017). Moreover, the negative impact of bullying victimization tends to persist long after the bullying has stopped (Arseneault, 2018; Copeland et al., 2013; Pabian & Vandebosch, 2021; Ttofi et al., 2011; Wolke & Lereya, 2015), resulting in subsequent individual, familial, and societal costs (Brimblecombe et al., 2018; Evans-Lacko et al., 2017; Jadambaa et al., 2021; Takizawa et al., 2014).

Given the high prevalence and the burden of outcomes associated with bullying victimization, consistent efforts have been made to identify risk and protective factors that could serve as targets in anti-bullying programs. From a socio-ecological perspective (Bronfenbrenner, 1979), bullying victimization occurs as a function of complex interactions between individual and contextual factors, like family, peer, school, and community characteristics (Swearer & Hymel, 2015). The family is considered the first socialization context in which children acquire interpersonal skills and abilities, which they later transfer to their peer context (Duncan, 2004; Ladd, 1992), thus making families a good target for anti-bullying prevention and early intervention programs. Considering this perspective, Gaffney et al., (2019a, 2019b, 2021) have conducted several meta-analyses to quantify the effectiveness of anti-bullying programs and explore mechanisms of change. Results have shown that anti-bullying programs are effective in reducing traditional (Gaffney et al., 2019a) and cyberbullying victimization (Gaffney et al., 2019b) prevalence by approximately 15%. Furthermore, findings have indicated that among the most important components of school-based anti-bullying programs linked to a significant reduction in bullying victimization is parental involvement (e.g., providing parents with information about bullying or the intervention through letters or leaflets) (Gaffney et al., 2021). In line with these results, a meta-analysis (Huang et al., 2019) that has assessed the effectiveness of school-based anti-bullying programs with parental components indicates a small but significant overall effect (d = 0.162 (95% CI = [0.059, 0.265], p = .004). Similarly, the effectiveness of parental components in reducing cyberbullying victimization has been noted by Hutson et al. (2018) in a qualitative analysis, showing that interventions with parent education components are among the successful programs in reducing cyberbullying victimization.

Anti-bullying interventions with parental components usually inform parents about school-implemented interventions, raise parents’ awareness and understanding of bullying, increase parent-school communication, or enhance parental involvement and monitoring through tasks at home (e.g., Cross et al., 2012; Joronen et al., 2012). However, most anti-bullying programs tend not to directly target specific parental factors (Axford et al., 2015), even though various aspects of parenting can increase or decrease the risk of becoming a victim of bullying. Longitudinal studies have shown that parental rejection (Stavrinides et al., 2018), family conflict (Hemphill & Heerde, 2014), psychological control (Wu et al., 2022), harsh parenting (Whelan et al., 2014), authoritarian parenting, and permissive parenting (Charalampous et al., 2018) are all risk factors for being bullied. Additionally, factors such as family support (Fanti et al., 2012), parental supervision (Le et al., 2017), or authoritative parenting (Charalampous et al., 2018) predict lower levels of bullying victimization. The existing conceptual models suggest these parental factors are risk or protective to the extent that they influence children’s characteristics that create proximal vulnerability to bullying victimization (e.g., Shin et al., 2016; Kaufman et al., 2020; Chen et al., 2022; Samper-García et al., 2021; Li et al., 2015; Charalampous et al., 2018). Specifically, negative parenting has been shown to predict more socio-emotional and behavioral difficulties in children, which, in turn, increase their risk of being bullied. Conversely, positive parenting has been shown to predict positive adjustments in these areas, which subsequently protect children against bullying victimization.

Prior systematic reviews and meta-analyses have explored several parental predictors of bullying victimization, but they have assessed wider parental or family concepts, such as “family/home environment” (Cook et al., 2010) or “negative family environment” (Guo, 2016), while others have conducted qualitative analysis (Elsaesser et al., 2017; Nocentini et al., 2019). Moreover, while several meta-analyses have focused on parental predictors of cyberbullying victimization, they have neglected the role of parents in traditional bullying victimization (e.g., Chen et al., 2017; Guo, 2016; Kowalski et al., 2014). Overall, researchers have found small but significant effect sizes, regardless of the parental component that was considered. For instance, Cook et al. (2010) have found a small negative association between positive home environment and school bullying victimization, while Guo (2016) has reported a small positive association between negative family environment and cyberbullying victimization. Chen et al. (2017) have found small negative associations between parental interaction and parental mediation, respectively, and cyberbullying victimization. Conversely, Kowalski et al. (2014) have found a small negative association between parental monitoring and cyberbullying victimization, but a non-significant association between parental control of technology and cyberbullying victimization.

Only one systematic review has performed a quantitative synthesis specifically on the role of multiple parental factors in bullying victimization (Lereya et al., 2013). Overall, findings have indicated that victims of bullying are more likely to be exposed to abuse, neglect, overprotection, and maladaptive parenting. Conversely, authoritative parenting, good communication with parents, warm and affectionate relationships, parental involvement and support, and parental supervision have been shown to protect against bullying victimization. The effect sizes were significant and generally small to moderate. This meta-analysis has reported on the association between parental factors and traditional and cyberbullying victimization combined. To our knowledge, there is currently no systematic review or meta-analysis on the differential associations with bullying victimization occurring in the school context versus using technology. While some studies have reported similarities in how parent–child relationships influence traditional and cyberbullying victimization (e.g., Katzer et al., 2009), other studies have highlighted several differences (e.g., Hemphill & Heerde, 2014). Similarly, no synthesis has explored whether maternal and paternal factors are distinctly associated with traditional and cyberbullying victimization. This is not surprising since studies have focused mainly on the mother–child relationship while neglecting the role of the father. However, a growing interest in maternal and paternal contributions to a child’s development allows us to now approach bullying victimization from this perspective as well.

Even though previous reviews and meta-analyses have highlighted the role of various parental factors in bullying victimization, further clarification is needed. In the past decade, a wide range of research has emerged on the role of parental factors in bullying victimization, allowing us to explore from different perspectives the modifiable parental factors that might impact on bullying victimization as well as to obtain a more comprehensive picture by synthesizing the results through a meta-analysis. Assessing whether parental factors are concurrently associated with both traditional and cyberbullying victimization and if there is a differential impact of maternal and paternal factors on both types of bullying victimization could extend the approach of future prevention and anti-bullying intervention programs.

Therefore, the present meta-analysis aimed to investigate the role of parental factors in traditional as well as cyberbullying victimization among children and adolescents. The first main objective was to determine which parental factors are protective and which are those that put children at risk of being bullied in the school context and using technology as well as to assess the magnitude of the associations. The second goal was to evaluate whether maternal and paternal factors (i.e., risk and protective) are differently associated with bullying victimization (i.e., traditional and cyber). The third goal was to assess potential moderators (i.e., age and gender) of the association between parental factors (i.e., risk and protective) and bullying victimization (i.e., traditional and cyber).

## Methods

### Protocol and Registration

This systematic review and meta-analysis was conducted according to the Preferred Reporting Items for Systematic Reviews and Meta-Analyses (PRISMA) (Moher et al., 2010) and the Cochrane Handbook (Higgins & Green, 2011). The study protocol was registered on the International Prospective Register of Systematic Reviews (PROSPERO reference number CRD42021240629).

### Searching Strategy

To identify relevant articles on the relationship between parental factors and bullying victimization, a literature search was conducted on March 12, 2021, and updated on November 1, 2023, in the PubMed, PsycInfo, Scopus, and Web of Science electronic databases, using the following search string: (((((parent* OR family* OR caregiv* OR mother* OR father* OR maternal OR paternal) AND (child* *bully* *victim*) OR (child* *bulli* *victim*) OR (adolescent* *bully* *victim*) OR (adolescent* *bulli* *victim*))))). Searches were conducted without limitations on language, country, or publication date. Furthermore, the bibliographies of the included articles in this review, as well as the references cited in prior systematic reviews and meta-analyses, were scrutinized to identify any additional pertinent studies.

### Inclusion and Exclusion Criteria

Studies were eligible for inclusion if they: (1) examined the relationship between at least one parental factor and bullying victimization; (2) assessed the parental factors with a validated instrument; (3) reported quantitative data necessary to calculate effect sizes; (4) were cross-sectional, case–control, longitudinal, or intervention studies (studies involving interventions and longitudinal design were eligible only if baseline/first wave data were available); (5) had a sample consisting of children and adolescents ≤ 18 years old; (6) were peer-reviewed; (7) were written in English, German, or French. Studies were excluded if they: (1) assessed forms of victimization other than bullying victimization; (2) measured sibling bullying victimization; (3) measured bullying victimization outside the school context; and (4) measured traditional and cyberbullying victimization combined. In addition, as they are more prone to biases due to a less rigorous review process, we excluded dissertations, letters to the editor, and conference abstracts.

### Study Selection

After completing the electronic search, duplicates were removed, and all the titles and abstracts were screened by two independent reviewers using EndNote. Irrelevant articles were excluded. The remaining articles were full-text screened by the two researchers according to the inclusion and exclusion criteria. All discrepancies were discussed with a third researcher and resolved by consensus.

### Data Extraction

Two researchers independently used a standardized spreadsheet to extract the data from all eligible articles. Any disagreements between the coders were reviewed and corrected using the source text of the respective primary study, and unclear situations were resolved by consulting a senior researcher. The following data were extracted from each included study: the identification data (authors, publication year); the data necessary to calculate effect sizes (i.e., r and sample size); the sample characteristics (i.e., mean age/age range/grades, gender, sample size, country); the type of parenting variable, the specific scales used to measure the parental factors, as well as the informant (i.e., self or others); the type of bullying victimization (i.e., traditional or cyber), the specific scales used to measure bullying victimization, the informant (i.e., self or others), as well as the reference time frame for bullying victimization.

### Coding

Given the heterogeneity of parenting constructs, Yap et al.’s (2014) conceptual model of parenting was used as a framework for our data. It is based on two broad dimensions: rejection and control (Maccoby, 1994), each including several subdimensions that have been outlined before by McLeod et al. (2007): rejection comprises withdrawal, aversiveness, and warmth; control includes over-involvement and autonomy-granting. Yap et al. (2014) formulated four more categories for variables that did not fit the ones stated above: inter-parental conflict, monitoring, encouraging sociability, and discipline. Discipline was further divided into permissive parenting, authoritarian parenting, authoritative parenting, and inconsistent discipline (for definitions see Appendix A). Thus, parental factors linked to bullying victimization were coded and included in the meta-analysis according to the model described above. Each parental factor was coded by two independent reviewers. Discrepancies were resolved through discussion, and further disagreements were discussed with a third researcher.

### Meta-analytical Procedure

We used the software packages Comprehensive Meta-Analysis (CMA v. 2.2.064) for computing study-level effect estimates and Stata SE 16.0 (STATA Corp., Inc., College Station, TX) packages Meta (Wilson, 2022) for pooling, Metabias (Harbord et al., 2009) for testing small study effects, Hetergi (Orsini et al., 2006) for computing the 95% CIs of *I*^*2*^, and Confunnel (Palmer et al., 2008) for visualization. The Pearson correlation coefficient (r) was employed in combination with the sample size (N) of each study to determine the pooled effect size (ES). To enable comparability and facilitate subsequent analyses, the overall effect sizes (r) were transformed into Fisher's z scores. For presentation, the z scores were converted back to correlation coefficients. When *r* correlation coefficients and sample sizes were not available, we asked authors to provide the data, and in cases of no response, the effect sizes were estimated based on other available data (i.e., t-value and sample size; unadjusted odd ratio and confidence interval). The magnitude of the associations was interpreted based on the guidelines provided by Cohen (1988), with values of .10, .30, and .50 indicating small, medium, and large effect sizes, respectively. Meta-analyses were conducted to estimate the magnitude of the association between each parental factor and bullying victimization (i.e., traditional and cyber), as well as between the broader categories of parental factors (i.e., risk and protective) and traditional and cyberbullying victimization, respectively. Given the diversity of parental variables, we expected a high degree of between-study heterogeneity. Thus, a random effects model was conducted. The heterogeneity of the effect sizes was estimated using the *I*^*2*^ index, which reflects the percentage of variation across studies that is due to heterogeneity rather than chance (Higgins & Thompson, 2002). I^2^ values around 25%, 50%, and 75% indicate low, moderate, and high heterogeneity, respectively (Higgins, 2003). A series of sensitivity analyses were also performed. Firstly, we excluded outliers by identifying those studies whose confidence interval did not overlap with the confidence interval of the pooled effect. We also computed the effect size for fair and good-quality studies that reported on the association between parental risk and protective factors and traditional and cyberbullying victimization, respectively. Finally, separate effect sizes were calculated for the relationship between maternal and paternal factors (i.e., risk and protective) and bullying victimization (i.e., traditional and cyber). For assessing the impact of continuous moderators, we used meta-regression analysis and a restricted maximum likelihood model. We tested whether there was a significant relationship between the mean age and the percentage of girls, respectively, and the main effect sizes (i.e., parental risk and protective factors associated with traditional and cyberbullying victimization). When the mean age was not available, we computed it based on the reported age range or the grades the students were in, considering the country in which students were studying.

### Quality Assessment

Quality assessments for the eligible studies were carried out using the *NIH Quality Assessment Tool for Observational Cohort and Cross-Sectional Studies* (NIH, 2014). It consists of 14 items that address the major components of the articles, such as the research question (e.g., “Was the research question or the objective of this paper clearly stated?”) or the study population (e.g., “Was the study population clearly specified and defined?”). Items were answered with "yes”, “no”, or “cannot determine/not applicable”. A quality score was provided for each study based on the items rated with an affirmative answer: ≥ 75% = good, 50–75% = fair, ≤ 50% = poor. The overall quality of each included study was assessed by two independent reviewers. The degree of agreement between the two reviewers was evaluated by computing Kappa (Munoz & Bangdiwala, 1997). All disagreements concerning the methodological quality of the articles were discussed and resolved by consensus.

### Publication Bias

A recurring issue in meta-analyses is that research with non-significant findings may remain unpublished, whereas studies with significant findings have a better chance of being published (Song et al., 2010). As a result, the sample of included studies in our meta-analyses could be incomplete and not representative of the population of research, causing us to overestimate or underestimate the effects of parental factors on bullying victimization. As such, first, we created funnel plots for the broader categories of parental factors (i.e., risk and protective) and each type of bullying victimization (i.e., traditional and cyber), in which the effect sizes were plotted against their standard errors, and we visually inspected whether data points were spread symmetrically within the funnel. In addition, we constructed contour-enhanced funnel plots with contour lines indicating regions where the association was significant at 90, 95, and 99% statistical significance levels (Peters et al., 2008). Second, Egger’s test was used to examine whether there is a tendency toward selectivity in publishing studies based on their nature and direction of results. In the linear regression analysis, the intercept value is an indicator of asymmetry; the larger its deviation from zero, the higher the degree of asymmetry (Egger et al., 1997).

## Results

### Selection and Inclusion of Studies (Fig. 1)

**Fig. 1:**
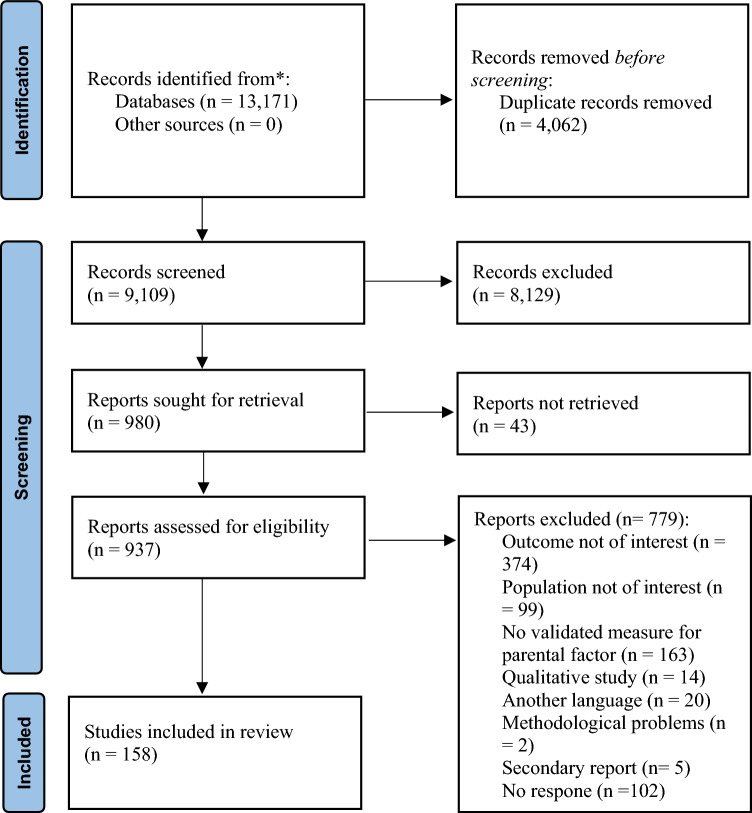
PRISMA diagram

A total of 13,171 records were identified through databases. 4062 duplicates were removed, and the remaining 9109 articles were screened by title and abstract. 8129 records were further excluded, yielding a total of 980 studies that were full-text assessed for eligibility. 260 studies met the inclusion criteria, and 145 studies had enough data to calculate the effect size. For studies with missing data, authors were contacted. 13 authors provided the data necessary to calculate the effect size. Finally, 158 studies were included in the systematic review and meta-analysis (see Fig. 1).

### Characteristics of Included Studies (Table 1)

**Table 1:** Selected characteristics of included studies (N = 158)

References	N	Mean age/age range/grade	Percentage of females	BV type^1^	TBV/CBV scale^2^	TBV/CBV time^3^	BV reported by^4^	Parental factor	PF scale^5^	PF reported by^6^	Country	Study quality
Affrunti et al. (2014)	124	8.7/7–12/NR	43.50	Traditional	PRQ	NR	Self	Warmth	PDI-SF	Parent	USA	POOR
Akkurt Nurtan et al. (2022)	550	13.42/NR/9	59.1	Cyber	CVS (a)	NR	Self	Autonomy granting	PAS	Child	Turkey	POOR
								Warmth	PAS			
								Over-involvement	PAS			
Alcantara et al. (2017)	910	11.7/10–16/6–7	52.10	Traditional	EVAP	6	Self	Warmth	SSA	Child	Brazil	FAIR
Alikasifoglu et al. (2007)	3519	NR/14–16/9–11	49.50	Traditional	HBSC	< 12	Self	Warmth	HBSC	Child	Turkey	POOR
Arabiat et al. (2018)	1166	NR/11–16/6–10	52.70	Traditional	HBSC	2	Peers	Warmth	HBSC	Child	Jordan	POOR
Arango et al. (2019)	142	13.6/12–15/NR	74.60	Traditional	PEQ	4	Self	Warmth	PFCS	Child	USA	FAIR
Balan et al. (2018)	476	14 /10–17/NR	58.19	Traditional	APRI-BT	12	Self	Warmth	IPPA-R	Child	Romania	POOR
Balan et al. (2022)	269	11.98/11–15/6–7	48.69	Traditional	APRI	NR	Self	Withdrawal	ECR	Child	Romania	POOR
Bartolomé Gutiérrez and Díaz Herráiz (2019)	769	14.13/13–17/2-3^**a**^	46	Traditional	PVS (a)	NR	Self	Warmth	The Kidscreen-52	Child	Spain	FAIR
Beran and Violato (2004)	3434	NR/10–11/NR	44	Traditional	LIKERT SCALE	NR	Self	Over-involvement	PQ	Parent	Canada	POOR
								Warmth	PQ			
Bjereld et al. (2023)	16,057	NR/11–15/5,7,9	NR	Traditional	HBSC	2	Self	Aversiveness	HBSC	Child	Finland & Sweden	POOR
Boniel-Nissim and Sasson (2018)	1000	14.19/12–17/6–11	53	Traditional, cyber	SPRM/CS	< 12/ < 12	Self	Aversiveness	Loeber et. al., (1998) scale	Child	Israel	POOR
								Warmth	Loeber et. al., (1998) scale			
Bordin and Handegård (2023)	669	13.01/11–15/NR	51.7	Traditional	LIKERT SCALE	6	Self	Warmth	EMBU	Child	Brazil	POOR
Carter et al. (2020)	1078	11.24/10–12/NR	59.10	Traditional	APRI-BT	NR	Self	Authoritarian style	CRPBI-30	Child	South Africa	POOR
								Over-involvement	CRPBI-30			
								Warmth	CRPBI-30 + IPPA-R			
Carter et al. (2023)	465	11.27/10–12/5–6	58.9	Traditional	APRI-BT	NR	Self	Warmth	IPPA-R	Child	South Africa	POOR
Cassiani-Miranda et al. (2022)	1462	14.4/13–17/10–11	58.92	Cyber	YRBSQ	NR	Self	Aversiveness	The Family APGAR	Child	Colombia	FAIR
Cassidy (2009)	461	13.1./11–15/NR	57.04	Traditional	NOMINAL SCALE	NR	Self	Autonomy granting	PES	Child	UK	POOR
								Warmth	FES			
Cerezo et al. (2018)	847	12.73/9–12/NR	46.87	Traditional	Bull-S	NR	Peers	Autonomy granting	FES	Child	Spain	POOR
								Aversiveness	ESPA29			
								Warmth	FES			
Chang et. al. (2015)	1808	NR/NR/7–9	51.68	Cyber	EU Kids	NR	Self	Monitoring	EU Kids	Child	Taiwan	POOR
								Warmth	EU Kids			
								Over-involvement	EU Kids			
Charalampous et al., (2018)	761	11.72/10–15/5–8	52	Traditional, cyber	BVQ, PECK	NR/4	Self	Authoritarian style	PAQ	Child	Cyprus	FAIR
								Authoritative style	PAQ			
Charalampous et al. (2019)	406	16.01/15–18/NR	55.40	Traditional	OBVQ-R	NR	Self	Warmth	IPPA	Child	Cyprus	POOR
								Withdrawal	IPPA			
Chen (2020)	1932	NR/NR/7–9	48.90	Cyber	CBVS (a)	< 12	Self	Warmth	CASSA	Child	China	POOR
Chen et al. (2021)	1230	NR/8/3	53.2	Traditional	ISCWeB survey	1	Self	Warmth	ISCWeB survey	Child	Taiwan	FAIR
Chen et al. (2022)	4582	12.95/10–17/NR	50.3	Traditional	OBVQ	NR	Self	Aversiveness	EMBU	Child	China	FAIR
								Warmth	EMBU			
								Over-involvement	EMBU			
Chicoine et al. (2021)	498	11.22/NR/6	47.59	Traditional	OBVQ	12	Self	Warmth	FES	Child	Canada	GOOD
Cho and Lee (2018)	12,642	13.11/10–17/5–10	49	Traditional	OBVQ	2	Self	Warmth	HBSC	Child	USA	FAIR
Cho and Norman (2019)	12,642	12.95/10–17/5–10	49	Traditional	HBSC	< 12	Self	Warmth	HBSC	Child	USA	POOR
Cho et al. (2019)	2560	13.11/10–17/5–10	50.1	Traditional, cyber	HBSC	2/NR	Self	Warmth	HBSC	Child	USA	POOR
								Monitoring	HBSC			
Choi and Park (2018)	3660	NR/NR/7	48.50	Traditional	OBVQ	NR	Self	Warmth	SERI	Child	Korea	POOR
Choi (2023)	20,708	14.7/12–17/NR	68	Traditional	CAHMI	NR	Parent	Warmth	CAHMI	Prent	USA	POOR
Chui et al. (2022)	2139	15.20/10–18/6–12	61.76	Traditional	IBS	1	Self	Aversiveness	FAI	Child	China	POOR
Davidson and Demaray (2007)	355	NR/NR/6–8	53	Traditional	BVS	1	Self	Warmth	CASSA	Child	USA	POOR
DeSmet et al. (2021)	1037	15.27/12–18/7–12	49.8	Traditional, cyber	LIKERT SCALE/LIKERT SCALE	6/6	Self	Over-involvement	PPS	Child	Belgium	POOR
								Autonomy granting	PPS			
Doty et al. (2018)	570	NR/NR/6	48.70	Cyber	LIKERT SCALE	1	Self	Warmth	CRHS	Child	USA	POOR
Dudley et al. (2023)	783	13.98/13–15/8–10	56.96	Traditional	CBVS (b)	1	Self, parent	Warmth	Barnes & Olsen’s (1982) scale + FACES	Child	USA	FAIR
Duggins et al. (2016)	373	13.59/10–17/7–10	54	Traditional	LIKERT SCALE	12	Self	Warmth	CTCYS	Child	USA	POOR
Duong et al. (2009)	211	11.9/NR/6	53.55	Traditional	PNI	NR	Peers	Aversiveness	PSDQ	Parent	China	FAIR
Escario et al. (2023)	35,369	NR /14–18/NR	50.1	Cyber	ESTUDES	NR	Slef	Warmth	ESTUDES	Child	Spain	POOR
Espino et al. (2023)	1276	13.88/11–18/7–10	48.8	Cyber	ECIP-Q	2	Slef	Warmth	MSPSS	Child	Spain	POOR
Elledge et al. (2019)	1595	NR/11–16/7–12	51.20	Traditional	LIKERT SCALE	4	Self	Monitoring	GSHS	Child	Jamaica	POOR
Fanti et al. (2012)	1416	12.89/11–14/NR	50.10	Traditional, cyber	SSBB-R	NR/NR	Self	Warmth	MSPSS	Child	Cyprus	FAIR
Fox et al. (2022)	178	9.58/8–11/4–5	45.5	Traditional	SPVS	2 weeks	Slef	Over-involvement	EMBU	Child	USA	POOR
Fredrick et al. (2022)	801	14.45/13–15/7–11	57	Traditional	CBVS (b)	6	Self	Warmth	FACES	Child	USA	GOOD
Freitas et al. (2022)	2975	16.60/NR/10–12	54.4	Traditional	PVS (b)	lifetime	Self	Warmth	NRI	Child	Portugal	POOR
								Aversiveness	NRI			
Gan et al. (2023)	699	14.18/12–17/NR	53.6	Cyber	E-VS	1 week	Self	Withdrawal	CPANS	Child	China	POOR
Garaigordobil and Machimbarrena (2017)	1938	10.68/9–13/5–6	49.8	Traditional, cyber	CB:SPH	12/12	Self	Authoritarian style	FEPIS	Child	Spain	POOR
								Authoritative style	FEPIS	Child		
								Warmth	PPCS	Parent		
								Permissive style	FEPIS	Child		
Garaigordobil and Navarro (2022)	3026	14.39/12–18/7–10	51.5	Cyber	CB:SPH	12	Self	Warmth	ESPA29	Child	Spain	FAIR
								Aversiveness	ESPA29			
Georgiou (2008a)	377	11.6/NR/6	48.54	Traditional	OBVQ-R	2	Self	Over-involvement	PIS	Parent	Cyprus	FAIR
								Warmth	PSQ	Child		
Georgiou (2008b)	252	11.5/NR/4–6	53.96	Traditional	OBVQ-R	4	Self	Over-involvement	PIS	Parent	Cyprus	POOR
Georgiou et al. (2013)	231	13.01/NR/1-3^**b**^	52.20	Traditional	OBVQ-R	NR	Self	Authoritarian style	PAQ	Child	Cyprus	FAIR
Georgiou and Stavrinides (2013)	492	14.2/13–15/NR	53	Traditional	OBVQ-R	NR	Self	Aversiveness	CPRS	Parent	Cyprus	FAIR
								Monitoring	P-Monitor	Parent		
Georgiou et al. (2018)	985	10.96/10–12/5–6	55.70	Traditional	OBVQ-R	NR	Self	Authoritarian style	PAQ	Child	Cyprus & Greece	FAIR
								Authoritative style	PAQ			
								Permissive style	PAQ			
Gofin and Avitzour (2012)	1884	NR/12–14/7–9	40.07	Traditional, cyber	HBSC	< 12	Self	Warmth	HBSC	Child	Israel	POOR
Goldberg et al. (2023)	91	8/4–11/k-6^**c**^	48.4	Traditional	SDQ	NR	Parent	Authoritative style	CANS	Clinician	Canada	POOR
Goodfellow et al. (2023)	5286	13.56/11–15/NR	51.3	Traditional	HBSC	NR	Self	Warmth	HBSC	Child	Scotland	POOR
Goswami (2011)	4673	13.69/NR/8–10	53	Traditional	LIKERT SCALE	12	Self	Warmth	MSLLS	Child	UK	POOR
Gullone and Robertson (2008)	241	13.8/12–16/NR	57.83	Traditional	PRQ	12	Self	Inter-parental conflict	FES	Child	Australia	POOR
Han et al. (2021)	38,292	NR/15/7–12	49.26	Traditional	developed by PISA	12	Self	Warmth	Developed by PISA	Child	China, Japan, South Korea, Taiwan & US	POOR
Havewala and Wang (2021)	425	10.59/8–12/3–6	43.1	Traditional	DBVS	NR	Self	Warmth	EMBU	Child	China	FAIR
								Aversivenss	EMBU			
Herráiz and Gutiérrez (2016)	769	14.13/13–17/2-3^**d**^	46	Traditional	PVS	NR	Self	Warmth	The Kidscreen-52	Child	Spain	POOR
Healy and Sanders (2014)	215	8.72/6–12/NR	39	Traditional	TKD	< 1 week (self version)	Self, teacher	Authoritative style	FPS	Parent	Australia	GOOD
Healy et al. (2015a)	185	7.65/5–11/prep-5^**e**^	50.20	Traditional	BBR + TKD	1 WEEK (CHILD REPORT)	Self, teacher	Autonomy granting	FPS	Parent	Australia	POOR
								Aversiveness	PS			
								Over-involvement	FPS			
								Permissive style	PS			
								Warmth	FPS			
Healy et al. (2015b)	185	7.65/5–11/prep-5	50.20	Traditional	BBR	NR	Teacher	Authoritative style	FES	Parent	Australia	POOR
Heerde et al. (2018)	2906	13.63/12–15/7	51	Traditional	IYDS	NR	Self	Inter-parental conflict	YIDS	Child	Australia & USA	FAIR
								Monitoring	YIDS			
								Warmth	YIDS			
Hellheldt et al. (2019)	1707	11.89/10–13/NR	47.50	Cyber	OBVQ	6	Self	Warmth	MSPSS	Child	Sweden	POOR
Hokoda et al. (2006)	325	NR/13–16/1-3^**f**^	58	Traditional	CSEQ	1	Self	Authoritarian style	PSDQ	Parent	Taiwan	POOR
								Authoritative style	PSDQ			
								Aversiveness	PSDQ			
								Over-involvement	PCRQ			
								Warmth	PSDQ			
Holfeld & Baitz (2020)	1151	12.7/10–16/NR	48.60	Cyber	CVS (b)	< 3	Self	Warmth	MSPSS	Child	USA	POOR
Hong et al. (2016)	7533	14.33/NR/6–10	51.50	Traditional, cyber	HBSC	NR/NR	Self	Monitoring	HBSC	Child	USA	POOR
								Warmth	HBSC			
Hong et al. (2020)	8996	12.9/11–15/5–10	50.70	Traditional	HBSC	2	Self	Monitoring	HBSC	Child	USA	POOR
								Warmth	HBSC			
Hong et al. (2021a)	1438	NR/11–15/5–10	49.10	Traditional	HBSC	2	Self	Authoritarian style	HBSC	Child	USA	POOR
								Authoritative style	HBSC			
								Monitoring	HBSC			
								Permissive style	HBSC			
								Warmth	HBSC			
Hong et al. (2021b)	3792	13 /10–17/5–10	49.2	Traditional	HBSC	2	Self	Monitoring	HBSC	Child	USA	POOR
Hong et al. (2023)	12,092	12.98/NR/5–10	52.25	Traditional	HBSC	2	Self	Warmth	HBSC	Child	USA	POOR
Hsieh (2020)	853	12.89/NR/5–9	49	Cyber	CBQ	6	Self	Aversiveness	PCS-YRSR	Child	Taiwan	FAIR
								Warmth	CRPBI + PSDQ			
Ilevbare et al. (2023)	356	14.77/10–17/1-3^**g**^	55.1	Cyber	CBS	NR	Self	Authoritative style	PSS (a)	Child	Nigeria	POOR
Ioannidou and Zafiropoulou (2021)	374	12.04/10–14/5–6 & 1-2^**h**^	53.5	Traditional	OBVQ-R	1	Self	Warmth	PBI	Child	Cyprus	POOR
								Over-involvement	PBI			
Jiang et al. (2021)	728	16.33/15–18/10–12	52.6	Cyber	items by Varjas et al. (2009)	NR	Self	Authoritative style	APQ	Child	China	POOR
Jutengren et al. (2011)	880	13.72/13–15/7–8	49.43	Traditional	Alsaker and Brunner (1999) scale	< 12	Self	Aversiveness	PRFAP	Child	Sweden	FAIR
Katz et al. (2019)	180	13.25/12–14.5/7–8	49.44	Cyber	items by Walrave and Heirman (2011) + CBI	NR	Self	Authoritative style	P-PASS	Child	Israel	FAIR
								Authoritarian style	P-PASS			
Kelly et al. (2008)	322	10.9/6–16/NR	NR	Traditional	BTQ	NR	Parent	Inter-parental conflict	FES	Parent	Australia	POOR
								Warmth	FES			
Kim and Kim (2019)	584	NR/NR/3–6	50.17	Traditional	OBVQ	1 WEEK	Self	Withdrawal	PARQ	Child	Korea	FAIR
Klomek et al. (2015)	1294	NR/NR/7–8	54.60	Traditional	items based on Sourander et al. (2010)	6	Self	Warmth	ASSS	Child	Israel	POOR
Kokkinos and Panayiotou (2007)	186	10.63/9–13/NR	50	Traditional	BVQ	NR	Self	Authoritarian style	Demetriou et al., (2000) scale	Parent	Cyprus	POOR
								Authoritative style	Demetriou et al., (2000) scale			
								Inconsistent discipline	Demetriou et al., (2000) scale			
Kokkinos (2013)	601	NR/10–12/5–6	50.74	Traditional	BVS	1 WEEK	Self	Aversiveness	EMBU	Child	Greece	POOR
								Warmth	EMBU			
Kokkinos et al. (2016)	220	NR/10–12/5–6	51.40	Cyber	CBVEQ	1	Self	Autonomy granting	PSQ	Child	Greece	POOR
								Monitoring	PSQ + SIPQ			
								Warmth	PSQ			
Košir et al. (2023)	1925	15.35/NR/9 & 1–3^**i**^	57.5	Traditional	APRI-BT	< 12	Self	Warmth	DAP	Child	Slovenia	POOR
Krisnana et al. (2021)	705	NR/13–18/NR	46.10	Traditional	APRI	NR	Self	Authoritarian style	PSDQ	Child	Indonesia	POOR
								Authoritative style	PSDQ			
								Aversiveness	PACHIQ-R			
								Permissive style	PSDQ			
								Warmth	PACHIQ-R			
Lardier et al. (2016)	488	13.81/10–18/7–12	47.70	Traditional	SRQ	NR	Self	Inter-parental conflict	SSRPF	Child	USA	POOR
Larranga et al. (2016)	813	14.38/NR/7–10	54.60	Cyber	CBQ	6	Self	Aversiveness	PCCS	Child, parent	Spain	FAIR
								Warmth	PCCS			
								Withdrawal	PCCS			
Larsen et al. (2012)	2051	13.8/NR/7–8	48.50	Traditional	OBVQ	< 12	Self	Warmth	RSI	Child	Netherlands	FAIR
Li et al. (2019)	2969	15.48/NR/7–11	55.60	Traditioanl	OBVQ-R	NR	Self	Autonomy granting	Wang et al., (2007) scale	Child	China	POOR
								Over-involvement	Wang et al., (2007) scale			
Low and Espelage (2014)	1232	13.9/10–15/5–7	49.80	Traditional	UIVS	1	Self	Monitoring	PSS-SSDP	Child	USA	FAIR
Malecki et al. (2008)	142	NR/NR/6–8	54.90	Traditional	VQ	12	Self	Warmth	CASSS	Child	Spain	POOR
Malm et al. (2017)	142	NR/NR/4–5	NR	Traditional	SSBB-R	NR	Self	Monitoring	PSMS	Parent	USA	POOR
Malm and Henrich (2019)	828	NR/NR/3–6	49.50	Traditional	PSS-BV	NR	Self	Warmth	CPRS	Parent	USA	FAIR
Marraccini et al. (2022)	155	15.20/13–18/7–12	68.6	Traditional	APRI	NR	Self	Warmth	SCM	Child	USA	GOOD
Marret and Choo (2017)	1487	NR/15–16/NR	52.63	Cyber	items based on GUMS + YISS	12	Self	Inter-parental conflict	MPS	Child	Malaysia	FAIR
Mendez et al. (2017)	1148	14.41/11–18/NR	50.80	Traditional	PNI	NR	Peers	Authoritative style	FRIDA	Child	Spain	POOR
								Permissive style	FRIDA			
Nansel et al. (2001)	15,686	NR/NR/6–10	NR	Traditional	HBSC	< 12	Self	Monitoring	HBSC	Child	USA	POOR
Navarro et al. (2013)	1068	11.4/10–12/5–6	48.68	Cyber	IVS	6	Self	Autonomy granting	Navarro et al., (2012) scale	Child	Spain	FAIR
								Over-involvement	Navarro et al., (2012) scale			
Nozaki (2019)	1363	NR/11–15/2,4,7^**j**^	57.66	Traditional	HBSC	2	Self	Warmth	HBSC	Child	Scotland	POOR
Nuñez-Fadda et al. (2020)	1687	13.65/12–17/1-3^**k**^	54	Traditional	SVS	NR	Self	Aversiveness	PACS	Child	Mexico	FAIR
								Warmth	PACS			
Nuñez-Fadda et al. (2022)	1685	13.65/12–17/NR	54	Traditional	SVS	12	Self	Warmth	The Family APGAR	Child	Mexico	POOR
Olenik-Shemesh and Heiman (2016)	204	14.8/14–16/9–10	48	Traditional, cyber	SSQCB	12/12	Self	Warmth	MSPSS	Child	Israel	POOR
Ortega Barón et al. (2018)	849	14.09/12–18/7–9	48.30	Cyber	CYBVIC	12	Self	Withdrawal	PACS	Child	Spain	FAIR
								Warmth	PACS			
Owusu et al. (2022)	3609	14.82/13–17/7–11	49.49	Traditional	GSHS	1	Self	Monitoring	GSHS	Child	Timor-Leste	POOR
								Warmth	GSHS			
Özdemir (2014)	337	16.37/15–18/9–11	55.50	Cyber	CBI-R	NR	Self	Warmth	AFP	Child	Turkey	POOR
Papadaki and Giovazolias (2015)	201	11.23/10–12/5–6	46.80	Traditional	PEQ	NR	Self	Aversiveness	PARQ	Child	Greece	FAIR
								Warmth	PARQ			
Perasso et al. (2021)	3172	13.74/NR/6,8,10	48.40	Traditional, cyber	HBSC	2/2	Self	Warmth	MSPSS	Child	Italy	POOR
Poteat et al. (2011)	15,923	14.85/10–18/7–12	50	Traditional	UIVS	1	Self	Warmth	DCYA	Child	USA	FAIR
Ren et al. (2023)	2445	12.98/NR/7	48.3	Traditional	OBVQ	< 6	Self	Aversivenss	PCQ	Child	China	FAIR
								Monitoring	PCQ			
Rinaldi et al. (2023)	225	12.74/NR/7–8	60	Traditional	PRQ	NR	Self	Warmth	PSS (b) + PCCS	Child	Canada	POOR
								Aversiveness	PSS (b)			
								Monitoring	PSS (b)			
Rose et al.(2015)	443	12.9/11–15/7–8	46.7	Traditional	UIVS	1	Self	Warmth	VSSR	Child	USA	POOR
Rothon et al. (2011)	2688	NR/11–14/7,9	51.40	Traditional	RELACHS	< 12	Self	Warmth	MSPSS	Child	UK	POOR
Russo et al. (2021)	307	10/9–12/4–5	52.80	Traditional	MPVS	NR	Self	Warmth	CASSA	Child	USA	POOR
Sarhangi et al. (2023)	233	16.2/13–18/10–12	79	Cyber	CBVEQ	NR	Self	Warmth	MSPSS	Child	Iran	POOR
Sasson and Mesch (2017)	495	13.83/10–18/6–11	46.26	Cyber	EU KIDS	12	Self	Autonomy granting	based on EU Kids	Child	Israel	POOR
								Monitoring	based on EU Kids			
Sener (2021)	142	11.08/NR/4–6	51.70	Traditional	PBS	NR	Self	Aversiveness	FRSC	Child	Turkey	POOR
								Warmth	FRSC			
Shin et al. (2014)	227	NR/NR/6	46.69	Traditional	SEQ-P + PNI	NR	Self, peers	Inter-parental conflict	CPIC	Parent	South Korea	POOR
Serra-Negra et al. (2015)	348	13.7/13–15/NR	51.72	Traditional	PeNSE	1	Self	Warmth	MLSSA	Child	Brazil	FAIR
Stavrinides et al. (2015)	348	13.5/NR/7–8	55.50	Traditional	BVQ-R	NR	Self	Monitoring	PSMS	Parent	Cyprus	FAIR
Strohmeier et al. (2022)	1018	13.55/12–17/7–8	52.3	Traditional, cyber	LIKERT SCALE/LIKERT SCALE	2/2	Self	Monitoring	Strohmeier et al. (2022) scale	Child	Austria	POOR
								Warmth	Strohmeier et al. (2022) scale			
Tang et al. (2023)	1543	8.92/6–12/1–6	48.3	Traditional	JVQ	NR	Self	Warmth	IPPA	Child	China	POOR
Tao et al. (2022)	736	NR/NR/3	52	Cyber	CAV	NR	Self	Warmth	EU Kids	Child	China	POOR
Tian et al. (2023)	4326	9.94/8–13/NR	44.6	Cyber	EBQ	2	Self	Warmth	EMBU	Child	China	GOOD
Tobias and Chapanar (2016)	62	16.5/14–18/9–12	64.51	Cyber	Hinjuda & Patchin (2010) scale	NR	Self	Warmth	READ	Child	USA	POOR
Van Hoof et al. (2008)	194	14.7/12–18/9–11	40.20	Traditional	KOP	NR	Self	Warmth	LFQ	Child	Netherlands	POOR
								Withdrawal	LFQ			
Vannucci et al. (2021)	1282	12.75/11–14/7–8	51.56	Traditional, cyber	R-PEQ + SN-PEQ	6/6	Self	Warmth	MSPSS	Child	USA	FAIR
Vargas and Monjardín (2019)	348	NR/NR/NR	NR	Traditional	APRI	NR	Self	Autonomy granting	FES	Child	Mexico	POOR
								Inter-parental conflict	FES			
								Monitoring	FES			
								Warmth	FACES III + PSS-Fa + PACS			
Varsamis et al. (2022)	532	13.18/11–16/5–6	50.18	Traditional	LIKERT SCALE	2	Self	Warmth	BPNRQ	Child	Greece	POOR
Veenstra et al.(2005)	1065	11.09/NR/NR	50.08	Traditional	PNI	NR	Peers	Warmth	EMBU	Child	Netherlands	POOR
								Over-involvement	EMBU			
								Aversiveness	EMBU			
Verešová and Mujkošová (2020)	287	13.16/NR/6–8	46.68	Traditional	VRCHA	NR	Self	Authoritarian style	R-FCPI	Child	Slovak republic	POOR
								Autonomy granting	FES + R-FCPI			
								Inter-parental conflict	FES			
								Over-involvement	FES			
								Permissive style	FES			
								Warmth	FES			
Wachs et al. (2020)	2071	13.63/12–17/6, 8	48.70	Traditional	OBVQ-R	NR	Self	Warmth	Fend & Prester (1986) scale	Child	Germany	POOR
Wang et al. (2009)	7182	NR/NR/6–10	52.20	Traditional, cyber	OBVQ	2/2	Self	Warmth	HBSC	Child	USA	POOR
Wang et al. (2019)	301,628	NR/NR/6–8	50.70	Traditional, cyber	GSHS	NR/NR	Self	Warmth	GSHS	Child	USA	POOR
Wang et al. (2022)	362,980	NR/NR/9–12	51.85	Traditional, cyber	GSHS	NR/NR	Self	Warmth	GSHS	Child	USA	POOR
Wang and Chen (2023)	11,497	15/15–16/7 and above	47.9	Traditional	developed by PISA	12	Self	Warmth	developed by PISA	Child	China	POOR
Wong and McBride (2016)	312	13.5/NR/7–8	66.34	Cyber	Leung and McBride-Chang (2013) scale	NR	Self	Warmth	MSPSS	Child	China	POOR
Wong and Konishi (2021)	422	13.85/NR/7–11	55.21	Traditional, cyber	VHBIQ	NR/NR	Self	Autonomy granting	P-PASS	Child	China	POOR
								Aversiveness	P-PASS			
Wright (2016a)	50	13.80/13–15/7–8	0	Traditional	Wright and Li (2012) scale	< 12	Self	Authoritarian style	PAQ	Child	USA	POOR
								Authoritative style	PAQ			
								Permissive style	PAQ			
Wright (2016b)	867	13.67/13–15/8	51	Traditional	Wright and Li (2012) scale	2	Self	Warmth	CASSS	Child	USA	FAIR
Wright (2017)	131	NR/13–15/8	27	Traditional, cyber	Wright and Li (2012) scale	< 12/< 12	Self	Warmth	CASSS	Child	USA	FAIR
Wright (2018)	113	13.48/12–17/7–9	14	Traditional, cyber	Wright and Li (2012) scale	< 12/< 12	Self	Warmth	CASSS	Child	USA	FAIR
Wright et al. (2021)	436	13.26/NR/7–8	51	Traditional	Wright et al. (2014) scale	< 12	Self	Authoritative style	PAQ	Child	USA	POOR
								Authoritarian style	PAQ			
								Permissive style	PAQ			
Wright and Wachs (2020)	121	14.10/13–15/8	37	Traditional, cyber	Wright and Li (2012) scale	< 12/< 12	Self	Warmth	CASSS	Child	USA	FAIR
Wu et al. (2022a)	348	9.18/7–11/NR	30.17	Traditional	OBVQ	NR	Self	Aversiveness	SPPC	Child	China	GOOD
Wu et al. (2022b)	1297	15.50/NR/NR	61.1	Cyber	CIPQ	3	Self	Autonomy granting	P-PASS	Child	China	FAIR
Wu et al. (2023a)	866	10.55/9–13/4–6	44.8	Traditional	DBVS	NR	Self	Warmth	MSPSS	Child	China	POOR
Wu et al. (2023b)	723	9.53/8–11/NR	35.81	Traditional	OBVQ	NR	Self	Warmth	MIS	Child	China	FAIR
Xiao et al. (2023)	9156	12.84/10–17/NR	43.81	Traditional	OBVQ	< 6	Self	Aversiveness	EMBU	Child	China	FAIR
								Over-involvement	EMBU			
								Warmth	EMBU			
Yabko et al. (2008)	242	12.25/11–14/7–8	61.20	Traditional	CSEQ	1	Self	Aversiveness	PPI	Child	Mexic	POOR
Ye et al. (2022)	733	NR/NR/7–9	45.7	Cyber	DBVS	NR	Self	Authoritative style	PPQ	Parent	China	POOR
								Authoritarian style	PPQ			
Yu et al. (2023)	1701	11.99/NR/4,5,7,8	47.7	Traditional	SBPVS	NR	Self	Aversiveness	PPC	Child	China	FAIR
Zhang et al. (2022a)	6247	NR/8–18/4–9	45.6	Traditional	DBVS	NR	Self	Warmth	The Family APGAR	Child	China	FAIR
Zhang et al. (2022b)	620	11.73/10–14 /4,5,7	47.8	Traditional, cyber	SBPVS/CS	NR/NR	Self	Aversiveness	PCQ	Child	China	FAIR
								Monitoring	PCQ			
Zhang et al. (2023)	12,058	NR/15/NR	47.9	Traditional	developed by PISA	12	Self	Warmth	developed by PISA	Child	China	POOR
Zhou et al. (2022a)	492	NR/NR/5–6	49.8	Traditional, cyber	DBVS	NR/NR	Self	Over-involvement	EMBU	Child	China	FAIR
								Aversiveness	EMBU			
Zhou et al. (2022b)	3743	9.92/NR/NR	46.2	Cyber	RCBI-II	2	Self	Aversiveness	FAD	Child	China	FAIR
Zhou et al. (2023)	65,868	10.83/8–14/4	46.4	Traditional	PVQ	< 6	Self	Warmth	PCRS	Child	China	FAIR
Zhou and Li (2021)	2156	15.96/NR/1-3^**l**^	56.03	Cyber	Zhou and Li (2021) scale	< 6	Self	Authoritarian style	Zhou and Li (2021) scale	Child	China	FAIR
								Permissive style	Zhou and Li (2021) scale			

^a^2nd to 3rd year of compulsory secondary education in Spain

^b^1st to 3rd year of high school in Cyprus

^c^k=kindergarter

^d^2nd to 3rd year of secondary education in Spain

^e^Prep to 5ft year of school in Australia

^f^1st to 3rd year of junior high school in Taiwan

^g^1st to 3rd year of secondary education in Nigeria

^h^1st and 2nd grade of high school in Cyprus

^i^9th year of basic school and 1st and 2nd year of secondary education in Slovenia

^j^2 and 4 of secondary education and grade 7 of primary education in Scotland

^k^1st to 3rd year of secondary school in Mexico

^l^1st to 3rd year of vocational high school in China

^1^Bullying victimization type

^2^Traditional bullying victimization/ cyberbullying victimization scale: PRQ= The Peer Relations Questionnaire (Rigby & Slee, 1993); EVAP= The Peer Victimization and Aggression Scale (Orpinas & Frankowski, 2001); HBSC= The Health Behaviour in School Aged Children Questionnaire; PEQ= The Peer Experiences Questionnaire (Vernberg et al., 1999); APRI-BT= The Adolescent Peer Relations Instrument: Bully/Target ( Parada, 2000); YRBSQ = Youth Risk Behavior Survey Questionnaire (CDC, 1989); CAHMI= The Child and Adolescent Health Measurement Initiative (2021); IBS= The Illinois Bully Scale (IBS; Espelage & Holt, 2001); CYBVIC= The Victimization Through Mobile Phone and Internet Scale (Buelga, Cava, & Musitu, 2012); SPRM/ CS= The Social Peer Rejection Measure/ Cyberbullying Scale (Menesini, 2011); Bull-S= The Bull-S test (Cerezo, 2012); EU Kids= The EU Kids Online Survey; BVQ= The Bullying and Victimization Questionnaire (Olweus, 1993), PECK= The Personal Experiences Checklist ( Hunt, Peters, & Rapee, 2012); OBVQ-R= The Bully/Victim Questionnaire-Revised (Olweus, 1993, 1996, 1997); CBVS (a)= The Cyberbullying Victimisation Scale (Chen, 2018). CBVS (B)= The California Bullying Victimization Scale (Felix et al., 2011); BVS= The Bully-Victimization Scale (Reynolds, 2003); PNI= The Peer Nomination Inventory; SSBB-R= The Student Survey of Bullying Behaviour-Revised (Varjas, Meyers & Hunt, 2006); SPVS= The Schwartz Peer Victimization Scale (Schwartz et al., 2002); ESTUDES= The Survey on Drug Use in the School Population in Spain (2016); ECIP-Q= The European Cyberbullying Intervention Project Questionnaire (Del Rey et al., 2015); CB:SPH= Cyberbullying: Screening of Peer Harassment (Garaigordobil, 2013); PISA= The Programme for International Student Assessment Questionnaire (2015); PVS (a)= The Peer Victimization Scale (Cava, Musitu & Murgui, 2007), PVS (b)= The Peer-Victimization Scale (Mynard & Joseph, 2000); E-VS= The E-victimization scale (Lam & Li, 2013); DBVS= The Delaware Bullying Victimization Scale (Bear et al., 2016); CBS= The Cyberbullying Behavior Scale (Garaigorodobil, 2015); TKD= The Things Kids Do (TKD) Bullied (Healy & Sanders, 2008b); BBR= The Brief Bullying Report (Healy & Sanders, 2008); IYDS= The International Youth Development Study Survey; CSEQ= The Children’s Self-Experiences Questionnaire-Selfa Report (Crick & Grotpeter, 1996); CVS (a)= The Cyber Victimization Scale (Arıcak et al., 2012); CVS (b)= The Cyber Victimization Scale (Patchin & Hinduja, 2010); CBQ= The Cyberbullying Questionnaire (Calvete et al., 2010); SDQ= The Strengths and Difficulties Questionnaire (Goodman, 2001); CBI= The Cyberbullying Inventory (Topçu, Erdur-Baker & Çapa-Aydin (2008); BTQ= The Bullying and Teasing Questionnaire (Kelly et al., 2008); BVQ*= The Bullying and Victimization Questionnaire (Kokkinos & Panayiotou, 2004b); CBVEQ= The Cyber-bullying/ Victimization Experiences Questionnaire (Antoniadou & Kokkinos 2013); SRQ= The School Relationships Questionnaire (Hamburger, Basile & Vivolo-Kantor, 2011); UIVS= The University of Illinois Victimization Scale (Espelage & Holt, 2001); VQ= The Victimization Questionnaire (Malecki et al., 2008); PSS-BV= The Peer Social Support- Bullying, & Victimization (NICHD, 2005); IVS= The Internet Victimization Scale (Buelga, Cava & Musitu, 2010); SVS= The School Victimization Scale (Buelga, Cava, & Musitu, 2012); SSQCB= The Student Survey Questionnaire of Cyberbullying (Campbell et al., 2012); CBI-R= The Cyberbullying Inventory- Revised (Topçu & Erdur-Baker, 2010); RELACHS= The Research with East London Adolescents: Community Health Survey (2001); MPVS= Multidimensional Peer Victimization Scale (Mynard & Joseph, 2000); PBS= The Peer Bullying Scale (Pişkin & Ayas, 2011); SEQ-P= The Social Experience Questionnaire- Peer-Report (Crick & Werner, 1998); PeNSE= The Brazilian National School-Based Adolescent Health Survey (2009); JVQ= The Juvenile Victimization Questionnaire (Chan, 2013); CAV= The Cyber-Aggression and Cyber-Victimization Scale (Shapka & Maghsoudi, 2017); EBQ= The Electronic Bullying Questionnaire (Moore et al. 2012); R-PEQ= The Revised Peer Experiences Questionnaire (Prinstein, Boergers, & Vernberg, 2001); SN-PEQ= the Social Network Peer Experiences Questionnaire (Landoll, La Greca, & Lai, 2013); VRCHA= Výskyt rizikového chování u adolescentů (Dolejš & Skopal, 2015); GSHS= The Global School-Based Student Health Survey; VHBIQ= The Vaillancourt and Hymel Bullying Involvement Questionnaire (Vaillancourt et al., 2008); CIPQ= The revised European Cyber bullying Intervention Project Questionnaire (Zhu et al., 2021); SBPVS= The School Bullying Perpetration and Victimization Scale (Zhang, 2002); CS= The Cyberbullying Scale (Wright & Li, 2013); RCBI-II= The Revised Cyberbullying Inventory (Topcu & Erdur- Baker, 2018)

^3^Traditional bullying victimization/ cyberbullying victimization time of reference (months)

^4^Bullying victimization reported by

^5^Parental factor scale: PDI-SF= The Parenting Dimensions Inventory-Short Form (Power, 2002); PAS= The Parental Attitude Scale (Lamborn et al., 1991); SSA= The Social Support Appraisals Scale (Vaux et al. 1986); HBSC= The Health Behaviour in School Aged Children Questionnaire; PFCS= The Parent–Family Connectedness Scale (Resnick et al., 1997); IPPA-R= The Inventory for Parent and Peer Attachment-Revised (Gullone & Robinson, 2005); ECR= The Experiences in Close Relationships Scale (Fraley et al., 2011); ); CAHMI= The Child and Adolescent Health Measurement Initiative (2021); IBS= The Illinois Bully Scale (IBS; Espelage & Holt, 2001); FAI= The Chinese Family Assessment Instrument (Shek, 2002); NRI= The Network of Relationships Inventory (Coimbra & Mendonça, 2013); CPANS= The Child Psychological Abuse and Neglect Scale (Deng et al., 2017); PACS= The Parent–Adolescent Communication Scale (Barnes & Olson, 1982); PQ= The Parenting Questionnaire (Statistics Canada, 1997); CRPBI-30= Children’s Report of Parent Behaviour Inventory (Schludermann & Schludermann, 1988); PES= The Parental Encouragement Scale (Cassidy & Lynn, 1991); FES= The Family Environment Scale (Fernandez-Ballesteros & Sierra, 1989); ESPA29= The Parental Socialization Scale in Adolescence (Musitu & García, 2001); CANS= The Child and Adolescent Needs and Strengths (Cordell et al., 2016); EU Kids= The EU Kids Online Survey ; PAQ= The Parental Authority Questionnaire (Buri, 1991); IPPA= The Inventory for Parent and Peer Attachment (IPPA; Armsden & Greenberg, 1987); CASSA= The Children and Adolescent Social Support Scale (Malecki et al., 2008); SERI= scale developed by Seoul Education Research & Information Institute; PPS= The Perceptions of Parents Scale (Soenens & Vansteenkiste, 2005); ESTUDES= The Survey on Drug Use in the School Population in Spain (2016); CRHS= The Caring Relationships at Home Scale (California Department of Education, 2015); CTCYS= The Communities That Care Youth Survey (Arthur et al., 2007); PSDQ= The Parenting Styles and Dimensions Questionnaire (Robinson et al., 2001); GSHS= The Global School-Based Student Health Survey; MSPSS= The Multidimensional Scale of Perceived Social Support (Zimet et al., 1988); FEPIS= The Family Educational Practices Identification Scales (Alonso & Román, 2003); PPCS= The Perceived Parental Competence Scale (Bayot & Hernández, 2008); PIS= The Parental Involvement Scale (Campbell & Mandel, 1990); CPRS= The Child–Parent Relationship Scale (Pianta 1992); P-Monitor= The P-Monitor (Stattin & Kerr, 2000); MSLLS= The Multidimensional Students’ Life Satisfaction Scale (Huebner, 1994); FPS= The Facilitative Parenting Scale (Healy & Sanders, 2008a); PS= The Parenting Scale (Rhoades & O’Leary, 2007); YIDS= Communities That Care Youth Survey; PCS-YRSR= The Psychological Control Scale-Youth Self-Report (Barber, 1996); CRPBI= The Children’s Reports of Parental Behaviour Inventory (Schaefer, 1965); PSS (a)= The Parenting Styles Scale (Bamurind, 1971); PBI= The Parental Bonding Instrument (Parker et al., 1979); APQ= The Alabama Parenting Questionnaire (Elgar et al., 2007); PRFAP= Parents’ Reactions & Feelings About Parents (Tilton-Weaver et al., 2010); P-PASS= The Perceived Parental Autonomy Support Scale (Mageau et al., 2015); PARQ= The Parental Acceptance–Rejection Questionnaire (Rohner et al., 1980); ASSS= The Attachment Security Style Scale (Kerns et al., 1996); EMBU= The Egna Minnen Betraffande Uppfostran (Muris et al., 1998); PSQ= The Parenting Styles Questionnaire (Rayner & Moore, 2007); SIPQ= The Specific Internet Practices Questionnaire (Van den Eijnden et al., 2009); DAP= The Developmental Assets Profile ( Scales, 2011); SCM= The School Climate Measure (Zullig et al., 2015); PACHIQ-R= The Parent-Child Interaction Questionnaire-Revised (Lange et al., 2002); SSRPF= The Student Survey of Risk and Protective Factors (Gorman-Smith et al., 1996); PCCS= The Parent- Child Communication Scale (Barnes & Olson, 1985); RSI= The Relational Support Inventory (Scholte et al., 2001); PSS-SSDP= The Parental Supervision Subscale- Seattle Social Development Project (Arthur et al., 2002); PSMS= The Parental Supervision and Monitoring Scale (Kerr & Stattin, 2000); MPS= The Measure of Parenting Style (Parker et al., 1997); FRIDA= The Parental Educational Styles- FRIDA – Interpersonal Risk Factors for Drug Consumption in Adolescence (Secades et al., 2006); AFP= The Adolescent Family Process (Vazsonyi et al., 2003); DCYA= The 2009 Dane County Youth Assessment; VSSR= Vaux Social Support Record ( Vaux, 1988); PCQ= The Parental Control Questionnaire (Wang et al.,2007); PSS (b)= The Parenting Style Scale (Steinberg et al., 1994); BPNRQ= The Basic Psychological Needs in Relationships questionnaire (La Guardia et al., 2000); FRSC= The Family Relationships Scale for Children; CPIC= The Children’s Perceptions of Interparental Conflict (Grych et al., 1992); MLSSA= The Multidimensional Life Satisfaction Scale for Adolescent (Huebner, 1994) ; READ= The Resilience Scale for Adolescence (Hjemdal et al., 2006); LFQ= The Leuven Family Questionnaire (Kog et al., 1985); FACES III= The Family Adaptability and Cohesion Evaluation Scale (Olson, 1985); PSS-Fa= The Perceived Social Support- Family (Procidano & Heller, 1983); R-FCPI= The Revised Family Communication Pattern Instrument (Ritchie & Fitzpatrick, 1990); GSHS= The Global School-Based Student Health Survey; PPI= The Parent Perception Inventory (Hazzard et al., 1983); SPPC= The Scale of Parental Psychological Control (Shek, 2005); MIS= The Maternal Involvement Scale (Wu et al., 2018); PPQ= The Parenting Practice Questionnaire(Robinson et al., 1995); PPC= The Parental Psychological Control Scale (Wang et al. (2007); FAD= The Family Assessment Device (Epstein et al., 1978); PCRS= The Relationships Inventory (Furman & Buhrmester; 1985)

^6^Parental factor reported by

The 158 studies included in the systematic review and meta-analysis were published between 2001 and 2023. The combined sample of all included studies consisted of 1,095,468 participants. Of those, 50.6% were girls. Five studies did not report on the gender of their sample, and one study had a sample consisting only of boys. Based on 119 studies, the mean age was 12.95 years. Studies that did not report the mean age provided either the age range (4 to 18 years old) or the grade the students were in (kindergarten to grade 12), except for one study. Out of the 158 included studies, 109 reported associations with traditional bullying victimization, 30 assessed associations with cyberbullying victimization, and 19 reported associations with both. 89 studies indicated a time frame of reference for bullying victimization that varied from 1 week (4 studies) to 12 months (16 studies). Additionally, one study assessed lifetime bullying victimization. Bullying victimization was self-reported in most of the studies. Five studies measured bullying victimization by peer nominations, and one study used both peer nominations and self-report measures. One study used exclusively teacher reports, and two studies used both teacher and self-reports to assess bullying victimization. Only one study assessed bullying victimization through parent reports. The most analyzed parental factor was warmth (reported in 110 studies), followed by aversiveness (reported in 30 studies). On the other hand, only one study reported on the association between inconsistent discipline and bullying victimization, and no study reporting on the association between encouraging sociability and bullying victimization was found. In 19 studies, the parental factor was reported by parents themselves. One study assessed the parental factor through both child and parent reports, and another study assessed the parental factor through clinician reports. The remaining studies relied on child-report measurements (see Table 1).

### Quality Assessment of Included Studies (Fig. 2, Table A.1)

**Fig. 2:**
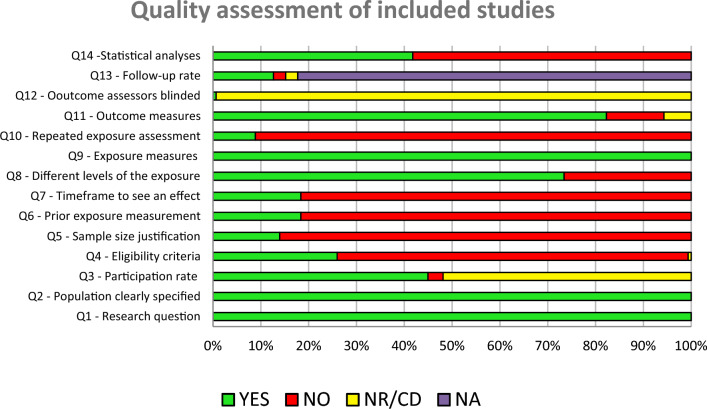
Quality assessment of included studies (N = 158)

Out of the 158 studies that reported on the association between parental factors and traditional as well as cyberbullying victimization, 102 were rated as having “inadequate” quality, 50 were rated as having “fair” quality, and 6 were rated as having “good” quality. The most frequent caveats were the lack of sample size justification (N = 136), not specifying the inclusion and exclusion criteria (N = 116), and not controlling for confounding variables (N = 92). Given that most studies were cross-sectional, exposure was not repeatedly assessed (N = 144), nor assessed prior to the outcome (N = 129), therefore, there was no sufficient time to see an effect (N = 129) (see Fig. 2 and Table A.1). The inter-rater reliability for the overall quality of the studies was high (Kappa = 0.88).

### Main Effects and Sensitivity Analyses

#### Parental Protective Factors and Traditional Bullying Victimization (Table 2, Fig. 3)

The effect sizes of the association between each parental factor and traditional bullying victimization are presented in Table 2. The overall association between parental protective factors and traditional bullying victimization was small with a large level of heterogeneity (k = 111, r = − .12, 95% CI [− .14; − .09], I^2^ = 99). When sensitivity analyses were performed by excluding the outliers (k = 71, r = -.12, 95% CI [− .13; − .11], I^2^ = 82) or by considering only the studies with good and fair quality (k = 37, r = − .12, 95% CI [− .17; − .07], I^2^ = 99), the overall effect size remained unchanged, and the level of heterogeneity decreased only in the case of good and fair quality studies. The effect size of the association between authoritative parenting (k = 13, r = 10,95% CI [− .18; − .02], I^2^ = 85), warmth (k = 92, r = − .14, 95% CI [− .17; − .12], I^2^ = 99), and monitoring (k = 17, r = − .06, 95% CI [− .11; − .02], I^2^ = 88), respectively, and traditional bullying victimization was small and significant with high levels of heterogeneity. Small and significant effect size was also found for the association between autonomy granting and traditional bullying victimization but with low levels of heterogeneity (k = 8, r = − .16, 95% CI [− .20; − .12], I^2^ = 25). All the associations were negative, meaning that the more parents exhibit warmth, autonomy granting, monitoring, or authoritative parenting, the less likely their children are to be victims of traditional bullying.

**Table 2:** Effect sizes of the associations between parental protective and risk factors and traditional bullying victimization

Outcome	k	N	*r*^*a*^	LCI	UCI	*I*^*2*^	LCI	UCI
Parental protective factors	111	711 776	*− 0.12*	*− 0.14*	*− 0.09*	99	99	99
Excluding outliers^b^	71	610 094	*− 0.12*	*− 0.13*	*− 0.11*	82	84	99
FAIR and GOOD quality studies^c^	37	140 280	*− 0.12*	*− 0.17*	*− 0.07*	99	98	99
Authoritative parenting	13	7 129	*− 0.10*	*− 0.18*	*− 0.02*	85	75	90
Autonomy granting	8	6 556	*− 0.16*	*− 0.20*	*− 0.12*	25	0	66
Monitoring	17	63 649	*− 0.06*	*− 0.11*	*− 0.02*	88	82	92
Warmth	92	995 897	*− 0.14*	*− 0.17*	*− 0.12*	99	98	99
Parental risk factors	55	73 314	*0.19*	*0.17*	*0.22*	82	78	86
Excluding outliers^d^	41	66 693	*0.21*	*0.19*	*0.22*	41	67	83
FAIR and GOOD quality studies^c^	20	29 572	*0.21*	*0. 19*	*0.24*	81	72	87
Authoritarian parenting	12	7 149	*0.14*	*0.07*	*0.21*	81	68	89
Aversiveness	27	50 170	*0.20*	*0.16*	*0.23*	78	68	85
Inter-parental conflict	7	4 819	*0.21*	*0.14*	*0.29*	68	29	86
Over-involvement	15	25 791	*0.17*	*0.11*	*0.23*	83	73	89
Permissive parenting	9	6 450	*0.12*	*0.03*	*0.20*	86	76	92
Withdrawal	5	2 158	*0.18*	*0.09*	*0.28*	72	30	89

*k* number of studies, *N* number of participants, *LCI* lower confidence interval, *UCI* upper confidence interval,* I*^*2*^ percentage of heterogeneity, *NA* not applicable

^a^All results are reported with r correlation (significant results are marked with italic)

^b^Outliers were defined as studies in which the 95% CI was outside the 95% CI of the pooled studies: Alcantara et al. (2017), Alikasifoglu et al. (2007), Arabiat et al. (2018), Bartolomé Gutiérrez and Díaz Herráiz (2019), Beran and Violato (2004), Boniel-Nissim and Sasson (2018), Charalampous et al. (2019), Cho and Norman (2019), Cho et al. (2019), Elledge et al. (2019), Fanti et al. (2012), Garaigordobil and Machimbarrena (2017), Georgiou et al. (2018), Gofin et al. (2012), Havewala et al. (2021), Healy et al. (2015a, b), Heerde et al. (2019), Herraiz et al. (2016), Hong et al. (2021a), Ioannidou et al. (2021), Kokkinos and Panayiotou (2007), Krisnana et al. (2021), Li et al. (2019), Marraccini et al. (2022), Olenik-Shemesh et al. (2017), Owusu et al. (2022), Papadaki and Giovazolias (2015), Rinaldi et al. (2023), Serra-Negra et al. (2015), Stavinidre et al. (2015), Strohmeier et al. (2022), Tang et al. (2023), Vannucci et al. (2021), Vargas and Monjardín (2019), Veenstra et al. (2005), Wang et al. (2019), Wright (2018), Wright et al. (2021), Zhang et al. (2022a), Zhou et al. (2023)

^c^Fair and good studies were defined as studies which had less than 50% (respectively 75% for good quality) risk of bias accordingly to NIH Quality Assessment Tool for Observational Cohort and Cross-Sectional Studies NIH (2014) detailed in Table A.1

^d^Beran and Violato (2004), Chen et al. (2022), Chui et al. (2022), Garaigordobil and Machimbarrena (2017), Georgiou et al. (2018), Hokada et al. (2006), Hong et al. (2021a), Ioannidou et al. (2021), Kokkinos and Panayiotou (2007), Krisnana et al. (2021), Lardier et al. (2016), Papadaki and Giovazolias (2015), Veenstra et al. (2005), Xiao et al. (2023)

**Fig. 3:**
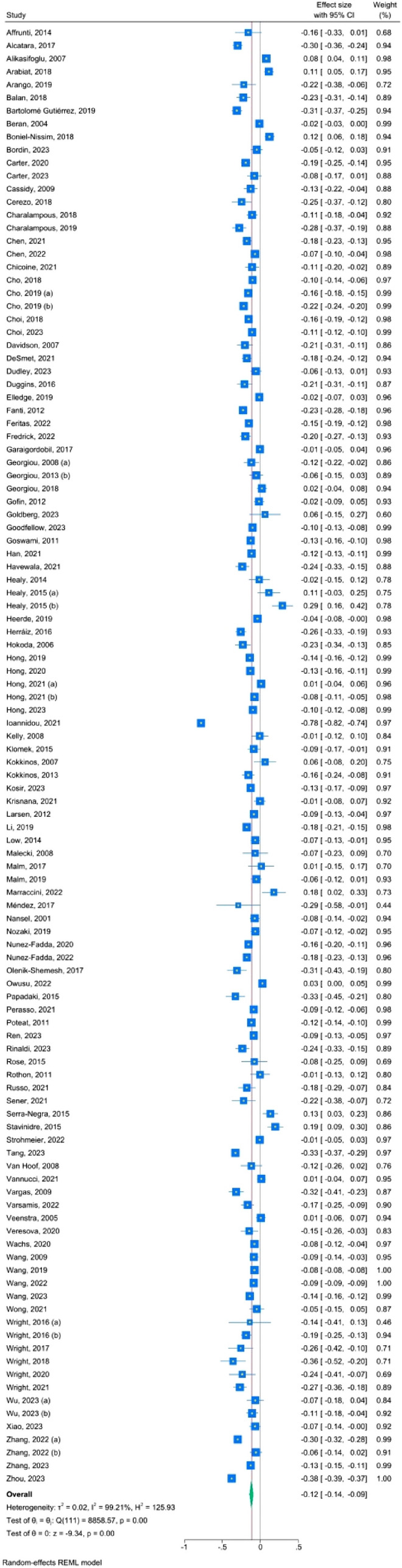
Forest plot—parental protective factors and traditional bullying victimization

#### Parental Risk Factors and Traditional Bullying Victimization (Table 2, Fig. 4)

The overall effect size of the association between parental risk factors and traditional bullying victimization was small and with a high level of heterogeneity (k = 55, r = .19, 95% CI [.17; .22], I^2^ = 82). After removing the outliers, the effect size slightly increased and the heterogeneity level decreased to a moderate level (k = 41, r = .21, 95% CI [.19; .22], I^2^ = 41). When we considered only the studies with fair and good quality, the effect size also increased, but the level of heterogeneity remained high (k = 20, r = .21, 95% CI [.19; .24], I^2^ = 81). A significant and small effect size was found for the association between authoritarian parenting (k = 12, r = .14, 95% CI [.07; .21], I^2^ = 81), aversiveness (k = 27, r = .20, 95% CI [.16; .23], I^2^ = 78), over-involvement (k = 15, r = .17, 95% CI [.11; .23], I^2^ = 83), and permissive parenting (k = 9, r = 0.12, 95% CI [.03; .20], I^2^ = 86), respectively, and traditional bullying victimization with a high level of heterogeneity. A significant and small effect size was also found for the association with inter-parental conflict (k = 7, r = .21, 95% CI [.14; .29], I^2^ = 68) and parental withdrawal (k = 5, r = .18, 95% CI [.09; 0.28], I^2^ = 72), but with moderate to high levels of heterogeneity. All the effect sizes were positive, meaning that the more parents exhibit authoritarian parenting, aversiveness, inter-parental conflict, over-involvement, permissive parenting, and withdrawal, respectively, the more likely it is for their children to be victims of traditional bullying.

**Fig. 4:**
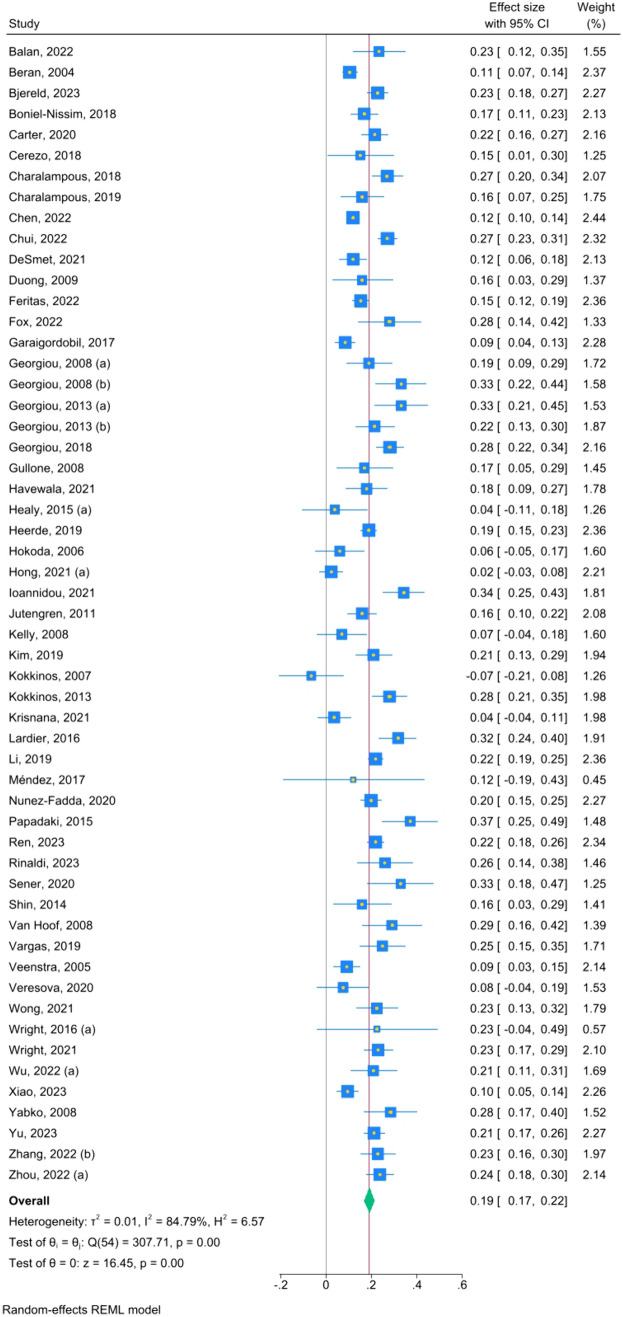
Forest plot—parental risk factors and traditional bullying victimization

#### Parental Protective Factors and Cyberbullying Victimization (Table 3, Fig. 5)

The effect sizes of the association between each parental factor and cyberbullying victimization are presented in Table 3. The overall effect size of the association between parental protective factors and cyberbullying victimization was small (k = 45, r = − .11, 95% CI [− .15; − .08], I^2^ = 99), accompanied by a high level of heterogeneity. When outliers were excluded, the effect size was similar and the level of heterogeneity remained high (k = 31, r = − .11, 95% CI [− .13; − .09], I^2^ = 96). When only studies with fair and good quality were considered, the effect size and the heterogeneity level decreased, although slightly (k = 15, r = − .10, 95% CI [− .17; − .04], I^2^ = 88). A small and significant effect size was found for the association between warmth (k = 36, r = − .14, 95% CI [− .17; − .10], I^2^ = 99) and cyberbullying victimization, with a high level of heterogeneity. The association was negative, meaning that the more parents exhibit warmth, the less likely it is for their children to become victims of cyberbullying victimization. No significant association was found between authoritative parenting (k = 5, r = .01, 95% CI [− .07; .09], I^2^ = 70), autonomy granting (k = 7, r = − .09, 95% CI [− .21; .03], I^2^ = 92), and monitoring (k = 7, r = − .04, 95% CI [− .10; .02], I^2^ = 89), respectively, and cyberbullying victimization.

**Table 3:** Effect sizes of the associations between parental protective and risk factors and cyberbullying victimization

Outcome	k	N	r^a^	LCI	UCI	I^2^	LCI	UCI
Parental protective factors	45	756 960	*− 0.11*	*− 0.15*	*− 0.08*	99	99	99
Excluding outliers^b^	31	706 263	*− 0.11*	*− 0.13*	*− 0.09*	96	58	87
FAIR and GOOD quality studies^c^	15	17 132	*− 0.10*	*− 0.17*	*− 0.04*	88	82	92
Authoritative parenting	5	3 946	0.01	*− *0.07	0.09	70	24	88
Autonomy granting	7	5 089	*− *0.09	*− *0.21	0.03	92	87	96
Monitoring	7	14 370	*− *0.04	*− *0.10	0.02	89	80	94
Warmth	36	751 321	*− 0.14*	*− 0.17*	*− 0.10*	99	99	99
Parental risk factors	21	24 734	*0.16*	*0.10*	*0.21*	95	94	96
Excluding outliers^d^	14	16 849	*0.15*	*0.12*	*0.18*	66	56	88
FAIR and GOOD quality studies^c^	13	17 510	*0.17*	*0.10*	*0.24*	94	91	96
Authoritarian parenting	5	4 724	0.23	*− *0.05	0.50	97	95	98
Aversiveness	9	10 662	*0.17*	*0.14*	*0.21*	80	63	89
Over-involvement	5	5 421	0.05	*− *0.03	0.12	85	66	93
Permissive parenting	2	4 235	0.01	*− *0.16	0.15	88	NA	NA
Withdrawal	2	4 312	*0.14*	*0.09*	*0.19*	0	NA	NA

*k* number of studies, *N* number of participants, *LCI* lower confidence interval, *UCI* upper confidence interval,* I*^*2*^ percentage of heterogeneity, *NA* not applicable

^a^All results are reported with r correlation (significant results are marked with italic)

^b^Outliers were defined as studies in which the 95% CI was outside the 95% CI of the pooled studies: Chang et al. (2015), Escario et al. (2023), Espino et al. (2023), Garaigordobil and Machimbarrena (2017), Holfeld and Baitz (2020), Katz et al. (2019), Navarro et al. (2013), Strohmeier et al. (2023), Tao et al. (2023), Tian et al. (2023), Tobias et al. (2016), Vannucci et al. (2020), Wright (2017), Zhang et al. (2022b)

^c^Fair and good studies were defined as studies which had less than 50% (respectively 75% for good quality) risk of bias accordingly to NIH Quality Assessment Tool for Observational Cohort and Cross-Sectional Studies NIH (2014), detailed in Tabel A.1

^d^Chang et al. (2015), Charalampous et al., (2018), Garaigordobil and Machimbarrena (2017), Katz et al. (2019), Navarro et al. (2013); Ye et al. (2023), Zhou et al. (2021)

**Fig. 5:**
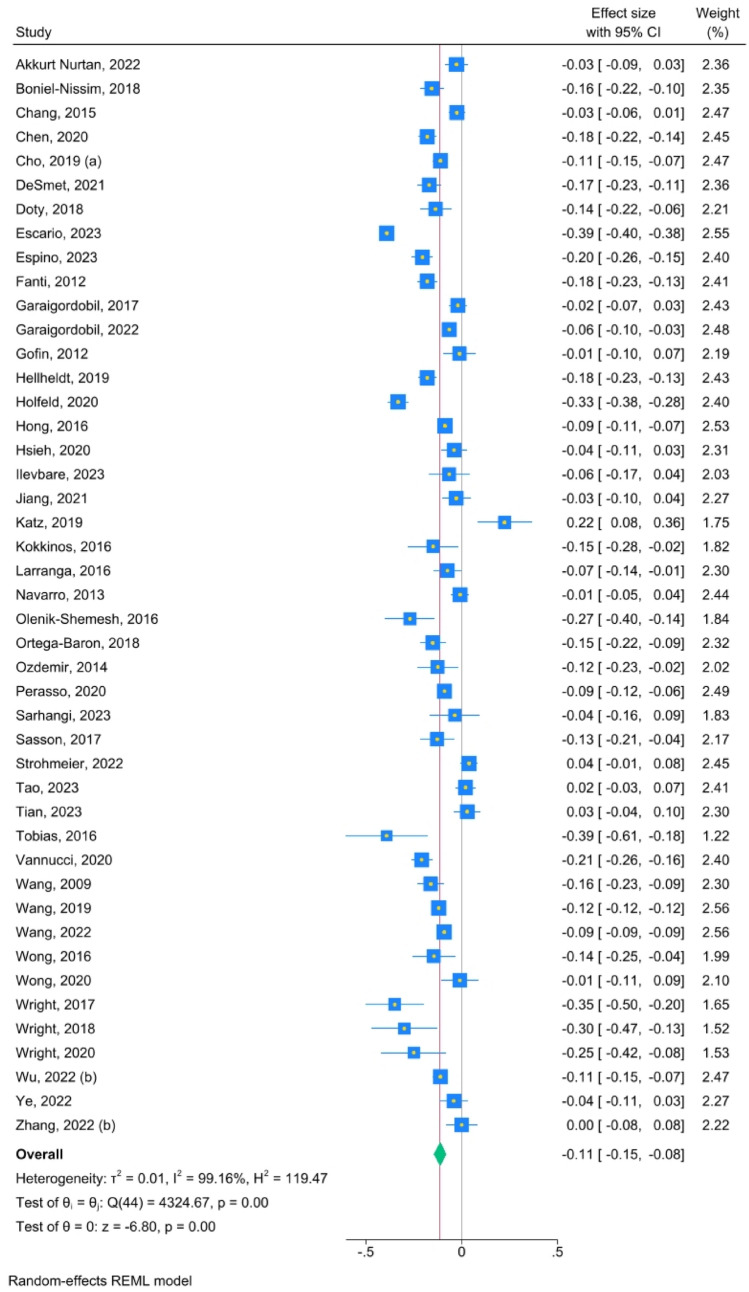
Forest plot—parental protective factors and cyberbullying victimization

#### Parental Risk Factors and Cyberbullying Victimization (Table 3, Fig. 6)

The overall effect size of the association between parental risk factors and cyberbullying victimization was small and with a high level of heterogeneity (k = 21, r = .16, 95% CI [.10; .21], I^2^ = 95). When outliers were excluded, both the effect size and the level of heterogeneity decreased (k = 14, r = .15, 95% CI [.12; .18], I^2^ = 66). However, when only studies with fair and good quality were considered, the effect size slightly increased, and the level of heterogeneity was similar to the initial value (k = 13, r = .17, 95% CI [.10; .24], I^2^ = 94). A small and significant effect size was found for the association between parental aversiveness (k = 9, r = .17, 95% CI [.14; .21], I^2^ = 80) and cyberbullying victimization, with a high level of heterogeneity. Similarly, a small and significant effect size was found for the association between parental withdrawal (k = 2, r = .14, 95% CI [.09;.19]) and cyberbullying victimization. The effect sizes were positive, meaning that the more parents exhibit aversiveness and withdrawal, the more likely it is for their children to be bullied in the online context. No significant association was found between authoritarian parenting (k = 5, r = .23, 95% CI [− .05; .50], I^2^ = 97), permissive parenting (k = 2, r = .01, 95% CI [− .16; .15], I^2^ = 88), and parental over-involvement (k = 5, r = .05, 95% CI [− .03; .12], I^2^ = 85), respectively, and cyberbullying victimization.

**Fig. 6:**
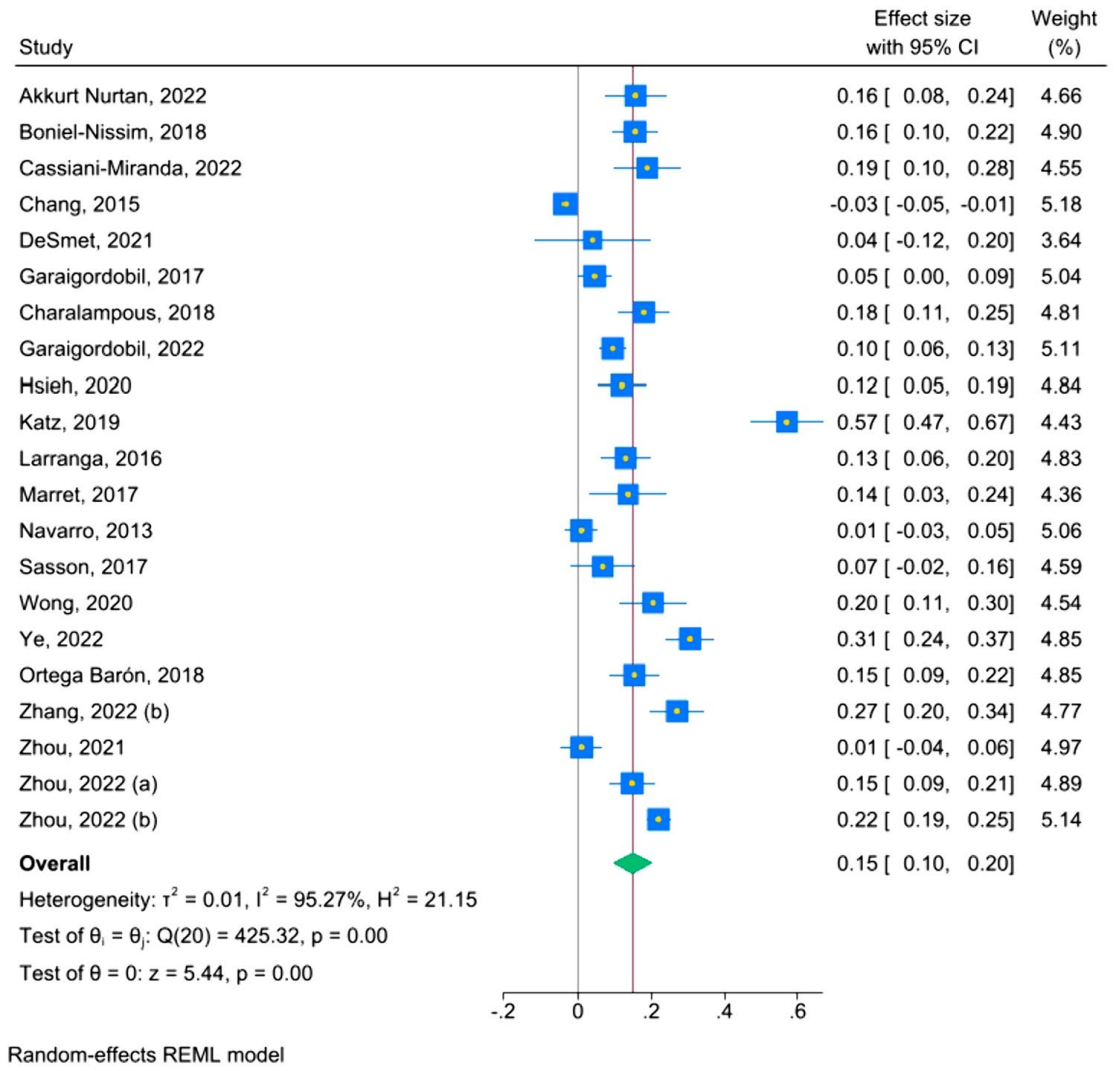
Forest plot—parental risk factors and cyberbullying victimization

### Maternal and Paternal Factors and Traditional/Cyberbullying Victimization

Small and significant effect sizes were found when we considered the differential association between maternal (k = 24, r = − .12, 95% CI [− .19; − .05], I^2^ = 98) and paternal (k = 11, r = − .14, 95% CI [− .24; − .03], I^2^ = 98) protective factors and traditional bullying victimization, with high levels of heterogeneity in both cases. Similarly, small and significant associations were found between maternal (k = 20, r = .21, 95% CI [.18; .25], I^2^ = 78) and paternal (k = 9, r = .17, 95% CI [.10; .23], I^2^ = 91) risk factors and traditional bullying victimization, with high levels of heterogeneity. Furthermore, small and significant associations were identified between maternal (k = 6, r = − .09, 95% CI [− .14; − .05], I^2^ = 69) and paternal (k = 6, r = − .08, 95% CI [− .12; − .04], I^2^ = 60) protective factors and cyberbullying victimization, with moderate to high levels of heterogeneity in each case. Finally, small and significant associations were found between maternal (k = 3, r = .16, 95% CI [.07; .24], I^2^ = 80) and paternal (k = 3, r = .13, 95% CI [.08; .17], I^2^ = 39) risk factors and cyberbullying victimization, with high and low to moderate levels of heterogeneity, respectively.

### Meta-regression Analysis

Meta-regression analysis indicated that the association between parental protective factors and traditional bullying victimization was not significantly moderated by the percentage of females (β = .002, p = .236) or by the mean age (β = − .002, p = .728). Similarly, regarding the effect size of the association between parental risk factors and traditional bullying victimization, neither the percentage of females (β = .002, p = .339) nor the mean age (β = .−002, p = .798) was a significant predictor. The effect size of the association between parental protective factors and cyberbullying victimization was also not significantly moderated by the percentage of females (β = .003, p = .104), but was significantly moderated by the mean age (β = − .02, p = .029). Finally, neither the percentage of females (β = .002, p = .444) nor the mean age (β = − .008, p = .491) was a significant predictor of the effect size of the association between parental risk factors and cyberbullying victimization.

### Publication Bias

Regrading the studies examining the association between parental protective factors and traditional bullying victimization, the Egger test indicated an estimated slope of βb_1_ = 0.28, with a standard error of 0.529, giving a test statistic of z = 0.52 and a p-value of 0.602, indicating no signs of publication bias. A similar result was found for studies that examined the association between parental risk factors and traditional bullying victimization: the Egger test indicated an estimated slope of βb_1_ = 0.39, with a standard error of 0.574, giving a test statistic of z = 0.69 and a p-value of 0.492. Likewise, for studies that examined the association between parental protective factors and cyberbullying victimization, the Egger test indicated an estimated slope of βb_1_ = − 0.77, with a standard error of 0.808, giving a test statistic of z = − 0.95 and a p-value of 0.341. Furthemore, for studies that assessed the association between parental risk factors and cyberbullying victimization, the Egger test indicated an estimated slope of βb_1_ = 2.65, with a standard error of 1.945, giving a test statistic of z = 1.36 and a p-value of 0.172, suggesting no evidence of small study effects. These results were consistent with the visual inspection of the funnel plots and the contour-enhanced funnel plots, which indicated no asymmetry.

## Discussion

The role of parental factors in bullying victimization has been previously documented in several systematic reviews (Elsaesser et al., 2017; Nocentini et al., 2019) and meta-analyses (e.g., Chen et al., 2017; Cook et al., 2010; Guo, 2016; Kowalski et al., 2014; Lereya et al., 2013; Ward et al., 2018). However, these syntheses have focused either on traditional or cyberbullying victimization, often assessing the role of distinct parental factors for each, or on both, treating them as interchangeable phenomena. Therefore, a research question that arises is whether the same set of parental factors is concurrently associated with both types of bullying victimization. The present meta-analysis aimed to fill this gap in the literature by exploring the role of modifiable parental factors in relation to bullying victimization occurring in the school context and using technology. Specifically, it primarily aimed to determine which parental factors are protective, which are those that may put children at risk for traditional and cyberbullying victimization, and the magnitude of these associations.

### The Effects of Parental Factors on Traditional and Cyberbullying Victimization

Overall, results indicated significant and small associations between the broader categories of parental risk and protective factors and traditional and cyberbullying victimization, suggesting that parental factors do matter regardless of the context in which bullying victimization occurs. When we narrowed the glance and looked upon specific parental dimensions and styles, results indicated that parental warmth, autonomy granting, authoritative parenting, and monitoring were all protective factors against traditional bullying victimization. Still, for cyberbullying victimization, only parental warmth emerged as a protective factor. Furthermore, parental aversiveness, withdrawal, over-involvement, authoritarian parenting, permissive parenting, and inter-parental conflict were identified as predisposing factors for being bullied at school. In contrast, for cyberbullying victimization, only parental aversiveness and withdrawal were found to be significant risk factors. These results point to the ongoing debate over whether traditional bullying and cyberbullying are similar or distinct phenomena, with a focus on their shared or unique relationship with predictor and outcome factors. One perspective argues that cyberbullying is just an extension of traditional bullying, sharing similar features and correlates (e.g., Casas et al., 2013), while the other perspective emphasizes their distinct characteristics and related factors (e.g., Barlett et al., 2024). Our findings support the latter perspective, as they depict few commonalities and many differences in how parental factors are related to traditional and cyberbullying victimization. Specifically, all parental factors examined were significantly related to being bullied in the school context, and only three parental factors emerged as related predictors of cyberbullying victimization: warmth, aversiveness, and withdrawal. These parental factors represent distinct dimensions of the same construct (i.e., parental rejection); therefore, it is not surprising that each of them was a significant related predictor. This pattern of results could also be seen in the remaining parental categories: parental control (i.e., autonomy granting and over-involvement) and parenting styles (i.e., authoritative, authoritarian, and permissive). Specifically, all the dimensions and styles within these broader categories were all significantly related to traditional but not to cyberbullying victimization, acting like a cohesive set of parental factors. This could be due to the association between the dimensions or styles within each of these parental categories (e.g., DeSmet et al., 2021; Ye et al., 2022; Wright, 2016), as they represent different, and sometimes opposite (e.g., over-involvement and autonomy granting), facets of the same construct.

Our results revealed that only the dimensions of parental rejection were relevant in protecting or putting children at risk of being bullied in the online context and are in line with those previously reported in a systematic review, where parental warmth was shown to be a protective factor (Elsaesser et al., 2017), and with those reported in a recent meta-analysis, where parental aversiveness, in the form of parental offensive communication, was found to be a risk factor (Lozano-Blasco et al., 2023). A parent child-relationship characterized by a warmth and affection may create an environment in which children are more likely to disclose bullying incidents (Liu et al., 2020). At the same time, within such enviroment parents also have the means to guide their children in navigating online social interactions. On the other hand, from a compensatory perspective, children who feel rejected by their parents may find their comfort in the online world, which, in turn, could lead to unhealthy Internet use and an increased risk of cyberbullying victimization (Bonniel-Nissim & Sasson, 2018). However, except for the facets of parental rejection, all other parental factors investigated were found to be unrelated to cyberbullying victimization. Although these results might seem unexpected, they could suggest that cyberbullying victimization is related to other contextual factors. It is possible that parents face more challenges in addressing cyberbullying victimization given its several distinct characteristic, such as anonymity or easiness to spread among a wider audience. Furthermore, it has been reported that victims of cyberbullying tend to hide online incidents from their parents more than those of traditional bullying, as they fear they could lose their autonomy and Internet privilages (Agatston et al., 2007; Dooley et al., 2010). Therefore, parents who have limited knowledge of their child’s online experiences are deprived of the chance to intervene and offer help. In light of this possible explanation, a particularly surprising result would appear to be the non-linear association between parental monitoring (i.e., knowledge of child activities, whereabouts, and friends) and cyberbullying victimization, as opposed to the negative and significant association reported by Kowalski et al. (2014) in a previous meta-analysis. In interpreting our finding, it is important to consider that the majority of the included studies relied on measures that assessed children’s perception of parental knowledge or parental control as a way to gain knowledge and not children’s voluntary disclosure. This could be relevant since it has been reported in a meta-analysis that child disclosure is the strongest predictor of parental knowledge, while parental monitoring, in the form of parental solicitation or control, is a marginal source (Liu et al., 2020).

Taken together, our results suggest that parents may have a greater impact on bullying victimization occurring in the offline context. All parental factors directed at the child (i.e., parental rejection, control, parenting styles, and monitoring) and the relationship between parents (i.e., inter-parental conflict) were relevant in protecting or putting children at risk of being bullied at school. Of these, the dimensions of parental rejection have been previously examined through a meta-analysis, showing that parental warmth, in the form of communication and trust, reduced the risk of being bullied, while parental rejection, in the form of alienation, increased the risk (Ward et al., 2018). In the present meta-analysis, parental risk factors had slightly higher associations (ranging from .12 to .21) than parental protective factors (ranging from − .06 to − .16), suggesting that negative influences may have a stronger impact than positive ones. However, associations were small, indicating that parental factors are likely to have an indirect effect on bullying victimization through more proximal factors. In the bullying victimization literature, factors linking parenting to bullying victimization are more often inferred, and studies testing specific mechanisms are relatively scarce, although they could offer valuable insight into the pathways through which bullying victimization occurs.

Existing empirical findings have indicated that parental rejection dimensions act as risk or protective factors in bullying victimization primarily through child’s emotional difficulties (Kaufman et al., 2020; Shin et al., 2016), regulation skills (Chen et al., 2022; Samper-García et al., 2021), and bullying perpetration (Kaufman et al., 2020), while parental control (i.e., over-involvement) impacts the risk of being bullied through child’s self-control (Li et al., 2015) and basic psychological need satisfaction (Peng et al., 2023). Furthermore, parenting styles (i.e., authoritative and authoritarian) have been shown to indirectly predict bullying victimization through peer alienation (Charalampous et al., 2018) or locus of control (Georgiou et al., 2017). Parental monitoring has been found to predict academic performance, risky peer influence, and school belongingness, which, in turn, predict bullying victimization (Wu et al., 2024). It is worth noting that parental factors directed at the child and child’s bullying victimization experiences are likely to have transactional associations through child’s emotional and behavioral difficulties. For instance, Kaufman et al. (2020) have found longitudinal spillover effects from bullying victimization to parental rejection via children’s social anxiety, depressive symptoms, conduct problems, and bullying perpetration. This is concerning since the spillover effect may get children stuck in a pattern of negative interactions. Furthermore, it is generally hypothesized that children exposed to inter-parental conflicts learn negative patterns of interaction through observation and replicate them in their peer context. Empirical findings indeed suggest that children living in high-conflict homes are likely to exhibit lower social competence (Azam & Hanif, 2011), which further predisposes them to bullying victimization (Cook et al., 2010). Inter-parental conflicts may also leave children with elevated levels of anxiety and depression (Yap et al., 2014), which make them easy targets for bullies (Christina et al., 2021; Reijntjes et al., 2010).

### The Impact of Maternal and Paternal Factors on Bullying Victimization

The second objective was to examine whether maternal and paternal (i.e., risk and protective) factors are differently associated with bullying victimization (i.e., traditional and cyber). Overall, our results indicated that the practices of both parents impact a child’s risk of being bullied and are supported by previous studies that have reported similarities in how mothers’ and fathers’ relationships with their children influence traditional (e.g., Chen et al., 2022; Freitas et al., 2022) or cyberbullying victimization (e.g., Larrañaga et al., 2016; Garaigordobil & Navarro, 2022). Furthermore, our results indicated that maternal and paternal factors were common predictors of traditional and cyberbullying victimization, showing associations of similar magnitude. These results confirm previous findings that investigated the associations between parental factors and bullying victimization while taking into account parents’ gender and the type of bullying victimization (e.g., Boniel-Nissim & Sasson, 2018; Wong & Konishi, 2021).

### The Impact of Age and Gender on the Main Effects

We also examined whether age and gender had a moderating effect on the association between parental factors (i.e., risk and protective) and bullying victimization (i.e., traditional and cyber). Our results indicated that gender did not moderate the main effects, suggesting that parental factors may equally impact boys’ and girls’ risk of being bullied. Furthermore, our findings indicated that age was not a significant moderator, except for the association between parental protective factors and cyberbullying victimization, which became weaker as age increased. These findings are consistent with those reported in previous meta-analyses. Specifically, Guo (2016) found no moderating effect of age and gender for the association between negative home environment and cyberbullying victimization, and Cook et al. (2010) found no moderating effect of age for the association between positive home environment and traditional bullying victimization. The negative age effect on the association between parental protective factors and cyberbullying victimization is concordant with our expectations, since older children tend to seek independence from their parents (Levpušček, 2006). However, the non-significant moderating effect of age for the remaining associations was surprising. It is possible that the cumulative effects of parenting across time make older children equally likely to experience bullying victimization as their younger counterparts, especially in the offline context.

### Implications

This is the first meta-analysis that examined the concurrent impact of multiple parental factors on traditional and cyberbullying victimization, as well as the differential impact of maternal and paternal factors on bullying victimization (i.e., traditional and cyber). From a theoretical standpoint, these results could be used to better understand the role of parents in bullying victimization among children and adolescents. First, our findings indicated that all parental factors examined were significantly associated with traditional bullying victimization, suggesting the greater influence parents have upon bullying victimization occurring in the offline context. Second, our findings indicated few commonalities between traditional and cyberbullying victimization, challenging the extension perspective, which assumes cyberbullying is just another form of bullying with similar correlates. Third, our findings indicated that fathers were as likely as mothers to impact a child’s risk of being bullied.

From a methodological point of view, our meta-analysis demonstrated that the conceptual model of Yap et al. (2014) used as a framework for our data was suitable for exploring multiple facets of parenting in relation to bullying. Specifically, only two parental factors (i.e., inconsistent discipline and encouraging sociability) out of eleven did not seem to be represented either in the traditional or in the cyberbullying victimization literature. However, this could be due to our inclusion and exclusion criteria (e.g. validated measures for parental factors) that limited the number of included studies.

From a practical point of view, the present findings could shape the current practices used in developing anti-bullying programs. Most of the prevention and/or intervention programs follow the paradigm of ”one size fits all” and, in general, their efficacy is modest in the most optimistic cases (e.g., Gaffney et al., 2019a, 2019b), highlighting that not all children benefit from a universal approach. In addition, there is evidence showing that children who display high levels of internalizing symptoms and poor parent–child relationships report the lowest level of bullying victimization decrease after such interventions (Kaufman et al., 2018). Therefore, a personalized approach could better fit children’s needs. The current results revealed key parental factors that could serve as screening variables for creating customized interventions. However, due to the generally small effect sizes, we advise against fully incorporating parents into these programs. Instead, we recommend including targeted modules for parents to improve the overall effectiveness of interventions. Therefore, for children facing bullying at school, we suggest including modules to educate parents about the importance of a warm and supportive family environment where children feel comfortable sharing their experiences and seeking help when overwhelmed. Modules could also emphasize parental encouragement of children autonomy that is appropriate to their developmental stage and parental behaviors that convey a proper balance between warmth and control. Promoting parental practices that meet the particular needs of children (i.e., warmth and autonomy) would strengthen their resilience in the face of bullying incidents. Furthermore, for those dealing with online bullying, we propose modules that emphasize recognizing and accepting children’s behaviors that are appropriate to their developmental stage, as well as modules that target motivating parents’ active involvement and understanding of their children online activities. Additionally, we suggest that these modules be made accessible to both mothers and fathers, given that our findings indicated no noticeable difference between parents' impact on bullying victimization.

### Limitations

The present meta-analysis has several limitations. First, the results were based on cross-sectional data, thus no conclusion related to the direction and causality could be drawn. While parental factors have mostly been conceptualized as predictors of bullying victimization, it is also possible that bullied children elicit specific parental behaviors, as suggested by several longitudinal studies (e.g., Kaufman et al., 2020; Peng et al., 2023; Stavrinides et al., 2018). Second, when interpreting the results regarding non-shared predictors, it is important to consider that the primary available data was larger for traditional bullying victimization, leading to more stable effect sizes compared to those that were observed for cyberbullying victimization. In contrast, the most frequently studied parental factors in relation to both types of bullying victimization were the shared dimensions of parental rejection (i.e., warmth and aversiveness), yielding more reliable results. Additionally, no effect size could be computed for inter-parental conflict and cyberbullying victimization due to the lack of primary available data; therefore, no comparison could be made in regard to this dimension. Third, the majority of included studies examining maternal and paternal factors measured facets of parental rejection (i.e., warmth and aversiveness). Interpreting these findings beyond this dimension should be made with caution. Fourth, the included studies mostly had samples drawn from the community population of children and adolescents, and we did not perform separate analyses for those having emotional and behavioral difficulties (e.g., anxiety disorders, conduct disorder, ADHD) or developmental disabilities (e.g., autism) that might increase vulnerability to bullying victimization. This could be a topic to consider in future systematic reviews and meta-analyses. Finally, most of the analyses were accompanied by high heterogeneity between studies that could not be reduced through sensitivity analyses or explained by the proposed moderators (i.e., age and gender). The high level of heterogeneity could be due to the variety of measures used to evaluate both bullying victimization and parental factors. Furthermore, the included studies had samples consisting mainly of preadolescents and adolescents; therefore, it is possible there was not enough variability in ages (i.e., fewer children under ten years old) to detect significant age effects.

## Conclusions

Despite the mentioned limitations, this meta-analysis examined for the first time the differential impact of multiple parental factors on traditional and cyberbullying victimization. Based on the amount of primary available data, stronger evidence was found for the association between parental risk (i.e., authoritarian parenting, aversiveness, inter-parental conflict, over-involvement, permissive parenting, and withdrawal) and protective (i.e., authoritative parenting, autonomy granting, warmth, and monitoring) factors, respectively, and traditional bullying victimization. Of these, only parental warmth, aversiveness, and withdrawal were significantly related to cyberbullying victimization. We believe the effectiveness of interventions could be increased by tailoring parent-focused components based on a prior assessment of these factors. Furthermore, this meta-analysis was the first to examine the differential impact of maternal and paternal factors on traditional and cyberbullying victimization. Our findings indicated that mothers and fathers were equally likely to protect or put children at risk of being bullied, thus parents should foster a positive parent–child relationship while minimizing negative parent–child interactions.

## Supplementary Information

Below is the link to the electronic supplementary material.

Supplementary file1 (DOCX 200 kb)

## Declarations

### Review Protocol Registration Number

PROSPERO reference number CRD42021240629. Available from https://www.crd.york.ac.uk/PROSPERO/display_record.php?RecordID=240629
