# Supplementary material for: Parental Risk and Protective Factors Associated with Bullying Victimization in Children and Adolescents: A Systematic Review and Meta-analysis
Source: Clin Child Fam Psychol Rev. 2024 May 8;27(3):627–57. doi: 10.1007/s10567-024-00473-8 (PMC11486818; doi:10.1007/s10567-024-00473-8)
Supplement: Supplementary file 1 — Supplementary file1 (DOCX 200 kb) [file 10567_2024_473_MOESM1_ESM.docx]

**Appendix A** – Parental factors definitions

| **Broad category** | **Sub-dimension** | **Definition (Yap et al., 2014)** |
| --- | --- | --- |
| Parental rejection | warmth | a sense of positive regard expressed by the parent toward the child, pleasant interactions between parent and child, or parental involvement in child's activities |
|  | aversiveness | parental hostility toward the child in the form of criticism, punishment, and conflict, which reflect a lack of parental acceptance |
|  | withdrawal | the lack of parental involvement and interest in child’s activities, or the lack of emotional support/reciprocity |
| Parental control | autonomy granting | parental encouragement of child's opinions and choices, acknowledgment of child’s independent perspectives on issues, and solicitation of the child's input on decisions and solutions to difficulties |
|  | over-involvement | parental interference with child's age normative autonomy and emotional independence and encourage excessive dependence. It includes psychological control, the degree to which a parent uses coercive, hostile and controlling techniques to intrude upon their child's psychological experiences |
| Parenting styles | authoritative | the extent to which parents make rules that respect their child's maturity and independence, and convey the rationale for these rules to them |
|  | authoritarian | the degree to which a parent is highly directive, expects complete obedience from their child, and employs punitive measures to manage their behavior |
|  | permissive | the degree to which a parent allows their child to engage in activities which would normally be regulated by an adult |
|  | inconsisent | parent’s inconsistent enforcement of rules and consequences |
| Parental monitoring | **-** | parental knowledge of their child's activities, whereabouts, and friends |
| Inter-parental conflict | **-** | the frequency and intensity of inter-parental conflict, marital discontent, and the extent to which parents express hostility towards each other |
| Encouraging sociability | **-** | the extent to which parents encourage their child to engage in social behavior by modeling or verbal encouragement |

**Appendix B –** List of included studies (N=158)

Affrunti, N. W., Geronimi, E. M. C., & Woodruff-Borden, J. (2014). Temperament, Peer Victimization, and Nurturing Parenting in Child Anxiety: A Moderated Mediation Model. *Child Psychiatry & Human Development*, *45*(4), 483–492. <https://doi.org/10.1007/s10578-013-0418-2>

Akkurt Nurtan, K., Evgin, D., & Gördeles Beşer, N. (2022). The Relationship Between Internet Addiction, Cyberbullying and Parental Attitudes. *The Journal of Pediatric Research*, *9*(3), 274–285. <https://doi.org/10.4274/jpr.galenos.2022.65392>

Alcantara, S. C., González-Carrasco, M., Montserrat, C., Viñas, F., Casas, F., & Abreu, D. P. (2017). Peer violence in the School Environment and Its Relationship with Subjective Well-Being and Perceived Social Support Among Children and Adolescents in Northeastern Brazil. *Journal of Happiness Studies*, *18*(5), 1507–1532. <https://doi.org/10.1007/s10902-016-9786-1>

Alikasifoglu, M., Erginoz, E., Ercan, O., Uysal, O., & Albayrak-Kaymak, D. (2007). Bullying behaviours and psychosocial health: Results from a cross-sectional survey among high school students in Istanbul, Turkey. *European Journal of Pediatrics*, *166*(12), 1253–1260. <https://doi.org/10.1007/s00431-006-0411-x>

Arabiat, D. H., Shaheen, A., Nassar, O., Saleh, M., & Mansour, A. (2018). Social and Health Determinants of Adolescents’ Wellbeing in Jordan: Implications for Policy and Practice. *Journal of Pediatric Nursing*, *39*, 55–60. <https://doi.org/10.1016/j.pedn.2017.03.015>

Arango, A., Cole-Lewis, Y., Lindsay, R., Yeguez, C. E., Clark, M., & King, C. (2019). The Protective Role of Connectedness on Depression and Suicidal Ideation Among Bully Victimized Youth. *Journal of Clinical Child & Adolescent Psychology*, *48*(5), 728–739. <https://doi.org/10.1080/15374416.2018.1443456>

Balan, R., Dobrean, A., & Balazsi, R. (2018). Indirect effects of parental and peer attachment on bullying and victimization among adolescents: The role of negative automatic thoughts. *Aggressive Behavior*, *44*(6), 561–570. <https://doi.org/10.1002/ab.21775>

Balan, R., Dobrean, A., & Balazsi, R. (2022). From Victims to Perpetrators of Bullying: The Role of Irrational Cognitions, Externalizing Problems, and Parental Attachment. *Journal of Interpersonal Violence*, *37*(19–20), NP19149–NP19166. <https://doi.org/10.1177/08862605211043583>

Bartolomé Gutiérrez, R., & Díaz Herráiz, E. (2019). Apoyo social y autopercepción en los roles del acoso escolar. *Anales de Psicología*, *36*(1), 92–101. <https://doi.org/10.6018/analesps.301581>

Beran, T. N., & Violato, C. (2004). A Model of Childhood Perceived Peer Harassment: Analyses of the Canadian National Longitudinal Survey of Children and Youth Data. *The Journal of Psychology*, *138*(2), 129–148. <https://doi.org/10.3200/JRLP.138.2.129-148>

Bjereld, Y., Augustine, L., Turner, R., Löfstedt, P., & Ng, K. (2023). The association between self-reported psychosomatic complaints and bullying victimisation and disability among adolescents in Finland and Sweden. *Scandinavian Journal of Public Health*, *51*(8), 1136–1143. <https://doi.org/10.1177/14034948221089769>

Boniel-Nissim, M., & Sasson, H. (2018). Bullying victimization and poor relationships with parents as risk factors of problematic internet use in adolescence. *Computers in Human Behavior*, *88*, 176–183. <https://doi.org/10.1016/j.chb.2018.05.041>

Bordin, I. A., & Handegård, B. H. (2023). Factors Associated with Peer Victimization Among Brazilian Low-Income Adolescents. *Journal of Aggression, Maltreatment & Trauma*, *32*(11), 1511–1530. <https://doi.org/10.1080/10926771.2023.2203078>

Carter, M., van der Watt, R., & Esterhuyse, K. (2020). The relationship between perceived parenting dimensions, attachment, and pre-adolescent bullying. *Journal of Psychology in Africa*, *30*(2), 106–118. <https://doi.org/10.1080/14330237.2020.1744280>

Carter, M., Van Der Watt, R., & Esterhuyse, K. (2023). Parent and peer attachment in bullying experiences among pre-adolescents. *Journal of Psychology in Africa*, *33*(1), 26–34. <https://doi.org/10.1080/14330237.2023.2182948>

Cassiani-Miranda, C. A., Campo-Arias, A., & Caballero-Domínguez, C. C. (2022). Factors Associated with Cyberbullying Victimisation among Colombian High-School Adolescents. *Journal of Child & Adolescent Trauma*, *15*(1), 27–36. <https://doi.org/10.1007/s40653-021-00355-z>

Cassidy, T. (2009). Bullying and victimisation in school children: The role of social identity, problem-solving style, and family and school context. *Social Psychology of Education*, *12*(1), 63–76. <https://doi.org/10.1007/s11218-008-9066-y>

Cerezo, F., Ruiz-Esteban, C., & Sánchez, C. (2018). Dimensions of parenting styles, social climate, and bullying victims in primary and secondary education. *Psicothema*, *30.1*, 59–65. <https://doi.org/10.7334/psicothema2016.360>

Chang, F.-C., Chiu, C.-H., Miao, N.-F., Chen, P.-H., Lee, C.-M., Chiang, J.-T., & Pan, Y.-C. (2015). The relationship between parental mediation and Internet addiction among adolescents, and the association with cyberbullying and depression. *Comprehensive Psychiatry*, *57*, 21–28. <https://doi.org/10.1016/j.comppsych.2014.11.013>

Charalampous, K., Demetriou, C., Tricha, L., Ioannou, M., Georgiou, S., Nikiforou, M., & Stavrinides, P. (2018). The effect of parental style on bullying and cyber bullying behaviors and the mediating role of peer attachment relationships: A longitudinal study. *Journal of Adolescence*, *64*(1), 109–123. <https://doi.org/10.1016/j.adolescence.2018.02.003>

Charalampous, K., Ioannou, M., Georgiou, S., & Stavrinides, P. (2019). The Integrative Model of Multiple Attachment Relationships in Adolescence: Linkages to Bullying and Victimization. *International Journal of Developmental Science*, *13*(1–2), 3–17. <https://doi.org/10.3233/DEV-180249>

Chen, J.-K. (2020). Cyber victimisation, social support, and psychological distress among junior high school students in Taiwan and Mainland China. *Asia Pacific Journal of Social Work and Development*, *30*(3), 150–163. <https://doi.org/10.1080/02185385.2020.1755994>

Chen, J.-K., Wang, S.-C., Chen, Y.-W., & Huang, T.-H. (2021). Family Climate, Social Relationships With Peers and Teachers at School, and School Bullying Victimization Among Third Grade Students in Elementary Schools in Taiwan. *School Mental Health*, *13*(3), 452–461. <https://doi.org/10.1007/s12310-020-09404-8>

Chen, X., Lu, J., Ran, H., Che, Y., Fang, D., Chen, L., Peng, J., Wang, S., Liang, X., Sun, H., & Xiao, Y. (2022). Resilience mediates parenting style associated school bullying victimization in Chinese children and adolescents. *BMC Public Health*, *22*(1), 2246. <https://doi.org/10.1186/s12889-022-14746-w>

Chicoine, J., Marcotte, D., & Poirier, M. (2021). Bullying perpetration and victimization among adolescents: A diathesis-stress model of depressive symptoms. *Journal of Applied Developmental Psychology*, *77*, 101350. <https://doi.org/10.1016/j.appdev.2021.101350>

Cho, S., & Lee, J. M. (2018). Explaining physical, verbal, and social bullying among bullies, victims of bullying, and bully-victims: Assessing the integrated approach between social control and lifestyles-routine activities theories. *Children and Youth Services Review*, *91*, 372–382. <https://doi.org/10.1016/j.childyouth.2018.06.018>

Cho, S., & Norman, L. (2019). The Mediating Effect of Social Controls on Marijuana Use Among Adolescent Bullies, Victims, and Bully-Victims: A Comparison of Various Approaches to Mediation. *Substance Use & Misuse*, *54*(5), 796–810. <https://doi.org/10.1080/10826084.2018.1543326>

Cho, S., Lee, H., Peguero, A. A., & Park, S. (2019). Social-ecological correlates of cyberbullying victimization and perpetration among African American youth: Negative binomial and zero-inflated negative binomial analyses. *Children and Youth Services Review*, *101*, 50–60. <https://doi.org/10.1016/j.childyouth.2019.03.044>

Choi, B., & Park, S. (2018). What Do Parents Attribute the Cause of Bullying to? It Can Amplify or Buffer the Vicious Cycle of Bullying Perpetration and Victimization. *Journal of Interpersonal Violence*, *33*(23), 3589–3609. <https://doi.org/10.1177/0886260518778265>

Choi, J. (2023). The Moderating Role of Family Resilience in the Relationship of Bullying Victimization, Adverse Childhood Experiences, and Poverty Status to Adolescents’ Internalizing Disorders. *Journal of Aggression, Maltreatment & Trauma*, *32*(5), 745–762. <https://doi.org/10.1080/10926771.2022.2074329>

Chui, W. H., Weng, X., & Khiatani, P. V. (2022). Associations among Bullying Victimization, Family Dysfunction, Negative Affect, and Bullying Perpetration in Macanese Adolescents. *International Journal of Offender Therapy and Comparative Criminology*, *66*(1), 28–49. <https://doi.org/10.1177/0306624X20983741>

Davidson, L. M., & Demaray, M. K. (2007). Social Support as a Moderator Between Victimization and Internalizing–Externalizing Distress From Bullying. *School Psychology Review*, *36*(3), 383–405. <https://doi.org/10.1080/02796015.2007.12087930>

DeSmet, A., Rodelli, M., Walrave, M., Portzky, G., Dumon, E., & Soenens, B. (2021). The Moderating Role of Parenting Dimensions in the Association between Traditional or Cyberbullying Victimization and Mental Health among Adolescents of Different Sexual Orientation. *International Journal of Environmental Research and Public Health*, *18*(6), 2867. <https://doi.org/10.3390/ijerph18062867>

Doty, J., Gower, A., Sieving, R., Plowman, S., & McMorris, B. (2018). Cyberbullying Victimization and Perpetration, Connectedness, and Monitoring of Online Activities: Protection from Parental Figures. *Social Sciences*, *7*(12), 265. <https://doi.org/10.3390/socsci7120265>

Dudley, M. J., Nickerson, A. B., Seo, Y. S., & Livingston, J. A. (2023). Mother-Adolescent Agreement Concerning Peer Victimization: Predictors and Relation to Coping. *Journal of Child and Family Studies*, *32*(10), 3134–3147. <https://doi.org/10.1007/s10826-023-02567-3>

Duggins, S. D., Kuperminc, G. P., Henrich, C. C., Smalls-Glover, C., & Perilla, J. L. (2016). Aggression among adolescent victims of school bullying: Protective roles of family and school connectedness. *Psychology of Violence*, *6*(2), 205–212. <https://doi.org/10.1037/a0039439>

Duong, M. T., Schwartz, D., Chang, L., Kelly, B. M., & Tom, S. R. (2009). Associations between Maternal Physical Discipline and Peer Victimization among Hong Kong Chinese Children: The Moderating Role of Child Aggression. *Journal of Abnormal Child Psychology*, *37*(7), 957–966. <https://doi.org/10.1007/s10802-009-9322-4>

Elledge, L. C., Smith, D. E., Kilpatrick, C. T., McClain, C. M., & Moore, T. M. (2019). The associations between bullying victimization and internalizing distress, suicidality, and substance use in Jamaican adolescents: The moderating role of parental involvement. *Journal of Social and Personal Relationships*, *36*(7), 2202–2220. <https://doi.org/10.1177/0265407518786804>

Escario, J.-J., Rodriguez-Sanchez, C., Sancho-Esper, F., & Barlés-Arizón, M.-J. (2023). A quantitative analysis of factors related to adolescent cybervictimization in Spain: A multilevel logistic regression approach. *Children and Youth Services Review*, *155*, 107170. <https://doi.org/10.1016/j.childyouth.2023.107170>

Espino, E., Guarini, A., & Del Rey, R. (2023). Effective coping with cyberbullying in boys and girls: The mediating role of self-awareness, responsible decision-making, and social support. *Current Psychology*, *42*(36), 32134–32146. <https://doi.org/10.1007/s12144-022-04213-5>

Fanti, K. A., Demetriou, A. G., & Hawa, V. V. (2012). A longitudinal study of cyberbullying: Examining riskand protective factors. *European Journal of Developmental Psychology*, *9*(2), 168–181. <https://doi.org/10.1080/17405629.2011.643169>

Fox, J. K., Ryan, J. L., Martin Burch, J., & Halpern, L. F. (2022). The Role of Parental Overcontrol in the Relationship between Peer Victimization, Social Threat Cognitions, and Social Anxiety in School-Age Children. *School Mental Health*, *14*(1), 201–212. <https://doi.org/10.1007/s12310-021-09466-2>

Fredrick, S. S., Nickerson, A. B., & Livingston, J. A. (2022). Family cohesion and the relations among peer victimization and depression: A random intercepts cross-lagged model. *Development and Psychopathology*, *34*(4), 1429–1446. <https://doi.org/10.1017/S095457942100016X>

Freitas, D. F. D., Mendonça, M., Wolke, D., Marturano, E. M., Fontaine, A. M., & Coimbra, S. (2022). Resilience in the face of peer victimization and perceived discrimination: The role of individual and familial factors. *Child Abuse & Neglect*, *125*, 105492. <https://doi.org/10.1016/j.chiabu.2022.105492>

Garaigordobil, M., & Machimbarrena, J. M. (2017). Stress, competence, and parental educational styles in victims and aggressors of bullying and cyberbullying. *Psicothema*, *29.3*, 335–340. <https://doi.org/10.7334/psicothema2016.258>

Garaigordobil, M., & Navarro, R. (2022). Parenting Styles and Self-Esteem in Adolescent Cybervictims and Cyberaggressors: Self-Esteem as a Mediator Variable. *Children*, *9*(12), 1795. <https://doi.org/10.3390/children9121795>

Georgiou, S. N. (2008a). Bullying and victimization at school: The role of mothers. *British Journal of Educational Psychology*, *78*(1), 109–125. <https://doi.org/10.1348/000709907X204363>

Georgiou, S. N. (2008b). Parental style and child bullying and victimization experiences at school. *Social Psychology of Education*, *11*(3), 213–227. <https://doi.org/10.1007/s11218-007-9048-5>

Georgiou, S. N., & Stavrinides, P. (2013). Parenting at home and bullying at school. *Social Psychology of Education*, *16*(2), 165–179. <https://doi.org/10.1007/s11218-012-9209-z>

Georgiou, S. N., Fousiani, K., Michaelides, M., & Stavrinides, P. (2013). Cultural value orientation and authoritarian parenting as parameters of bullying and victimization at school. *International Journal of Psychology*, *48*(1), 69–78. <https://doi.org/10.1080/00207594.2012.754104>

Georgiou, S. N., Ioannou, M., & Stavrinides, P. (2018). Cultural values as mediators between parenting styles and bullying behavior at school. *Social Psychology of Education*, *21*(1), 27–50. <https://doi.org/10.1007/s11218-017-9413-y>

Gofin, R., & Avitzour, M. (2012). Traditional Versus Internet Bullying in Junior High School Students. *Maternal and Child Health Journal*, *16*(8), 1625–1635. <https://doi.org/10.1007/s10995-012-0989-8>

Goldberg, B. J., Smith, J. D., Whitley, J., & Rogers, M. (2023). Bullying Involvement among Children Receiving Clinical Care: Links to Mental Health Indicators, Individual Strengths, and Parenting Challenges. *Child & Youth Services*, *44*(2), 105–121. <https://doi.org/10.1080/0145935X.2022.2040358>

Goodfellow, C., Willis, M., Inchley, J., Kharicha, K., Leyland, A. H., Qualter, P., Simpson, S., & Long, E. (2023). Mental health and loneliness in Scottish schools: A multilevel analysis of data from the health behaviour in school‐aged children study. *British Journal of Educational Psychology*, *93*(2), 608–625. <https://doi.org/10.1111/bjep.12581>

Goswami, H. (2012). Social Relationships and Children’s Subjective Well-Being. *Social Indicators Research*, *107*(3), 575–588. <https://doi.org/10.1007/s11205-011-9864-z>

Gullone, E., & Robertson, N. (2008). The relationship between bullying and animal abuse behaviors in adolescents: The importance of witnessing animal abuse. *Journal of Applied Developmental Psychology*, *29*(5), 371–379. <https://doi.org/10.1016/j.appdev.2008.06.004>

Han, Y., Kang, H. R., Choe, J. W., & Kim, H. (2021). The moderating role of parental support in the relationship between latent profiles of bullying victimization and sense of school belonging: A cross-national comparison. *Children and Youth Services Review*, *122*, 105827. <https://doi.org/10.1016/j.childyouth.2020.105827>

Havewala, M., & Wang, C. (2021). Effects of Peer Victimization on Child Emotional and Behavioral Difficulties: The Moderating Role of Parenting. *Journal of Child and Family Studies*, *30*(11), 2601–2615. <https://doi.org/10.1007/s10826-021-02054-7>

Healy, K. L., & Sanders, M. R. (2014). Randomized Controlled Trial of a Family Intervention for Children Bullied by Peers. *Behavior Therapy*, *45*(6), 760–777. <https://doi.org/10.1016/j.beth.2014.06.001>

Healy, K. L., Sanders, M. R., & Iyer, A. (2015a). Parenting Practices, Children’s Peer Relationships and Being Bullied at School. *Journal of Child and Family Studies*, *24*(1), 127–140. <https://doi.org/10.1007/s10826-013-9820-4>

Healy, K. L., Sanders, M. R., & Iyer, A. (2015b). Facilitative Parenting and Children’s Social, Emotional and Behavioral Adjustment. *Journal of Child and Family Studies*, *24*(6), 1762–1779. <https://doi.org/10.1007/s10826-014-9980-x>

Heerde, J. A., Curtis, A., Bailey, J. A., Smith, R., Hemphill, S. A., & Toumbourou, J. W. (2019). Reciprocal associations between early adolescent antisocial behavior and depressive symptoms: A longitudinal study in Victoria, Australia and Washington State, United States. *Journal of Criminal Justice*, *62*, 74–86. <https://doi.org/10.1016/j.jcrimjus.2018.09.003>

Hellfeldt, K., López-Romero, L., & Andershed, H. (2019). Cyberbullying and Psychological Well-being in Young Adolescence: The Potential Protective Mediation Effects of Social Support from Family, Friends, and Teachers. *International Journal of Environmental Research and Public Health*, *17*(1), 45. <https://doi.org/10.3390/ijerph17010045>

Herráiz, E. D., & Gutiérrez, R. B. (2016). Social Support as a School Victimisation Risk Factor. *Journal of Child and Family Studies*, *25*(12), 3473–3480. <https://doi.org/10.1007/s10826-016-0503-9>

Hokoda, A., Lu, H.-H. A., & Angeles, M. (2006). School Bullying in Taiwanese Adolescents. *Journal of Emotional Abuse*, *6*(4), 69–90. <https://doi.org/10.1300/J135v06n04_04>

Holfeld, B., & Baitz, R. (2020). The Mediating and Moderating Effects of Social Support and School Climate on the Association between Cyber Victimization and Internalizing Symptoms. *Journal of Youth and Adolescence*, *49*(11), 2214–2228. <https://doi.org/10.1007/s10964-020-01292-0>

Hong, J. S., Gómez, A., Kim, J., & Peguero, A. A. (2023). Easy Talking With Parents as a Buffer in the Association Between Bullying Victimization and Declining Academic Performance among Foreign-Born and U.S.-Born Adolescents. *Applied Research in Quality of Life*, *18*(3), 1453–1468. <https://doi.org/10.1007/s11482-023-10146-3>

Hong, J. S., Kim, D. H., Burlaka, V., Peguero, A. A., Padilla, Y. C., & Espelage, D. L. (2021b). Bullying Victimization and Internalizing Problems of Foreign-Born and U.S.-Born Latino/Hispanic and Asian Adolescents in the United States: The Moderating Role of Parental Monitoring. *Journal of the Society for Social Work and Research*, *12*(3), 445–464. <https://doi.org/10.1086/715891>

Hong, J. S., Kim, D. H., deLara, E. W., Wei, H.-S., Prisner, A., & Alexander, N. B. (2021a). Parenting Style and Bullying and Victimization: Comparing Foreign-Born Asian, U.S.-Born Asian, and White American Adolescents. *Journal of Family Violence*, *36*(7), 799–811. <https://doi.org/10.1007/s10896-020-00176-y>

Hong, J. S., Ryou, B., & Piquero, A. R. (2020). Do Family-Level Factors Associated With Bullying Perpetration and Peer Victimization Differ by Race? Comparing European American and African American Youth. *Journal of Interpersonal Violence*, *35*(21–22), 4327–4349. <https://doi.org/10.1177/0886260517714441>

Hsieh, Y.-P. (2020). Parental psychological control and adolescent cyberbullying victimisation and perpetration: The mediating roles of avoidance motivation and revenge motivation. *Asia Pacific Journal of Social Work and Development*, *30*(3), 212–226. <https://doi.org/10.1080/02185385.2020.1776153>

Ilevbare, F., Fagbenro, D., & Idemudia, E. (2023). Personality Traits, Gender, Parenting Styles and Cyberbullying: A Quantitative Study of In-School Adolescents in Nigeria. *Journal of Infant, Child, and Adolescent Psychotherapy*, *22*(2), 154–168. <https://doi.org/10.1080/15289168.2023.2203600>

Ioannidou, L., & Zafiropoulou, M. (2021). Parenting Practices, Victimization, and Negative Affectivity in Child Internalizing Symptoms: Moderated-Mediation Models. *International Journal of Developmental Science*, *15*(1–2), 19–28. <https://doi.org/10.3233/DEV-210308>

Jiang, S., Jiang, C., Ren, Q., & Wang, L. (2021). Cyber victimization and psychological well-being among Chinese adolescents: Mediating role of basic psychological needs satisfaction and moderating role of positive parenting. *Children and Youth Services Review*, *130*, 106248. <https://doi.org/10.1016/j.childyouth.2021.106248>

Jutengren, G., Kerr, M., & Stattin, H. (2011). Adolescents’ deliberate self-harm, interpersonal stress, and the moderating effects of self-regulation: A two-wave longitudinal analysis. *Journal of School Psychology*, *49*(2), 249–264. <https://doi.org/10.1016/j.jsp.2010.11.001>

Katz, I., Lemish, D., Cohen, R., & Arden, A. (2019). When parents are inconsistent: Parenting style and adolescents’ involvement in cyberbullying. *Journal of Adolescence*, *74*(1), 1–12. <https://doi.org/10.1016/j.adolescence.2019.04.006>

Kelly, A. B., Garnett, M. S., Attwood, T., & Peterson, C. (2008). Autism Spectrum Symptomatology in Children: The Impact of Family and Peer Relationships. *Journal of Abnormal Child Psychology*, *36*(7), 1069–1081. <https://doi.org/10.1007/s10802-008-9234-8>

Kim, J., & Kim, E. (2019). Bullied by Siblings and Peers: The Role of Rejecting/Neglecting Parenting and Friendship Quality Among Korean Children. *Journal of Interpersonal Violence*, *34*(11), 2203–2226. <https://doi.org/10.1177/0886260516659659>

Klomek, A. B., Kopelman-Rubin, D., Al-Yagon, M., Berkowitz, R., Apter, A., & Mikulincer, M. (2016). Victimization by Bullying and Attachment to Parents and Teachers Among Student Who Report Learning Disorders and/or Attention Deficit Hyperactivity Disorder. *Learning Disability Quarterly*, *39*(3), 182–190. <https://doi.org/10.1177/0731948715616377>

Kokkinos, C. M. (2013). Bullying and Victimization in Early Adolescence: Associations With Attachment Style and Perceived Parenting. *Journal of School Violence*, *12*(2), 174–192. <https://doi.org/10.1080/15388220.2013.766134>

Kokkinos, C. M., & Panayiotou, G. (2007). Parental discipline practices and locus of control: Relationship to bullying and victimization experiences of elementary school students. *Social Psychology of Education*, *10*(3), 281–301. <https://doi.org/10.1007/s11218-007-9021-3>

Kokkinos, C. M., Antoniadou, N., Asdre, A., & Voulgaridou, K. (2016). Parenting and Internet Behavior Predictors of Cyber-Bullying and Cyber-Victimization among Preadolescents. *Deviant Behavior*, *37*(4), 439–455. <https://doi.org/10.1080/01639625.2015.1060087>

Košir, K., Zakšek, M., & Kozina, A. (2023). School Belongingness and Family Support as Predictors of School Bullying Perpetration and Victimization in Adolescents: Are Relations the Same for Students with an Immigrant Background? *Victims & Offenders*, 1–19. <https://doi.org/10.1080/15564886.2023.2181251>

Krisnana, I., Rachmawati, P. D., Arief, Y. S., Kurnia, I. D., Nastiti, A. A., Safitri, I. F. N., & Putri, A. T. K. (2021). Adolescent characteristics and parenting style as the determinant factors of bullying in Indonesia: A cross-sectional study. *International Journal of Adolescent Medicine and Health*, *33*(5), 20190019. <https://doi.org/10.1515/ijamh-2019-0019>

Lardier, D. T., Barrios, V. R., Garcia-Reid, P., & Reid, R. J. (2016). Suicidal ideation among suburban adolescents: The influence of school bullying and other mediating risk factors. *Journal of Child & Adolescent Mental Health*, *28*(3), 213–231. <https://doi.org/10.2989/17280583.2016.1262381>

Larrañaga, E., Yubero, S., Ovejero, A., & Navarro, R. (2016). Loneliness, parent-child communication and cyberbullying victimization among Spanish youths. *Computers in Human Behavior*, *65*, 1–8. <https://doi.org/10.1016/j.chb.2016.08.015>

Larsen, J. K., Vermulst, A. A., Eisinga, R., English, T., Gross, J. J., Hofman, E., Scholte, R. H. J., & Engels, R. C. M. E. (2012). Social Coping by Masking? Parental Support and Peer Victimization as Mediators of the Relationship Between Depressive Symptoms and Expressive Suppression in Adolescents. *Journal of Youth and Adolescence*, *41*(12), 1628–1642. <https://doi.org/10.1007/s10964-012-9782-7>

Li, R., Yao, M., Liu, H., & Chen, Y. (2019). Relationships among autonomy support, psychological control, coping, and loneliness: Comparing victims with nonvictims. *Personality and Individual Differences*, *138*, 266–272. <https://doi.org/10.1016/j.paid.2018.10.001>

Low, S., & Espelage, D. (2014). Conduits from community violence exposure to peer aggression and victimization: Contributions of parental monitoring, impulsivity, and deviancy. *Journal of Counseling Psychology*, *61*(2), 221–231. <https://doi.org/10.1037/a0035207>

Malecki, C. K., Demaray, M. K., & Davidson, L. M. (2008). The Relationship Among Social Support, Victimization, and Student Adjustment in a Predominantly Latino Sample. *Journal of School Violence*, *7*(4), 48–71. <https://doi.org/10.1080/15388220801973847>

Malm, E. K., & Henrich, C. C. (2019). Longitudinal Relationships Between Parent Factors, Children’s Bullying, and Victimization Behaviors. *Child Psychiatry & Human Development*, *50*(5), 789–802. <https://doi.org/10.1007/s10578-019-00882-9>

Malm, E. K., Henrich, C., Varjas, K., & Meyers, J. (2017). Parental Self-Efficacy and Bullying in Elementary School. *Journal of School Violence*, *16*(4), 411–425. <https://doi.org/10.1080/15388220.2016.1168743>

Marraccini, M. E., Resnikoff, A. W., Brick, L. A., Brier, Z. M. F., & Nugent, N. R. (2022). Adolescent perceptions of school before and after psychiatric hospitalization: Predicting suicidal ideation. *School Psychology*, *37*(2), 119–132. <https://doi.org/10.1037/spq0000487>

Marret, M. J., & Choo, W. Y. (2017). Factors associated with online victimisation among Malaysian adolescents who use social networking sites: A cross-sectional study. *BMJ Open*, *7*(6), e014959. <https://doi.org/10.1136/bmjopen-2016-014959>

Méndez, I., Ruiz-Esteban, C., & López-García, J. J. (2017). Risk and Protective Factors Associated to Peer School Victimization. *Frontiers in Psychology*, *8*. <https://doi.org/10.3389/fpsyg.2017.00441>

Nansel, T. R., Overpeck, M., Pilla, R. S., Ruan, W. J., Simons-Morton, B., & Scheidt, P. (2001). Bullying Behaviors Among US Youth: Prevalence and Association With Psychosocial Adjustment. *JAMA*, *285*(16), 2094. <https://doi.org/10.1001/jama.285.16.2094>

Navarro, R., Serna, C., Martínez, V., & Ruiz-Oliva, R. (2013). The role of Internet use and parental mediation on cyberbullying victimization among Spanish children from rural public schools. *European Journal of Psychology of Education*, *28*(3), 725–745. <https://doi.org/10.1007/s10212-012-0137-2>

Nozaki, Y. (2019). Why do bullies matter?: The impacts of bullying involvement on Adolescents’ life satisfaction via an adaptive approach. *Children and Youth Services Review*, *107*, 104486. <https://doi.org/10.1016/j.childyouth.2019.104486>

Nuñez-Fadda, S. M., Castro-Castañeda, R., Vargas-Jiménez, E., Musitu-Ochoa, G., & Callejas-Jerónimo, J. E. (2020). Bullying Victimization among Mexican Adolescents: Psychosocial Differences from an Ecological Approach. *International Journal of Environmental Research and Public Health*, *17*(13), 4831. <https://doi.org/10.3390/ijerph17134831>

Nuñez-Fadda, S. M., Castro-Castañeda, R., Vargas-Jiménez, E., Musitu-Ochoa, G., & Callejas-Jerónimo, J. E. (2022). Impact of Bullying—Victimization and Gender over Psychological Distress, Suicidal Ideation, and Family Functioning of Mexican Adolescents. *Children*, *9*(5), 747. <https://doi.org/10.3390/children9050747>

Olenik-Shemesh, D., & Heiman, T. (2017). Cyberbullying Victimization in Adolescents as Related to Body Esteem, Social Support, and Social Self-Efficacy. *The Journal of Genetic Psychology*, *178*(1), 28–43. <https://doi.org/10.1080/00221325.2016.1195331>

Ortega Barón, J., Postigo, J., Iranzo, B., Buelga, S., & Carrascosa, L. (2018). Parental Communication and Feelings of Affiliation in Adolescent Aggressors and Victims of Cyberbullying. *Social Sciences*, *8*(1), 3. <https://doi.org/10.3390/socsci8010003>

Owusu, D. N., Owusu Ansah, K., Dey, N. E. Y., Duah, H. O., & Agbadi, P. (2022). Bullying and truancy amongst school-going adolescents in Timor-Leste: Results from the 2015 global school-based health survey. *Heliyon*, *8*(1), e08797. <https://doi.org/10.1016/j.heliyon.2022.e08797>

Özdemir, Y. (2014). Cyber victimization and adolescent self-esteem: The role of communication with parents: Cyber victimization and self-esteem. *Asian Journal of Social Psychology*, *17*(4), 255–263. <https://doi.org/10.1111/ajsp.12070>

Papadaki, E., & Giovazolias, T. (2015). The Protective Role of Father Acceptance in the Relationship Between Maternal Rejection and Bullying: A Moderated-mediation Model. *Journal of Child and Family Studies*, *24*(2), 330–340. <https://doi.org/10.1007/s10826-013-9839-6>

Perasso, G., Carone, N., Lombardy Group 2014, H. B. in S. A. C., & Barone, L. (2021). Written and visual cyberbullying victimization in adolescence: Shared and unique associated factors. *European Journal of Developmental Psychology*, *18*(5), 658–677. <https://doi.org/10.1080/17405629.2020.1810661>

Poteat, V. P., Mereish, E. H., DiGiovanni, C. D., & Koenig, B. W. (2011). The effects of general and homophobic victimization on adolescents’ psychosocial and educational concerns: The importance of intersecting identities and parent support. *Journal of Counseling Psychology*, *58*(4), 597–609. <https://doi.org/10.1037/a0025095>

Ren, P., Yang, L., Chen, C., & Luo, F. (2023). Parental control and adolescents’ bullying victimization: The moderating role of teacher support. *Current Psychology*, *42*(32), 27952–27964. <https://doi.org/10.1007/s12144-022-03864-8>

Rinaldi, C. M., Bulut, O., Muth, T., & Di Stasio, M. (2023). The Influence of Parenting Dimensions and Junior High School Students’ Involvement in Bullying. *Journal of School Violence*, *22*(2), 183–197. <https://doi.org/10.1080/15388220.2022.2162534>

*Rose, C. A., Espelage, D. L., Monda-Amaya, L. E., Shogren, K. A., & Aragon, S. R. (2015). Bullying and Middle School Students With and Without Specific Learning Disabilities: An Examination of Social-Ecological Predictors. Journal of Learning Disabilities, 48(3), 239–254.* [*https://doi.org/10.1177/0022219413496279*](https://doi.org/10.1177/0022219413496279)

Rothon, C., Head, J., Klineberg, E., & Stansfeld, S. (2011). Can social support protect bullied adolescents from adverse outcomes? A prospective study on the effects of bullying on the educational achievement and mental health of adolescents at secondary schools in East London. *Journal of Adolescence*, *34*(3), 579–588. <https://doi.org/10.1016/j.adolescence.2010.02.007>

Russo, J. N., Griese, E. R., & Bares, V. J. (2018). Examining prevalence and impact of peer victimization and social support for rural youth. *South Dakota medicine: the journal of the South Dakota State Medical Association*, *71*(10), 448.

Sarhangi, N., Rostami, M., Abbasirad, R., Fasihi, M., & Ahmadboukani, S. (2023). Cyber victimization and suicidal behavior in high school students: The mediating role of psychological problems and perceived social support. *Psychology in the Schools*, *60*(7), 2395–2408. <https://doi.org/10.1002/pits.22868>

Sasson, H., & Mesch, G. (2017). The Role of Parental Mediation and Peer Norms on the Likelihood of Cyberbullying. *The Journal of Genetic Psychology*, *178*(1), 15–27. <https://doi.org/10.1080/00221325.2016.1195330>

Sener, O. (2021). Investigation of the relationship between family relations and peer bullying of primary and secondary school students. *Ilkogretim Online*, *20*(1).

Serra-Negra, J. M., Paiva, S. M., Bendo, C. B., Fulgêncio, L. B., Lage, C. F., Corrêa-Faria, P., & Pordeus, I. A. (2015). Verbal school bullying and life satisfaction among Brazilian adolescents: Profiles of the aggressor and the victim. *Comprehensive Psychiatry*, *57*, 132–139. <https://doi.org/10.1016/j.comppsych.2014.11.004>

Shin, J.-H., Hong, J. S., Yoon, J., & Espelage, D. L. (2014). Interparental Conflict, Parenting Behavior, and Children’s Friendship Quality as Correlates of Peer Aggression and Peer Victimization Among Aggressor/Victim Subgroups in South Korea. *Journal of Interpersonal Violence*, *29*(10), 1933–1952. <https://doi.org/10.1177/0886260513511695>

Stavrinides, P., Nikiforou, M., & Georgiou, S. (2015). Do mothers know? Longitudinal associations between parental knowledge, bullying, and victimization. *Journal of Social and Personal Relationships*, *32*(2), 180–196. <https://doi.org/10.1177/0265407514525889>

Strohmeier, D., Gradinger, P., & Yanagida, T. (2022). The Role of Intrapersonal-, Interpersonal-, Family-, and School-Level Variables in Predicting Bias-Based Cybervictimization. *The Journal of Early Adolescence*, *42*(9), 1175–1203. <https://doi.org/10.1177/02724316211010335>

Tang, W., Chen, M., Wang, N., Deng, R., Tang, H., Xu, W., & Xu, J. (2023). Bullying victimization and internalizing and externalizing problems in school-aged children: The mediating role of sleep disturbance and the moderating role of parental attachment. *Child Abuse & Neglect*, *138*, 106064. <https://doi.org/10.1016/j.chiabu.2023.106064>

Tao, S., Reichert, F., Law, N., & Rao, N. (2022). Digital Technology Use and Cyberbullying Among Primary School Children: Digital Literacy and Parental Mediation as Moderators. *Cyberpsychology, Behavior, and Social Networking*, *25*(9), 571–579. <https://doi.org/10.1089/cyber.2022.0012>

Tian, L., Huang, J., & Huebner, E. S. (2023). Profiles and Transitions of Cyberbullying Perpetration and Victimization from Childhood to Early Adolescence: Multi-Contextual Risk and Protective Factors. *Journal of Youth and Adolescence*, *52*(2), 434–448. <https://doi.org/10.1007/s10964-022-01633-1>

Tobias, S., & Chapanar, T. (2016). Predicting resilience after cyberbully victimization among high school students. *Journal of Psychological and Educational Research*, *24*(1), 7.

van Hoof, A., Raaijmakers, Q. A. W., van Beek, Y., Hale, W. W., & Aleva, L. (2008). A Multi-mediation Model on the Relations of Bullying, Victimization, Identity, and Family with Adolescent Depressive Symptoms. *Journal of Youth and Adolescence*, *37*(7), 772–782. <https://doi.org/10.1007/s10964-007-9261-8>

Vannucci, A., Fagle, T. R., Simpson, E. G., & Ohannessian, C. M. (2021). Perceived Family and Friend Support Moderate Pathways From Peer Victimization to Substance Use in Early-Adolescent Girls and Boys: A Moderated-Mediation Analysis. *The Journal of Early Adolescence*, *41*(1), 128–166. <https://doi.org/10.1177/0272431620931187>

Vargas, A. E. O., & Monjardín, M. del R. M. (2019). Impacto multifacético del ambiente familiar en situaciones de violencia escolar en hombres y mujeres. *Psicologia Escolar e Educacional*, *23*, e192847. <https://doi.org/10.1590/2175-35392019012847>

Varsamis, P., Halios, H., Katsanis, G., & Papadopoulos, A. (2022). The role of basic psychological needs in bullying victimisation in the family and at school. *Journal of Psychologists and Counsellors in Schools*, *32*(2), 230–242. <https://doi.org/10.1017/jgc.2021.9>

Veenstra, R., Lindenberg, S., Oldehinkel, A. J., De Winter, A. F., Verhulst, F. C., & Ormel, J. (2005). Bullying and Victimization in Elementary Schools: A Comparison of Bullies, Victims, Bully/Victims, and Uninvolved Preadolescents.*Developmental Psychology*, *41*(4), 672–682. <https://doi.org/10.1037/0012-1649.41.4.672>

Verešová, M., & Mujkošová, K. (2020). Relationship Between Risk Behavior Of Pubescents And Characteristics Of Their Family Environment. *Ad Alta: Journal of Interdisciplinary Research*, *10*(2).

Wachs, S., Görzig, A., Wright, M. F., Schubarth, W., & Bilz, L. (2020). Associations among Adolescents’ Relationships with Parents, Peers, and Teachers, Self-Efficacy, and Willingness to Intervene in Bullying: A Social Cognitive Approach. *International Journal of Environmental Research and Public Health*, *17*(2), 420. <https://doi.org/10.3390/ijerph17020420>

Wang, C., La Salle, T. P., Do, K. A., Wu, C., & Sullivan, K. E. (2019). Does parental involvement matter for students' mental health in middle school?.*School psychology (Washington, D.C.), 34(2), 222–232.* [*https://doi.org/10.1037/spq0000300*](https://doi.org/10.1037/spq0000300)

Wang, C., La Salle, T., Wu, C., & Liu, J. L. (2022). Do Parental Involvement and Adult Support Matter for Students’ Suicidal Thoughts and Behavior in High School? *School Psychology Review*, *51*(3), 329–342. <https://doi.org/10.1080/2372966X.2021.1873058>

Wang, J., Iannotti, R. J., & Nansel, T. R. (2009). School Bullying Among Adolescents in the United States: Physical, Verbal, Relational, and Cyber. *Journal of Adolescent Health*, *45*(4), 368–375. <https://doi.org/10.1016/j.jadohealth.2009.03.021>

Wang, Y.-J., & Chen, I.-H. (2023). A Multilevel Analysis of Factors Influencing School Bullying in 15-Year-Old Students. *Children*, *10*(4), 653. <https://doi.org/10.3390/children10040653>

Wong, N., & McBride, C. (2016). To Use Facebook for Good: Usage, Cyberbullying Involvement, and Perceived Social Support. *Child Studies in Asia-Pacific Contexts*, *6*(2), 59–72. <https://doi.org/10.5723/csac.2016.6.2.059>

Wong, T. K. Y., & Konishi, C. (2021). The interplay of perceived parenting practices and bullying victimization among Hong Kong adolescents. *Journal of Social and Personal Relationships*, *38*(2), 668–689. <https://doi.org/10.1177/0265407520969907>

Wright, M. F. (2016b). Cybervictimization and Substance Use Among Adolescents: The Moderation of Perceived Social Support. *Journal of Social Work Practice in the Addictions*, *16*(1–2), 93–112. <https://doi.org/10.1080/1533256X.2016.1143371>

Wright, M. F. (2016a). Bullying among adolescents in residential programs and in public school: The role of individual and contextual predictors. *Journal of Aggression, Conflict and Peace Research*, *8*(2), 86–98. <https://doi.org/10.1108/JACPR-10-2015-0192>

Wright, M. F. (2017). Cyber Victimization and Depression Among Adolescents With Intellectual Disabilities and Developmental Disorders: The Moderation of Perceived Social Support. *Journal of Mental Health Research in Intellectual Disabilities*, *10*(2), 126–143. <https://doi.org/10.1080/19315864.2016.1271486>

Wright, M. F. (2018). Cyber Victimization and Depression among Adolescents with Autism Spectrum Disorder: The Buffering Effects of Parental Mediation and Social Support. *Journal of Child & Adolescent Trauma*, *11*(1), 17–25. <https://doi.org/10.1007/s40653-017-0169-5>

Wright, M. F., & Wachs, S. (2020). Parental Support, Health, and Cyberbullying among Adolescents with Intellectual and Developmental Disabilities. *Journal of Child and Family Studies*, *29*(9), 2390–2401. <https://doi.org/10.1007/s10826-020-01739-9>

Wright, M. F., Hong, J. S., & Wachs, S. (2021). The Relationship between parenting styles and relationally victimized adolescents’ adjustment: Moderation of parents’ victimization status during adolescence. *Journal of Family Trauma, Child Custody & Child Development*, *18*(2), 93–107. <https://doi.org/10.1080/26904586.2021.1918036>

Wu, W., Ding, W., Xie, R., Tan, D., Wang, D., Sun, B., & Li, A. W. (2022a). Bidirectional Longitudinal Relationships between Maternal Psychological Control and Bullying/Victimization among Father-Absent Left-Behind Children in China. *Journal of Interpersonal Violence*, *37*(17–18), NP15925–NP15943. <https://doi.org/10.1177/08862605211022062>

Wu, W., Guo, Z., Li, S., Tu, F., Wu, X., Ma, X., Teng, Z., Chen, Y., & Zeng, Y. (2022b). The influence of parental autonomy support on cyberbullying victimization of high school students: A latent moderation analysis. *Acta Psychologica*, *230*, 103739. <https://doi.org/10.1016/j.actpsy.2022.103739>

Wu, W., Xie, R., Ding, W., Jiang, M., Sun, Z., Kayani, S., & Li, W. (2023b). Can maternal involvement protect children from bullying victimization when fathers migrate for work? A serial mediation model. *Journal of Child and Family Studies*, *32*(10), 3148–3158. <https://doi.org/10.1007/s10826-023-02629-6>

Wu, X., Zhen, R., Shen, L., Tan, R., & Zhou, X. (2023a). Patterns of Elementary School Students’ Bullying Victimization: Roles of Family and Individual Factors. *Journal of Interpersonal Violence*, *38*(3–4), 2410–2431. <https://doi.org/10.1177/08862605221101190>

Xiao, Y., Ran, H., Che, Y., Fang, D., Wang, S., Chen, L., Liang, X., Peng, J., Sun, H., Li, Q., Shi, Y., & Lu, J. (2023). Childhood maltreatment and parenting style associated school bullying in Chinese children and adolescents: An analytical epidemiology evidence. *Journal of Affective Disorders*, *331*, 386–392. <https://doi.org/10.1016/j.jad.2023.02.153>

Yabko, B. A., Hokoda, A., & Ulloa, E. C. (2008). Depression as a Mediator Between Family Factors and Peer-Bullying Victimization in Latino Adolescents. *Violence and Victims*, *23*(6), 727–742. <https://doi.org/10.1891/0886-6708.23.6.727>

Ye, Y., Wang, C., Zhu, Q., He, M., Havawala, M., Bai, X., & Wang, T. (2022). Parenting and Teacher–Student Relationship as Protective Factors for Chinese Adolescent Adjustment During COVID-19. *School Psychology Review*, *51*(2), 187–205. <https://doi.org/10.1080/2372966X.2021.1897478>

Yu, L., Li, X., Hu, Q., Guo, Z., Hong, D., Huang, Y., Xu, Y., Zhang, J., Xu, Q., & Jiang, S. (2023). School bullying and its risk and protective factors in Chinese early adolescents: A latent transition analysis. *Aggressive Behavior*, *49*(4), 345–358. <https://doi.org/10.1002/ab.22080>

Zhang, H., Han, T., Ma, S., Qu, G., Zhao, T., Ding, X., Sun, L., Qin, Q., Chen, M., & Sun, Y. (2022a). Association of child maltreatment and bullying victimization among Chinese adolescents: The mediating role of family function, resilience, and anxiety. *Journal of Affective Disorders*, *299*, 12–21. <https://doi.org/10.1016/j.jad.2021.11.053>

Zhang, J., Lin, G., Cai, Q., Hu, Q., Xu, Y., Guo, Z., Hong, D., Huang, Y., Lv, Y., Chen, J., & Jiang, S. (2022b). The role of family and peer factors in the development of early adolescent depressive symptoms: A latent class growth analysis. *Frontiers in Psychiatry*, *13*, 914055. <https://doi.org/10.3389/fpsyt.2022.914055>

Zhang, Y., Li, H., Chen, G., Li, B., Li, N., & Zhou, X. (2023). The moderating roles of resilience and social support in the relationships between bullying victimization and well‐being among Chinese adolescents: Evidence from PISA 2018. *British Journal of Psychology*, bjop.12678. <https://doi.org/10.1111/bjop.12678>

Zhou, H., Wang, Q., Yu, S., & Zheng, Q. (2022a). Negative Parenting Style and Perceived Non-Physical Bullying at School: The Mediating Role of Negative Affect Experiences and Coping Styles. *International Journal of Environmental Research and Public Health*, *19*(10), 6206. <https://doi.org/10.3390/ijerph19106206>

Zhou, J., Li, X., Zou, Y., & Gong, X. (2022b). Longitudinal relations among family dysfunction, depressive symptoms, and cyberbullying involvement in Chinese early adolescents: Disentangling between- and within-person associations. *Development and Psychopathology*, 1–9. <https://doi.org/10.1017/S0954579422001274>

Zhou, L., & Li, C. (2021). Factors associated with cyberbullying among vocational students based on the ecological system model in an ethnic minority area. *Medicine*, *100*(40), e27226. <https://doi.org/10.1097/MD.0000000000027226>

Zhou, P., Dong, J., Liu, J., Zhang, Y., Ren, P., Xin, T., & Wang, Z. (2023). Relationship between Parent-Child Relationships and Peer Victimization: A Moderated Mediation Model of Self-esteem and Resilience. *Journal of Child and Family Studies*, *32*(3), 641–651. <https://doi.org/10.1007/s10826-022-02457-0>

**Appendix C - Table A.1.** Risk of bias - Methodological quality for each study (N=158)

| **References** | **Q1** | **Q2** | **Q3** | **Q4** | **Q5** | **Q6** | **Q7** | **Q8** | **Q9** | **Q10** | **Q11** | **Q12** | **Q13** | **Q14** | **Total** | **Total** |
| --- | --- | --- | --- | --- | --- | --- | --- | --- | --- | --- | --- | --- | --- | --- | --- | --- |
| Affrunti et al., 2014 | YES | YES | NR | NO | NO | NO | NO | YES | YES | NO | YES | NR | NA | NO | 38,46 | POOR |
| Akkurt Nurtan et al., 2022 | YES | YES | NR | NO | YES | NO | NO | YES | YES | NO | YES | NR | NA | NO | 46,15 | POOR |
| Alcantara et al, 2017 | YES | YES | YES | YES | NO | NO | NO | YES | YES | NO | YES | NR | NA | NO | 53,84 | FAIR |
| Alikasifoglu et al., 2007 | YES | YES | YES | NO | NO | NO | NO | NO | YES | NO | YES | NR | NA | NO | 38,46 | POOR |
| Arabiat et al., 2018 | YES | YES | YES | YES | NO | NO | NO | NO | YES | NO | YES | NR | NA | NO | 46,15 | POOR |
| Arango et al., 2019 | YES | YES | NR | YES | NO | YES | YES | YES | YES | YES | YES | NR | YES | NO | 71,42 | FAIR |
| Balan et al., 2018 | YES | YES | NR | NO | NO | NO | NO | YES | YES | NO | YES | NR | NA | YES | 46,15 | POOR |
| Balan et al., 2022 | YES | YES | NR | NO | NO | NO | NO | YES | YES | NO | YES | NR | NA | NO | 38,46 | POOR |
| Bartolomé Gutiérrez & Díaz Herráiz, 2019 | YES | YES | NR | YES | YES | NO | NO | YES | YES | NO | YES | NR | NA | NO | 53,84 | FAIR |
| Beran & Violato, 2004 | YES | YES | NR | YES | NO | NO | NO | YES | YES | NO | NO | NR | NA | YES | 46,15 | POOR |
| Bjereld et al., 2023 | YES | YES | YES | CD | NO | NO | NO | NO | YES | NO | YES | NR | NA | YES | 46,15 | POOR |
| Boniel-Nissim & Sasson, 2018 | YES | YES | YES | NO | NO | NO | NO | YES | YES | NO | CD | NR | NA | NO | 46,15 | POOR |
| Bordin & Handegård, 2023 | YES | YES | YES | YES | NO | NO | NO | YES | YES | NO | NO | NR | NA | NO | 46,15 | POOR |
| Carter et al., 2020 | YES | YES | NR | NO | NO | NO | NO | YES | YES | NO | YES | NR | NA | YES | 46,15 | POOR |
| Carter et al., 2023 | YES | YES | NR | NO | NO | NO | NO | YES | YES | NO | YES | NR | NA | YES | 46,15 | POOR |
| Cassiani-Miranda et al., 2022 | YES | YES | NR | NO | YES | NO | NO | YES | YES | NO | YES | NR | NA | YES | 53,84 | FAIR |
| Cassidy, 2009 | YES | YES | NR | NO | NO | NO | NO | YES | YES | NO | NO | NR | NA | YES | 38,46 | POOR |
| Cerezo et al., 2018 | YES | YES | NR | NO | NO | NO | NO | YES | YES | NO | YES | NR | NA | YES | 46,15 | POOR |
| Chang et al., 2015 | YES | YES | YES | NO | NO | NO | NO | YES | YES | NO | YES | NR | NA | NO | 46,15 | POOR |
| Charalampous et al., 2018 | YES | YES | YES | NO | NO | YES | YES | YES | YES | NO | YES | NR | YES | YES | 71,42 | FAIR |
| Charalampous et al., 2019 | YES | YES | NR | NO | NO | NO | NO | YES | YES | NO | YES | NR | NA | YES | 46,15 | POOR |
| Chen et al., 2021 | YES | YES | NR | YES | YES | NO | NO | NO | YES | NO | YES | NR | NA | YES | 53,84 | FAIR |
| Chen et al., 2022 | YES | YES | YES | YES | NO | NO | NO | YES | YES | NO | YES | NR | NA | YES | 61,53 | FAIR |
| Chen, 2020 | YES | YES | NR | NO | NO | NO | NO | YES | YES | NO | YES | NR | NA | NO | 38,46 | POOR |
| Chicoine et al., 2021 | YES | YES | NR | YES | NO | YES | YES | YES | YES | YES | YES | NR | YES | NO | 76,92 | GOOD |
| Cho & Lee, 2018 | YES | YES | YES | NO | NO | NO | NO | YES | YES | NO | YES | NR | NA | YES | 53,84 | FAIR |
| Cho & Norman, 2019 | YES | YES | NR | NO | NO | NO | NO | YES | YES | NO | YES | NR | NA | NO | 38,46 | POOR |
| Cho et al., 2019 | YES | YES | YES | NO | NO | NO | NO | YES | YES | NO | YES | NR | NA | NO | 46,15 | POOR |
| Choi & Park, 2018 | YES | YES | NR | NO | NO | YES | YES | NO | YES | NO | YES | NR | NA | NO | 46,15 | POOR |
| Choi, 2023 | YES | YES | NO | NO | NO | NO | NO | YES | YES | NO | NO | NR | NA | NO | 30,76 | POOR |
| Chui et al., 2022 | YES | YES | NR | NO | NO | NO | NO | YES | YES | NO | YES | NR | NA | NO | 38,46 | POOR |
| Davidson & Demaray, 2007 | YES | YES | YES | NO | NO | NO | NO | YES | YES | NO | YES | NR | NA | NO | 46,15 | POOR |
| DeSmet et al., 2021 | YES | YES | YES | NO | NO | NO | NO | YES | YES | NO | NO | NR | NA | NO | 38,46 | POOR |
| Doty et al., 2018 | YES | YES | YES | YES | NO | NO | NO | NO | YES | NO | CD | NR | NA | YES | 46,15 | POOR |
| Dudley et al., 2023 | YES | YES | YES | YES | NO | NO | NO | YES | YES | NO | YES | NR | NA | YES | 61,53 | FAIR |
| Duggins et al., 2016 | YES | YES | NR | NO | NO | YES | YES | YES | YES | NO | NO | NR | NO | NO | 46,15 | POOR |
| Duong et al., 2009 | YES | YES | YES | NO | NO | NO | NO | YES | YES | NO | YES | NR | NA | YES | 53,84 | FAIR |
| Elledge et al., 2019 | YES | YES | YES | NO | NO | NO | NO | YES | YES | NO | YES | NR | NA | NO | 46,15 | POOR |
| Escario et al., 2023 | YES | YES | NR | NO | NO | NO | NO | NO | YES | NO | NO | NR | NA | YES | 30,76 | POOR |
| Espino et al., 2023 | YES | YES | NR | NO | YES | NO | NO | YES | YES | NO | YES | NR | NA | NO | 46,15 | POOR |
| Fanti et al., 2012 | YES | YES | NR | NO | NO | YES | YES | NO | YES | NO | YES | NR | YES | YES | 57,14 | FAIR |
| Fox et al., 2022 | YES | YES | NR | NO | NO | YES | YES | NO | YES | NO | YES | NR | NO | NO | 46,15 | POOR |
| Fredrick et al., 2022 | YES | YES | YES | YES | NO | YES | YES | YES | YES | YES | YES | NR | YES | YES | 84,61 | GOOD |
| Freitas et al., 2022 | YES | YES | YES | YES | NO | NO | NO | NO | YES | NO | YES | NR | NA | NO | 46,15 | POOR |
| Garaigordobil & Machimbarrena, 2017 | YES | YES | NR | NO | NO | NO | NO | YES | YES | NO | YES | NR | NA | YES | 46,15 | POOR |
| Garaigordobil & Navarro, 2022 | YES | YES | NR | YES | YES | NO | NO | YES | YES | NO | YES | NR | NA | YES | 61,53 | FAIR |
| Georgiou & Stavrinides, 2013 | YES | YES | YES | YES | NO | NO | NO | YES | YES | NO | YES | NR | NA | NO | 53,84 | FAIR |
| Georgiou et al., 2013 | YES | YES | YES | YES | NO | NO | NO | NO | YES | NO | YES | NR | NA | YES | 53,84 | FAIR |
| Georgiou et al., 2018 | YES | YES | YES | YES | NO | NO | NO | NO | YES | NO | YES | NR | NA | YES | 53,84 | FAIR |
| Georgiou, 2008 (a) | YES | YES | YES | YES | NO | NO | NO | YES | YES | NO | YES | NR | NA | YES | 61,53 | FAIR |
| Georgiou, 2008 (b) | YES | YES | YES | YES | NO | NO | NO | NO | YES | NO | YES | NR | NA | NO | 46,15 | POOR |
| Gofin & Avitzour, 2012 | YES | YES | YES | NO | YES | NO | NO | NO | YES | NO | YES | NR | NA | NO | 46,15 | POOR |
| Goldberg et al., 2023 | YES | YES | NR | NO | NO | NO | NO | NO | YES | NO | NO | NR | NA | NO | 23,07 | POOR |
| Goodfellow et al., 2023 | YES | YES | NR | NO | NO | NO | NO | YES | YES | NO | YES | NR | NA | NO | 38,46 | POOR |
| Goswami, 2011 | YES | YES | NR | NO | NO | NO | NO | YES | YES | NO | NO | NR | NA | NO | 30,76 | POOR |
| Gullone & Robertson, 2008 | YES | YES | NO | NO | NO | NO | NO | YES | YES | NO | CD | NR | NA | NO | 30,76 | POOR |
| Han et al., 2021 | YES | YES | NR | NO | NO | NO | NO | YES | YES | NO | YES | NR | NA | YES | 46,15 | POOR |
| Havewala & Wang, 2021 | YES | YES | NR | NO | NO | YES | YES | YES | YES | NO | YES | NR | NR | NO | 53,84 | FAIR |
| Healy & Sanders, 2014 | YES | YES | YES | YES | NO | YES | YES | NO | YES | YES | YES | YES | YES | NO | 78,57 | GOOD |
| Healy et al., 2015 (a) | YES | YES | YES | NO | NO | NO | NO | NO | YES | NO | YES | NR | NA | YES | 46,15 | POOR |
| Healy et al., 2015 (b) | YES | YES | NR | NO | NO | NO | NO | YES | YES | NO | YES | NR | NA | YES | 46,15 | POOR |
| Heerde et al., 2019 | YES | YES | YES | NO | NO | YES | YES | YES | YES | YES | YES | NR | YES | NO | 71,42 | FAIR |
| Hellheldt et al., 2019 | YES | YES | YES | NO | NO | NO | NO | NO | YES | NO | YES | NR | NA | NO | 38,46 | POOR |
| Herráiz & Gutiérrez, 2016 | YES | YES | NR | NO | YES | NO | NO | YES | YES | NO | YES | NR | NA | NO | 46,15 | POOR |
| Hokoda et al., 2006 | YES | YES | YES | NO | NO | NO | NO | YES | YES | NO | YES | NR | NA | NO | 46,15 | POOR |
| Holfeld & Baitz, 2020 | YES | YES | YES | NO | NO | NO | NO | YES | YES | NO | YES | NR | NA | NO | 46,15 | POOR |
| Hong et al., 2016 | YES | YES | NR | NO | NO | NO | NO | NO | YES | NO | YES | NR | NA | YES | 38,46 | POOR |
| Hong et al., 2020 | YES | YES | NR | NO | NO | NO | NO | NO | YES | NO | YES | NR | NA | YES | 30,76 | POOR |
| Hong et al., 2021 | YES | YES | NR | NO | NO | NO | NO | NO | YES | NO | YES | NR | NA | YES | 38,46 | POOR |
| Hong et al., 2021 | YES | YES | NR | YES | NO | NO | NO | YES | YES | NO | YES | NR | NA | NO | 46,15 | POOR |
| Hong et al., 2023 | YES | YES | YES | NO | NO | NO | NO | NO | YES | NO | YES | NR | NA | NO | 38,46 | POOR |
| Hsieh, 2020 | YES | YES | YES | NO | NO | NO | NO | YES | YES | NO | YES | NR | NA | YES | 53,84 | FAIR |
| Ilevbare et al., 2023 | YES | YES | NR | NO | NO | NO | NO | NO | YES | NO | YES | NR | NA | NO | 30,76 | POOR |
| Ioannidou & Zafiropoulou, 2021 | YES | YES | NR | NO | NO | NO | NO | NO | YES | NO | YES | NR | NA | YES | 38,46 | POOR |
| Jiang et al., 2021 | YES | YES | YES | NO | NO | NO | NO | YES | YES | NO | YES | NR | NA | NO | 46,15 | POOR |
| Jutengren et al., 2011 | YES | YES | YES | NO | NO | YES | YES | YES | YES | YES | NO | NR | NR | YES | 64,28 | FAIR |
| Katz et al., 2019 | YES | YES | NR | NO | YES | NO | NO | YES | YES | NO | YES | NR | NA | YES | 53,84 | FAIR |
| Kelly et al., 2008 | YES | YES | NO | YES | NO | NO | NO | YES | YES | NO | YES | NR | NA | NO | 46,15 | POOR |
| Kim & Kim, 2019 | YES | YES | YES | YES | NO | NO | NO | YES | YES | NO | YES | NR | NA | YES | 61,53 | FAIR |
| Klomek et al., 2015 | YES | YES | YES | NO | NO | NO | NO | YES | YES | NO | CD | NR | NA | YES | 46,15 | POOR |
| Kokkinos & Panayiotou, 2007 | YES | YES | YES | NO | NO | NO | NO | NO | YES | NO | NO | NR | NA | NO | 30,76 | POOR |
| Kokkinos et al., 2016 | YES | YES | NR | NO | NO | NO | NO | YES | YES | NO | YES | NR | NA | NO | 38,46 | POOR |
| Kokkinos, 2013 | YES | YES | NR | NO | NO | NO | NO | NO | YES | NO | YES | NR | NA | YES | 38,46 | POOR |
| Košir et al., 2023 | YES | YES | YES | NO | NO | NO | NO | NO | YES | NO | YES | NR | NA | YES | 46,15 | POOR |
| Krisnana et al., 2021 | YES | YES | NR | YES | NO | NO | NO | NO | YES | NO | YES | NR | NA | NO | 38,46 | POOR |
| Lardier et al., 2016 | YES | YES | NO | NO | NO | NO | NO | YES | YES | NO | NO | NR | NA | NO | 30,76 | POOR |
| Larranaga et al., 2016 | YES | YES | YES | NO | YES | NO | NO | YES | YES | NO | YES | NR | NA | NO | 53,84 | FAIR |
| Larsen et al., 2012 | YES | YES | YES | NO | NO | YES | YES | YES | YES | YES | YES | NR | NO | NO | 64,28 | FAIR |
| Li et al., 2019 | YES | YES | NR | NO | NO | NO | NO | YES | YES | NO | YES | NR | NA | NO | 38,46 | POOR |
| Low & Espelage, 2014 | YES | YES | YES | NO | NO | YES | YES | NO | YES | YES | YES | NR | NR | NO | 61,53 | FAIR |
| Malecki et al., 2008 | YES | YES | NR | NO | NO | NO | NO | YES | YES | NO | NO | NR | NA | NO | 30,76 | POOR |
| Malm & Henrich, 2019 | YES | YES | NR | YES | NO | YES | YES | YES | YES | YES | NO | NR | NR | YES | 64,28 | FAIR |
| Malm et al., 2017 | YES | YES | YES | NO | NO | NO | NO | YES | YES | NO | YES | NR | NA | NO | 46,15 | POOR |
| Marraccini et al., 2022 | YES | YES | NR | YES | NO | YES | YES | YES | YES | YES | YES | NR | YES | NO | 76,92 | GOOD |
| Marret & Choo, 2017 | YES | YES | YES | NO | YES | NO | NO | YES | YES | NO | YES | NR | NA | YES | 53,84 | FAIR |
| Méndez et al., 2017 | YES | YES | NR | YES | NO | NO | NO | YES | YES | NO | CD | NR | NA | YES | 46,15 | POOR |
| Nansel et al., 2001 | YES | YES | YES | NO | YES | NO | NO | NO | YES | NO | YES | NR | NA | NO | 46,15 | POOR |
| Navarro et al., 2013 | YES | YES | YES | NO | YES | NO | NO | YES | YES | NO | YES | NR | NA | NO | 53,84 | FAIR |
| Nozaki, 2019 | YES | YES | YES | NO | NO | NO | NO | YES | YES | NO | YES | NR | NA | NO | 46,15 | POOR |
| Nuñez-Fadda et al., 2020 | YES | YES | YES | NO | YES | NO | NO | YES | YES | NO | YES | NR | NA | YES | 61,53 | FAIR |
| Nuñez-Fadda et al., 2022 | YES | YES | NR | NO | YES | NO | NO | NO | YES | NO | YES | NR | NA | NO | 38,46 | POOR |
| Olenik-Shemesh & Heiman, 2017 | YES | YES | NR | NO | NO | NO | NO | YES | YES | NO | YES | NR | NA | NO | 38,46 | POOR |
| Ortega Barón et al., 2018 | YES | YES | NR | YES | YES | NO | NO | YES | YES | NO | YES | NR | NA | NO | 53,84 | FAIR |
| Owusu et al., 2022 | YES | YES | YES | NO | NO | NO | NO | NO | YES | NO | YES | NR | NA | NO | 38,46 | POOR |
| Özdemir, 2014 | YES | YES | YES | NO | NO | NO | NO | YES | YES | NO | YES | NR | NA | NO | 46,15 | POOR |
| Papadaki & Giovazolias, 2015 | YES | YES | YES | NO | NO | NO | NO | YES | YES | NO | YES | NR | NA | YES | 53,84 | FAIR |
| Perasso et al., 2021 | YES | YES | NR | NO | NO | NO | NO | NO | YES | NO | YES | NR | NA | YES | 38,46 | POOR |
| Poteat et al., 2011 | YES | YES | YES | YES | NO | NO | NO | YES | YES | NO | YES | NR | NA | YES | 61,53 | FAIR |
| Ren et al., 2023 | YES | YES | NR | NO | NO | YES | YES | YES | YES | NO | YES | NR | YES | YES | 61,53 | FAIR |
| Rinaldi et al., 2023 | YES | YES | NO | NO | NO | NO | NO | NO | YES | NO | YES | NR | NA | YES | 38,46 | POOR |
| Rose et al., 2015 | YES | YES | YES | NO | NO | NO | NO | YES | YES | NO | YES | NR | NA | NO | 46,15 | POOR |
| Rothon et al., 2011 | YES | YES | YES | NO | NO | NO | NO | YES | YES | NO | NO | NR | NA | NO | 38,46 | POOR |
| Russo et al., 2021 | YES | YES | YES | NO | NO | NO | NO | YES | YES | NO | YES | NR | NA | NO | 46,15 | POOR |
| Sarhangi et al., 2023 | YES | YES | NR | NO | YES | NO | NO | YES | YES | NO | YES | NR | NA | NO | 46,15 | POOR |
| Sasson & Mesch, 2017 | YES | YES | NR | NO | NO | NO | NO | NO | YES | NO | YES | NR | NA | YES | 38,46 | POOR |
| Sener, 2021 | YES | YES | NR | NO | NO | NO | NO | YES | YES | NO | YES | NR | NA | YES | 46,15 | POOR |
| Serra-Negra et al., 2015 | YES | YES | YES | YES | YES | NO | NO | YES | YES | NO | YES | NR | NA | NO | 61,53 | FAIR |
| Shin et al., 2014 | YES | YES | NR | NO | NO | NO | NO | YES | YES | NO | NO | NR | NA | NO | 30,76 | POOR |
| Stavrinides et al., 2015 | YES | YES | NR | NO | NO | YES | YES | YES | YES | YES | YES | NR | YES | NO | 64,28 | FAIR |
| Strohmeier et al., 2022 | YES | YES | YES | NO | NO | NO | NO | NO | YES | NO | YES | NR | NA | NO | 38,46 | POOR |
| Tang et al., 2023 | YES | YES | YES | NO | NO | NO | NO | YES | YES | NO | NO | NR | NA | NO | 38,46 | POOR |
| Tao et al., 2022 | YES | YES | NR | NO | NO | NO | NO | YES | YES | NO | YES | NR | NA | NO | 38,46 | POOR |
| Tian et al., 2023 | YES | YES | NR | NO | NO | YES | YES | YES | YES | YES | YES | NR | YES | YES | 76,92 | GOOD |
| Tobias & Chapanar, 2016 | YES | YES | NR | NO | NO | NO | NO | NO | YES | NO | YES | NR | NA | NO | 30,76 | POOR |
| Van Hoof et al., 2008 | YES | YES | NR | NO | NO | NO | NO | YES | YES | NO | YES | NR | NA | YES | 46,15 | POOR |
| Vannucci et al., 2021 | YES | YES | YES | YES | NO | YES | YES | YES | YES | NO | YES | NR | NO | NO | 64,28 | FAIR |
| Vargas & Monjardín, 2019 | YES | YES | NR | NO | NO | NO | NO | NO | YES | NO | YES | NR | NA | NO | 30,76 | POOR |
| Varsamis et al., 2022 | YES | YES | NR | NO | YES | NO | NO | YES | YES | NO | NO | NR | NA | YES | 46,15 | POOR |
| Veenstra et al., 2005 | YES | YES | YES | YES | NO | NO | NO | NO | YES | NO | NO | NR | NA | NO | 38,46 | POOR |
| Verešová & Mujkošová, 2020 | YES | YES | NR | NO | NO | NO | NO | NO | YES | NO | YES | NR | NA | NO | 30,76 | POOR |
| Wachs et al., 2020 | YES | YES | YES | NO | NO | NO | NO | YES | YES | NO | YES | NR | NA | NO | 46,15 | POOR |
| Wang & Chen, 2023 | YES | YES | NR | NO | NO | NO | NO | YES | YES | NO | YES | NR | NA | YES | 46,15 | POOR |
| Wang et al., 2009 | YES | YES | YES | NO | NO | NO | NO | YES | YES | NO | YES | NR | NA | NO | 46,15 | POOR |
| Wang et al., 2019 | YES | YES | YES | NO | NO | NO | NO | YES | YES | NO | YES | NR | NA | NO | 46,15 | POOR |
| Wang et al., 2022 | YES | YES | NR | NO | NO | NO | NO | NO | YES | NO | YES | NR | NA | NO | 30,76 | POOR |
| Wong & Konishi, 2021 | YES | YES | NR | NO | NO | NO | NO | YES | YES | NO | YES | NR | NA | YES | 46,15 | POOR |
| Wong & McBride, 2016 | YES | YES | NR | NO | NO | NO | NO | YES | YES | NO | YES | NR | NA | NO | 38,46 | POOR |
| Wright & Wachs, 2020 | YES | YES | YES | YES | YES | YES | YES | YES | YES | NO | CD | NR | YES | NO | 71,42 | FAIR |
| Wright et al., 2021 | YES | YES | NR | YES | NO | NO | NO | YES | YES | NO | YES | NR | NA | NO | 46,15 | POOR |
| Wright, 2016 (a) | YES | YES | NR | NO | NO | NO | NO | YES | YES | NO | CD | NR | NA | YES | 38,46 | POOR |
| Wright, 2016 (b) | YES | YES | YES | NO | NO | YES | YES | YES | YES | NO | YES | NR | YES | NO | 64,28 | FAIR |
| Wright, 2017 | YES | YES | YES | YES | NO | YES | YES | YES | YES | NO | CD | NR | YES | NO | 64,28 | FAIR |
| Wright, 2018 | YES | YES | YES | YES | NO | YES | YES | YES | YES | NO | CD | NR | YES | NO | 57,14 | FAIR |
| Wu et al., 2022 (a) | YES | YES | NR | NO | NO | YES | YES | YES | YES | YES | YES | NR | YES | YES | 76,92 | GOOD |
| Wu et al., 2022 (b) | YES | YES | NR | NO | YES | NO | NO | YES | YES | NO | YES | NR | NA | YES | 53,84 | FAIR |
| Wu et al., 2023 (a) | YES | YES | NR | NO | NO | NO | NO | YES | YES | NO | YES | NR | NA | YES | 46,15 | POOR |
| Wu et al., 2023 (b) | YES | YES | NR | NO | NO | YES | YES | YES | YES | NO | YES | NR | YES | YES | 69,23 | FAIR |
| Xiao et al., 2023 | YES | YES | NR | YES | YES | NO | NO | YES | YES | NO | YES | NR | NA | YES | 61,53 | FAIR |
| Yabko et al., 2008 | YES | YES | NR | NO | NO | NO | NO | YES | YES | NO | YES | NR | NA | YES | 46,15 | POOR |
| Ye et al., 2022 | YES | YES | NR | NO | NO | NO | NO | YES | YES | NO | YES | NR | NA | NO | 38,46 | POOR |
| Yu et al., 2023 | YES | YES | NR | NO | NO | YES | YES | YES | YES | NO | YES | NR | YES | YES | 61,53 | FAIR |
| Zhang et al., 2022 (a) | YES | YES | YES | YES | NO | NO | NO | YES | YES | NO | YES | NR | NA | NO | 53,84 | FAIR |
| Zhang et al., 2022 (b) | YES | YES | NR | NO | NO | YES | YES | YES | YES | NO | YES | NR | YES | NO | 61,53 | FAIR |
| Zhang et al., 2023 | YES | YES | NR | YES | NO | NO | NO | YES | YES | NO | YES | NR | NA | NO | 46,15 | POOR |
| Zhou & Li, 2021 | YES | YES | YES | YES | NO | NO | NO | NO | YES | NO | YES | NR | NA | YES | 53,84 | FAIR |
| Zhou et al., 2022 (a) | YES | YES | YES | NO | NO | NO | NO | YES | YES | NO | YES | NR | NA | YES | 53,84 | FAIR |
| Zhou et al., 2022 (b) | YES | YES | NR | NO | NO | YES | YES | YES | YES | YES | YES | NR | YES | YES | 69,23 | FAIR |
| Zhou et al., 2023 | YES | YES | YES | YES | NO | NO | NO | YES | YES | NO | YES | NR | NA | YES | 61,53 | FAIR |

***Note:***

Q1. Was the research question or objective in this paper clearly stated?

Q2. Was the study population clearly specified and defined?

Q3. Was the participation rate of eligible persons at least 50%?

Q4. Were all the subjects selected or recruited from the same or similar populations (including the same time period)? Were inclusion and exclusion criteria for being in the study prespecified and applied uniformly to all participants?

Q5. Was a sample size justification, power description, or variance and effect estimates provided?

Q6. For the analyses in this paper, were the exposure(s) of interest measured prior to the outcome(s) being measured?

Q7. Was the timeframe sufficient so that one could reasonably expect to see an association between exposure and outcome if it existed?

Q8. For exposures that can vary in amount or level, did the study examine different levels of the exposure as related to the outcome (e.g., categories of exposure, or exposure measured as continuous variable)?

Q9. Were the exposure measures (independent variables) clearly defined, valid, reliable, and implemented consistently across all study participants?

Q10. Was the exposure(s) assessed more than once over time?

Q11. Were the outcome measures (dependent variables) clearly defined, valid, reliable, and implemented consistently across all study participants?

Q12. Were the outcome assessors blinded to the exposure status of participants?

Q13. Was loss to follow-up after baseline 20% or less?

Q14. Were key potential confounding variables measured and adjusted statistically for their impact on the relationship between exposure(s) and outcome(s)?

**Appendix D - Table A.2.** List of excluded studies with reason (N=779)

| **REFERENCE** | **REASON** |
| --- | --- |
| Abdirahman, H. A., Bah, T. T., Shrestha, H. L., & Jacobsen, K. H. (2012). Bullying, mental health, and parental involvement among adolescents in the Caribbean. West Indian Med J, 61(5), 504-508. | No validated measure for PF |
| Abdirahman, H., Fleming, L. C., & Jacobsen, K. H. (2013). Parental involvement and bullying among middle school students in North Africa. EMHJ-Eastern Mediterranean Health Journal, 19 (3), 227-233, 2013. | No validated measure for PF |
| Aboagye, R. G., Seidu, A. A., Hagan Jr, J. E., Frimpong, J. B., Okyere, J., Cadri, A., & Ahinkorah, B. O. (2021, March). Bullying victimization among in-school adolescents in ghana: analysis of prevalence and correlates from the global school-based health survey. In Healthcare (Vol. 9, No. 3, p. 292). MDPI | Population not of interest |
| Abreu, R. L., Lefevor, G. T., Gonzalez, K. A., Barrita, A. M., & Watson, R. J. (2023). Bullying, depression, and parental acceptance in a sample of Latinx sexual and gender minority youth. Journal of LGBT Youth, 20(3), 585-602. | Outcome not of interest |
| Achuthan, K., Muthupalani, S., Kolil, V. K., & Madathil, K. C. (2022). Theoretical perspectives of parental influence on adolescent cyber behaviour: A bi-national Instagram-based study. Heliyon, 8(11). | Population not of interest |
| Acquah, E. O., Palonen, T., Lehtinen, E., & Laine, K. (2014). Social Status Profiles Among First Grade Children. Scandinavian Journal of Educational Research, 58(1), 73-92. | Outcome not of interest |
| Acquah, E. O., Wilson, M. L., & Doku, D. T. (2014). Patterns and correlates for bullying among young adolescents in Ghana. Social Sciences, 3(4), 827-840. | No validated measure for PF |
| Adams, R. E., Taylor, J. L., & Bishop, S. L. (2020). Brief Report: ASD-Related Behavior Problems and Negative Peer Experiences Among Adolescents with ASD in General Education Settings. Journal of autism and developmental disorders, 50(12), 4548-4552. | Outcome not of interest |
| Adegboyega, L. O., Okesina, F. A., & Jacob, O. A. (2017). Family Relationship and Bullying Behaviour among Students with Disabilities in Ogbomoso, Nigeria. International Journal of Instruction, 10(3), 241-256. | Outcome not of interest |
| Afifi, T. O., Taillieu, T., Salmon, S., Davila, I. G., Stewart-Tufescu, A., Fortier, J., ... & MacMillan, H. L. (2020). Adverse childhood experiences (ACEs), peer victimization, and substance use among adolescents. Child abuse & neglect, 106, 104504. | Outcome not of interest |
| Agnafors, S., Sydsjö, G., Svedin, C. G., & Bladh, M. (2023). Symptoms of depression and internalizing problems in early adulthood–associated factors from birth to adolescence. Nordic journal of psychiatry, 1-12. | Outcome not of interest |
| Aguado-Gracia, J., Mundo-Cid, P., Lopez-Seco, F., Acosta-García, S., Cortes-Ruiz, M. J., Vilella, E., & Masana-Marín, A. (2021). Lifetime victimization in children and adolescents with adhd. Journal of interpersonal violence, 36(5-6), NP3241-NP3262. | Outcome not of interest |
| Ahmed, E., & Braithwaite, V. (2004). Bullying and victimization: Cause for concern for both families and schools. Social Psychology of Education, 7(1), 35-54. | No response |
| AlBuhairan, F. S., Al Eissa, M., Alkufeidy, N., & Almuneef, M. (2016). Bullying in early adolescence: An exploratory study in Saudi Arabia. International journal of pediatrics and adolescent medicine, 3(2), 64-70. | Qualitative study |
| Alizadeh Maralani, F., Mirnasab, M., & Hashemi, T. (2019). The predictive role of maternal parenting and stress on pupils’ bullying involvement. Journal of interpersonal violence, 34(17), 3691-3710. | Couldn't provide correlation |
| Alizadeh Maralani, F., Mirnasab, M., & Hashemi, T. (2019). The predictive role of maternal parenting and stress on pupils’ bullying involvement. Journal of interpersonal violence, 34(17), 3691-3710. | Couldn't provide correlation |
| Aljasir, S. A., & Alsebaei, M. O. (2022). Cyberbullying and cybervictimization on digital media platforms: the role of demographic variables and parental mediation strategies. Humanities and Social Sciences Communications, 9(1), 1-9. | No response |
| Allison, S., Roeger, L., Smith, B., & Isherwood, L. (2014). Family histories of school bullying: implications for parent-child psychotherapy. Australasian psychiatry, 22(2), 149-153. | Outcome not of interest |
| Al-loughani, S., & Al-Shammari, E. T. (2022). Factors influencing the spread of cyberbullying among adolescents in private schools: Kuwait. International Journal of Innovation and Learning, 32(3), 262-282. | Full text not available |
| Alotaibi, N. B. (2019). Cyber Bullying and the Expected Consequences on the Students’ Academic Achievement. IEEE Access, 7, 153417-153431. | Outcome not of interest |
| Álvarez-García, D., Núñez, J. C., García, T., & Barreiro-Collazo, A. (2018). Individual, family, and community predictors of cyber-aggression among adolescents. European journal of psychology applied to legal context, 10(2), 79-88. | Outcome not of interest |
| Andersen, L. P., Labriola, M., Andersen, J. H., Lund, T., & Hansen, C. D. (2015). Bullied at school, bullied at work: a prospective study. BMC psychology, 3(1), 1-15. | Outcome not of interest |
| Annerbäck, E. M., Sahlqvist, L., Svedin, C. G., Wingren, G., & Gustafsson, P. A. (2012). Child physical abuse and concurrence of other types of child abuse in Sweden—Associations with health and risk behaviors. Child abuse & neglect, 36(7-8), 585-595. | No validated measure for PF |
| AntÓnio, R., & Moleiro, C. (2015). Social and parental support as moderators of the effects of homophobic bullying on psychological distress in youth. Psychology in the Schools, 52(8), 729-742. | Population not of interest |
| Armstrong, S. B., Dubow, E. F., & Domoff, S. E. (2019). Adolescent Coping: In-Person and Cyber-Victimization. Cyberpsychology: Journal of Psychosocial Research on Cyberspace, 13(4). | Outcome not of interest |
| Arnon, S., Klomek, A. B., Visoki, E., Moore, T. M., Argabright, S. T., DiDomenico, G. E., ... & Barzilay, R. (2022). Association of cyberbullying experiences and perpetration with suicidality in early adolescence. JAMA network open, 5(6), e2218746-e2218746. | Outcome not of interest |
| Aryayev, M. L., & Senkivska, L. I. (2021). Domestic and school violence in short stature children with growth hormone deficiency. | No validated measure for PF |
| Ashrafi, A., Feng, C. X., Neudorf, C., & Alphonsus, K. B. (2020). Bullying victimization among preadolescents in a community-based sample in Canada: a latent class analysis. BMC Research Notes, 13(1), 1-6. | No validated measure for PF |
| Athanasiades, C., Baldry, A. C., Kamariotis, T., Kostouli, M., & Psalti, A. (2016). The “net” of the internet: Risk factors for cyberbullying among secondary-school students in Greece. European Journal on Criminal Policy and Research, 22(2), 301-317. | No validated measure for PF |
| Athanasiou, K., Melegkovits, E., Andrie, E. K., Magoulas, C., Tzavara, C. K., Richardson, C., ... & Tsitsika, A. K. (2018). Cross-national aspects of cyberbullying victimization among 14–17-year-old adolescents across seven European countries. BMC Public Health, 18(1), 1-15. | No validated measure for PF |
| Atik, G., & Güneri, O. Y. (2013). Bullying and victimization: Predictive role of individual, parental, and academic factors. School Psychology International, 34(6), 658-673. | Couldn't provide correlation |
| Attar-Schwartz, S., & Fridman-Teutsch, A. (2018). Father support and adjustment difficulties among youth in residential care: The moderating role of peer victimization and gender. American Journal of Orthopsychiatry, 88(6), 701. | Full text not available |
| Ayas, T. (2012). The effect of parental attitudes on bullying and victimizing levels of secondary school students. Procedia-Social and Behavioral Sciences, 55, 226-231. | No response |
| Azeredo, C. M., Levy, R. B., Araya, R., & Menezes, P. R. (2015). Individual and contextual factors associated with verbal bullying among Brazilian adolescents. | Outcome not of interest |
| Azimi, A. M., & Daigle, L. E. (2020). Violent victimization: the role of social support and risky lifestyle. Violence and victims, 35(1), 20-38. | No validated measure for PF |
| Babarro Vélez, I., Andiarena Villaverde, A., Fano Ardanaz, E., García Vaquero, G., Lebeña Maluf, F. A., Arranz Freijó, E. B., & Ibarluzea Maurolagoitia, J. M. (2022). Do prepubertal hormones, 2D: 4D index and psychosocial context jointly explain 11-year-old preadolescents' involvement in bullying?. | Outcome not of interest |
| Babarro, I., Andiarena, A., Fano, E., Lertxundi, N., Vrijheid, M., Julvez, J., ... & Ibarluzea, J. (2020). Risk and protective factors for bullying at 11 years of age in a Spanish birth cohort study. International journal of environmental research and public health, 17(12), 4428. | Outcome not of interest |
| Bai, Q., Bai, S., Huang, Y., Hsueh, F. H., & Wang, P. (2020). Family incivility and cyberbullying in adolescence: A moderated mediation model. Computers in Human Behavior, 110, 106315. | Outcome not of interest |
| Baiden, P., Kuuire, V. Z., Shrestha, N., Tonui, B. C., Dako-Gyeke, M., & Peters, K. K. (2019). Bullying victimization as a predictor of suicidal ideation and suicide attempt among senior high school students in Ghana: results from the 2012 Ghana global school-based health survey. Journal of School Violence, 18(2), 300-317. | Outcome not of interest |
| Baker, A. C., Wallander, J. L., Elliott, M. N., & Schuster, M. A. (2023). Non-suicidal self-injury among adolescents: A structural model with socioecological connectedness, bullying victimization, and depression. Child Psychiatry & Human Development, 54(4), 1190-1208. | Outcome not of interest |
| Baldry, A. C. (2003). Bullying in schools and exposure to domestic violence. Child abuse & neglect, 27(7), 713-732. | Outcome not of interest |
| Baldry, A. C. (2004). The impact of direct and indirect bullying on the mental and physical health of Italian youngsters. Aggressive Behavior: Official Journal of the International Society for Research on Aggression, 30(5), 343-355. | No validated measure for PF |
| Baldry, A. C., & Farrington, D. P. (1998). Parenting influences on bullying and victimization. Legal and Criminological Psychology, 3(2), 237-254. | Outcome not of interest |
| Baldry, A. C., & Farrington, D. P. (2005). Protective factors as moderators of risk factors in adolescence bullying. Social psychology of education, 8(3), 263-284. | Population not of interest |
| Baldry, A. C., & Winkel, F. W. (2004). Mental and physical health of Italian youngsters directly and indirectly victimized at school and at home. International Journal of Forensic Mental Health, 3(1), 77-91. | Outcome not of interest |
| Baldry, A. C., Sorrentino, A., & Farrington, D. P. (2019). Cyberbullying and cybervictimization versus parental supervision, monitoring and control of adolescents' online activities. Children and Youth Services Review, 96, 302-307. | Population not of interest |
| Baldwin, J. R., Arseneault, L., Caspi, A., Moffitt, T. E., Fisher, H. L., Odgers, C. L., ... & Danese, A. (2019). Adolescent victimization and self-injurious thoughts and behaviors: A genetically sensitive cohort study. Journal of the American Academy of Child & Adolescent Psychiatry, 58(5), 506-513. | Outcome not of interest |
| Ball, H. A., Arseneault, L., Taylor, A., Maughan, B., Caspi, A., & Moffitt, T. E. (2008). Genetic and environmental influences on victims, bullies and bully‐victims in childhood. Journal of Child Psychology and Psychiatry, 49(1), 104-112. | Outcome not of interest |
| Bao, J., Li, H., Song, W., & Jiang, S. (2020). Being bullied, psychological pain and suicidal ideation among Chinese adolescents: A moderated mediation model. Children and youth services review, 109, 104744. | No validated measure for PF |
| Barranco, R., Gatti, U., Verde, A., & Rocca, G. (2023). Psychosocial factors of risk and protection associated with juvenile cyberbullying victimization: results from an international multi-city study (International Self-Report Delinquency Study 3, ISRD3). Psychiatry, psychology and law, 30(3), 397-417. | No response |
| Bartolo, M. G., Palermiti, A. L., Servidio, R., Musso, P., & Costabile, A. (2019). Mediating processes in the relations of parental monitoring and school climate with cyberbullying: The role of moral disengagement. Europe’s Journal of Psychology, 15(3), 568-594. | Population not of interest |
| Barzilay, S., Klomek, A. B., Apter, A., Carli, V., Wasserman, C., Hadlaczky, G., ... & Wasserman, D. (2017). Bullying victimization and suicide ideation and behavior among adolescents in Europe: A 10-country study. Journal of Adolescent Health, 61(2), 179-186. | Outcome not of interest |
| Bauer, N. S., Herrenkohl, T. I., Lozano, P., Rivara, F. P., Hill, K. G., & Hawkins, J. D. (2006). Childhood bullying involvement and exposure to intimate partner violence. Pediatrics, 118(2), e235-e242. | Outcome not of interest |
| Bayraktar, F. (2012). Bullying among adolescents in North Cyprus and Turkey: Testing a multifactor model. Journal of Interpersonal Violence, 27(6), 1040-1065. | Outcome not of interest |
| Bayraktar, F., Machackova, H., Dedkova, L., Cerna, A., & Ševčíková, A. (2015). Cyberbullying: The discriminant factors among cyberbullies, cybervictims, and cyberbully-victims in a Czech adolescent sample. Journal of interpersonal violence, 30(18), 3192-3216. | No response |
| Beijers, R., Ten Thije, I., Bolhuis, E., O'Donnell, K. J., Tollenaar, M. S., Shalev, I., ... & de Weerth, C. (2023). Cumulative risk exposure and child cellular aging in a Dutch low‐risk community sample. Psychophysiology, 60(4), e14205. | Outcome not of interest |
| Benatov, J. (2019). Parents’ Feelings, Coping Strategies and Sense of Parental Self-Efficacy When Dealing With Children’s Victimization Experiences. Frontiers in Psychiatry, 10, 700. | No validated measure for PF |
| Benavides Abanto, C. M., Jara-Almonte, J. L., Stuart, J., & La Riva, D. (2018). Bullying victimization among Peruvian children: the predictive role of parental maltreatment. Journal of interpersonal violence, 0886260518817780. | No validated measure for PF |
| Benavides Abanto, C. M., Jara-Almonte, J. L., Stuart, J., & La Riva, D. (2021). Bullying victimization among Peruvian children: The predictive role of parental maltreatment. Journal of interpersonal violence, 36(13-14), 6369-6390. | No validated measure for PF |
| Benítez-Sillero, J. D. D., Armada Crespo, J. M., Ruiz Córdoba, E., & Raya-González, J. (2021). Relationship between amount, type, enjoyment of physical activity and physical education performance with cyberbullying in adolescents. International journal of environmental research and public health, 18(4), 2038. | Outcome not of interest |
| Benton, T. D., Jones, J. D., Julye, S., Butler, L. L., & Boyd, R. C. (2021). ‘Web of Violence’, Depression, and Impairment in a Clinical Sample of Adolescents. Journal of family violence, 36(1), 17-26. | Outcome not of interest |
| Beran, T. N., Hughes, G., & Lupart, J. (2008). A model of achievement and bullying: Analyses of the Canadian National Longitudinal Survey of Children and Youth data. Educational Research, 50(1), 25-39. | No validated measure for PF |
| Berdondini, L., & Smith, P. K. (1996). Cohesion and power in the families of children involved in bully/victim problems at school: An Italian replication. Journal of Family Therapy, 18(1), 99-102. | No validated measure for PF |
| Bergeron, F. A., Blais, M., & Hébert, M. (2015). The role of parental support in the relationship between homophobic bullying, internalized homophobia and psychological distress among sexual-minority youths (SMY): a moderated mediation approach. Sante mentale au Quebec, 40(3), 109-127. | Another language |
| Bettencourt, A. F., & Farrell, A. D. (2013). Individual and contextual factors associated with patterns of aggression and peer victimization during middle school. Journal of youth and adolescence, 42(2), 285-302. | Outcome not of interest |
| Bhui, K., Silva, M. J., Harding, S., & Stansfeld, S. (2017). Bullying, social support, and psychological distress: findings from RELACHS cohorts of East London's White British and Bangladeshi adolescents. Journal of Adolescent Health, 61(3), 317-328. | No response |
| Bifulco, A., Schimmenti, A., Jacobs, C., Bunn, A., & Rusu, A. C. (2014). Risk factors and psychological outcomes of bullying victimization: A community-based study. Child Indicators Research, 7(3), 633-648. | Population not of interest |
| Bilić, V. (2014). The role of perceived social injustice and care received from the environment in predicting cyberbullying and cybervictimization. Medijska istraživanja: znanstveno-stručni časopis za novinarstvo i medije, 20(1), 101-125. | Full text not available |
| Bilodeau, F., Brendgen, M., Vitaro, F., Côté, S. M., Tremblay, R. E., Petit, D., ... & Boivin, M. (2020). Association between peer victimization and parasomnias in children: searching for relational moderators. Child Psychiatry & Human Development, 51(2), 268-280. | Outcome not of interest |
| Bilodeau, F., Brendgen, M., Vitaro, F., Côté, S. M., Tremblay, R. E., Touchette, E., ... & Boivin, M. (2018). Longitudinal association between peer victimization and sleep problems in preschoolers: the moderating role of parenting. Journal of Clinical Child & Adolescent Psychology, 47(sup1), S555-S568. | Outcome not of interest |
| Bilsky, S. A., Cole, D. A., Dukewich, T. L., Martin, N. C., Sinclair, K. R., Tran, C. V., ... & Maxwell, M. A. (2013). Does supportive parenting mitigate the longitudinal effects of peer victimization on depressive thoughts and symptoms in children?. Journal of Abnormal Psychology, 122(2), 406. | Outcome not of interest |
| Birkett, M., Newcomb, M. E., & Mustanski, B. (2015). Does it get better? A longitudinal analysis of psychological distress and victimization in lesbian, gay, bisexual, transgender, and questioning youth. Journal of Adolescent Health, 56(3), 280-285. | Population not of interest |
| Bishop, M. D., Ioverno, S., & Russell, S. T. (2023). Sexual minority youth’s mental health and substance use: The roles of victimization, cybervictimization, and non-parental adult support. Current Psychology, 42(6), 5075-5087. | Outcome not of interest |
| Biswas, T., Scott, J. G., Munir, K., Thomas, H. J., Huda, M. M., Hasan, M. M., ... & Mamun, A. A. (2020). Global variation in the prevalence of bullying victimisation amongst adolescents: Role of peer and parental supports. EClinicalMedicine, 20, 100276. | No response |
| Bjereld, Y. (2018). The challenging process of disclosing bullying victimization: A grounded theory study from the victim’s point of view. Journal of health psychology, 23(8), 1110-1118. | Outcome not of interest |
| Bjereld, Y., Daneback, K., & Petzold, M. (2017). Do bullied children have poor relationships with their parents and teachers? A cross-sectional study of Swedish children. Children and youth services review, 73, 347-351. | No validated measure for PF |
| Björkqvist, K., Österman, K., & Berg, P. (2011). Higher rates of victimization to physical abuse by adults found among victims of school bullying. Psychological reports, 109(1), 167-168. | Outcome not of interest |
| Black, S. A., & Jackson, E. (2007). Using bullying incident density to evaluate the Olweus Bullying Prevention Programme. School psychology international, 28(5), 623-638. | Outcome not of interest |
| Boel-Studt, S., & Renner, L. M. (2013). Individual and familial risk and protective correlates of physical and psychological peer victimization. Child abuse & neglect, 37(12), 1163-1174. | Outcome not of interest |
| Boel-Studt, S., & Renner, L. M. (2014). Child and family-level correlates of direct and indirect peer victimization among children ages 6–9. Child abuse & neglect, 38(6), 1051-1060. | Outcome not of interest |
| Bonanno, R. A., & Hymel, S. (2010). Beyond hurt feelings: Investigating why some victims of bullying are at greater risk for suicidal ideation. Merrill-Palmer Quarterly (1982-), 420-440. | Outcome not of interest |
| Bonnet, M., Goossens, F. A., & Schuengel, C. (2011). Parental strategies and trajectories of peer victimization in 4 to 5 year olds. Journal of school psychology, 49(4), 385-398. | No validated measure for PF |
| Bordin, I. A., Handegård, B. H., Paula, C. S., Duarte, C. S., & Rønning, J. A. Home, school, and community violence exposure and emotional and conduct problems among low-income adolescents: the moderating role of age and sex. | Outcome not of interest |
| Borowsky, I. W., Taliaferro, L. A., & McMorris, B. J. (2013). Suicidal thinking and behavior among youth involved in verbal and social bullying: Risk and protective factors. Journal of adolescent health, 53(1), S4-S12. | No validated measure for PF |
| Borraccino, A., Marengo, N., Dalmasso, P., Marino, C., Ciardullo, S., Nardone, P., ... & 2018 HBSC-Italia Group. (2022). Problematic social media use and cyber aggression in Italian adolescents: the remarkable role of social support. International journal of environmental research and public health, 19(15), 9763. | Couldn't provide correlation |
| Bowers, L., Smith, P. K., & Binney, V. (1992). Cohesion and power in the families of children involved in bully/victim problems at school. Journal of Family Therapy, 14(4), 371-387. | No validated measure for PF |
| Bowers, L., Smith, P. K., & Binney, V. (1994). Perceived family relationships of bullies, victims and bully/victims in middle childhood. Journal of social and personal relationships, 11(2), 215-232. | Outcome not of interest |
| Bowes, L., Arseneault, L., Maughan, B., Taylor, A., Caspi, A., & Moffitt, T. E. (2009). School, neighborhood, and family factors are associated with children's bullying involvement: A nationally representative longitudinal study. Journal of the American Academy of Child & Adolescent Psychiatry, 48(5), 545-553. | Outcome not of interest |
| Bowes, L., Maughan, B., Ball, H., Shakoor, S., Ouellet-Morin, I., Caspi, A., ... & Arseneault, L. (2013). Chronic bullying victimization across school transitions: the role of genetic and environmental influences. Development and psychopathology, 25(2). | Outcome not of interest |
| Bowes, L., Maughan, B., Caspi, A., Moffitt, T. E., & Arseneault, L. (2010). Families promote emotional and behavioural resilience to bullying: evidence of an environmental effect. Journal of child psychology and psychiatry, 51(7), 809-817. | Outcome not of interest |
| Bozan, K., Evgi̇n, D., & Gördeles Beşer, N. (2021). Relatıonship bullying in adolescent period with family functionalities and child behaviors. Psychology in the Schools, 58(8), 1451-1473. | No response |
| Bradbury, S. L., Dubow, E. F., & Domoff, S. E. (2018). How do adolescents learn cyber-victimization coping skills? An examination of parent and peer coping socialization. Journal of youth and adolescence, 47(9), 1866-1879. | Outcome not of interest |
| Bradshaw, C. P. (2014). The role of families in preventing and buffering the effects of bullying. JAMA pediatrics, 168(11), 991-993. | Qualitative study |
| Bravo-Sanzana, M., Bangdiwala, S. I., & Miranda, R. (2022). School violence negative effect on student academic performance: a multilevel analysis. International journal of injury control and safety promotion, 29(1), 29-41. | Outcome not of interest |
| Brendgen, M., Girard, A., Vitaro, F., Dionne, G., & Boivin, M. (2016). Personal and Familial Predictors of Peer Victimization Trajectories From Primary to Secondary School. Developmental psychology, 52(7), 1103-1114. | Outcome not of interest |
| Brendgen, M., Ouellet-Morin, I., Lupien, S. J., Vitaro, F., Dionne, G., & Boivin, M. (2017). Environmental influence of problematic social relationships on adolescents’ daily cortisol secretion: A monozygotic twin-difference study. Psychological Medicine, 47(3), 460-470. | Outcome not of interest |
| Brighi, A., Guarini, A., Melotti, G., Galli, S., & Genta, M. L. (2012). Predictors of victimisation across direct bullying, indirect bullying and cyberbullying. Emotional and behavioural difficulties, 17(3-4), 375-388. | Population not of interest |
| Broll, R., & Reynolds, D. (2020). Parental Responsibility, Blameworthiness, and Bullying: Parenting Style and Adolescents’ Experiences With Traditional Bullying and Cyberbullying. Criminal Justice Policy Review, 0887403420921443. | No response |
| Buelga, S., MartínezeFerrer, B., & Cava, M. (2017). Differences in family climate and family communication among cyberbullies, cybervictims, and cyber bullyevictims in adolescents. Computers in Human Behavior, 76, 164e173. | Methodological problems |
| Buils, R. F., Miedes, A. C., & Oliver, M. R. (2020). Effect of a cyberbullying prevention program integrated in the primary education curriculum. Revista de Psicodidáctica (English ed.), 25(1), 23-29. | Outcome not of interest |
| Burk, L. R., Park, J. H., Armstrong, J. M., Klein, M. H., Goldsmith, H. H., Zahn-Waxler, C., & Essex, M. J. (2008). Identification of early child and family risk factors for aggressive victim status in first grade. Journal of Abnormal Child Psychology, 36(4), 513-526. | Outcome not of interest |
| Burke, T., Sticca, F., & Perren, S. (2017). Everything’s gonna be alright! The longitudinal interplay among social support, peer victimization, and depressive symptoms. Journal of youth and adolescence, 46(9), 1999-2014. | Outcome not of interest |
| Buxton, D., Potter, M. P., & Bostic, J. Q. (2013). Coping strategies for child bully-victims. Psychiatric Annals, 43(3), 101-105. | Full text not available |
| Cabral, F., Fernandes, O. M., & Relva, I. C. (2022). Cybervictimization and Social Support Perceived by Adolescents and Young Adults. Avances en Psicología Latinoamericana, 40(3). | Another language |
| Caivano, O., Leduc, K., & Talwar, V. (2020). When you think you know: The effectiveness of restrictive mediation on parental awareness of cyberbullying experiences among children and adolescents. Cyberpsychology: Journal of Psychosocial Research on Cyberspace, 14(1). | No validated measure for PF |
| Calvete, E., Fernández-González, L., González-Cabrera, J. M., & Gámez-Guadix, M. (2018). Continued bullying victimization in adolescents: Maladaptive schemas as a mediational mechanism. Journal of youth and adolescence, 47(3), 650-660. | Outcome not of interest |
| Campbell, M., Straatmann, V. S., Lai, E. T. C., Potier, J., Pinto Pereira, S. M., & Wickham, S. L. (2019). Understanding social inequalities in children being bullied: UK Millennium Cohort Study findings. PLoS ONE, 14(5), e0217162. | Outcome not of interest |
| Cañas, E., Estévez, E., León-Moreno, C., & Musitu, G. (2020). Loneliness, family communication, and school adjustment in a sample of cybervictimized adolescents. International journal of environmental research and public health, 17(1), 335. | Couldn't provide correlation |
| Carrillo, L. A., Sabatini, C. S., Brar, R. K., Jagodzinski, J. E., Sabharwal, S., Delgado, E., & Livingston, K. S. (2021). The prevalence of bullying among pediatric orthopaedic patients. Journal of Pediatric Orthopaedics, 41(8), 463-466. | Full text not available |
| Casale, M., Cluver, L., Boyes, M., Toska, E., Gulaid, L., Armstrong, A., ... & Langwenya, N. (2021). Bullying and ART Nonadherence Among South African ALHIV: Effects, Risks, and Protective Factors. JAIDS Journal of Acquired Immune Deficiency Syndromes, 86(4), 436-444. | Full text not available |
| Casale, M., Cluver, L., Boyes, M., Toska, E., Gulaid, L., Armstrong, A., ... & Langwenya, N. (2021). Bullying and ART Nonadherence Among South African ALHIV: Effects, Risks, and Protective Factors. Journal of Acquired Immune Deficiency Syndromes (1999), 86(4), 436-444. | Population not of interest |
| Castellini, G., D'Anna, G., Rossi, E., Cassioli, E., Fabio, V., Caterina, S., ... & Ricca, V. (2021). Body weight trends in adolescents of Central Italy across 13 years: social, behavioural, and psychological correlates. ZEITSCHRIFT FüR GESUNDHEITSWISSENSCHAFTEN, 1-11. | Outcome not of interest |
| Cava, M. J. (2011). Family, teachers, and peers: keys for supporting victims of bullying. Psychosocial intervention, 20(2), 183-192. | Another language |
| Cénat, J. M., Blais, M., Lavoie, F., Caron, P. O., & Hébert, M. (2018). Cyberbullying victimization and substance use among Quebec high schools students: The mediating role of psychological distress. Computers in Human Behavior, 89, 207-212. | Population not of interest |
| Cenkseven Onder, F., & Yurtal, F. (2008). An Investigation of the Family Characteristics of Bullies, Victims, and Positively Behaving Adolescents. Educational Sciences: Theory and Practice, 8(3), 821-832. | Outcome not of interest |
| Cenkseven Onder, F., & Yurtal, F. (2008). An Investigation of the Family Characteristics of Bullies, Victims, and Positively Behaving Adolescents. Educational Sciences: Theory and Practice, 8(3), 821-832. | No response |
| Cerezo, F., Sanchez, C., Ruiz, C., & Arense, J. J. (2015). Adolescents and preadolescents’ roles on bullying, and its relation with social climate and parenting styles. Revista de Psicodidáctica, 20(1), 139-155. | No response |
| Cerimoniale, G., Dalpiaz, I., Becherucci, P., Malorgio, E., Ceschin, F., Rosati, G. V., ... & Oretti, C. (2023). The digital child: A cross‐sectional survey study on the access to electronic devices in paediatrics. Acta Paediatrica, 112(8), 1792-1803. | Population not of interest |
| Cerna, A., Machackova, H., & Dedkova, L. (2016). Whom to trust: The role of mediation and perceived harm in support seeking by cyberbullying victims. Children & Society, 30(4), 265-277. | No validated measure for PF |
| Çevik, Ö., Rıdvan, A. T. A., & Çevik, M. Bullying and victimization among Turkish adolescents: the predictive role of problematic internet use, school burnout and parental monitoring. Education and Information Technologies, 1-28. | Couldn't provide correlation |
| Chai, L., Xue, J., & Han, Z. (2020). School bullying victimization and self-rated health and life satisfaction: The mediating effect of relationships with parents, teachers, and peers. Children and Youth Services Review, 117, 105281. | No validated measure for PF |
| Chan, H. C. O., & Wong, D. S. (2015). The overlap between school bullying perpetration and victimization: Assessing the psychological, familial, and school factors of Chinese adolescents in Hong Kong. Journal of child and Family Studies, 24(11), 3224-3234. | No validated measure for PF |
| Chan, H. C. O., & Wong, D. S. (2017). Coping with cyberbullying victimization: An exploratory study of Chinese adolescents in Hong Kong. International journal of law, crime and justice, 50, 71-82. | No validated measure for PF |
| Chan, H. C., & Wong, D. S. (2020). The overlap between cyberbullying perpetration and victimisation: exploring the psychosocial characteristics of Hong Kong adolescents. Asia Pacific Journal of Social Work and Development, 30(3), 164-180. | No validated measure for PF |
| Chan, J. Y., Harlow, A. J., Kinsey, R., Gerstein, L. H., & Fung, A. L. C. (2018). The examination of authoritarian parenting styles, specific forms of peer-victimization, and reactive aggression in Hong Kong Youth. School psychology international, 39(4), 378-399. | Outcome not of interest |
| Chang, F. C., Miao, N. F., Chiu, C. H., Chen, P. H., Lee, C. M., Chiang, J. T., & Chuang, H. Y. (2016). Urban–rural differences in parental Internet mediation and adolescents’ Internet risks in Taiwan. Health, Risk & Society, 18(3-4), 188-204. | No response |
| Chang, Q., Xing, J., Ho, R. T., & Yip, P. S. (2019). Cyberbullying and suicide ideation among Hong Kong adolescents: the mitigating effects of life satisfaction with family, classmates and academic results. Psychiatry research, 274, 269-273. | Outcome not of interest |
| Chao, M., Lei, J., He, R., Jiang, Y., & Yang, H. (2023). TikTok use and psychosocial factors among adolescents: Comparisons of non-users, moderate users, and addictive users. Psychiatry Research, 325, 115247. | Outcome not of interest |
| Charles, R., Brand, P. L., Gilchrist, F. J., Wildhaber, J., & Carroll, W. (2022). Why are children with asthma bullied? A risk factor analysis. Archives of disease in childhood, 107(6), 612-615. | Outcome not of interest |
| Chaux, E., & Castellanos, M. (2015). Money and age in schools: Bullying and power imbalances. Aggressive behavior, 41(3), 280-293. | No validated measure for PF |
| Che, Y., Lu, J., Fang, D., Ran, H., Wang, S., Liang, X., ... & Xiao, Y. (2022). Association between school bullying victimization and self-harm in a sample of Chinese children and adolescents: The mediating role of perceived social support. Frontiers in Public Health, 10. | No response |
| Chen, J. K., & Astor, R. A. (2012). School variables as mediators of personal and family factors on school violence in Taiwanese junior high schools. Youth & Society, 44(2), 175-200. | Outcome not of interest |
| Chen, J. K., Chang, C. W., Wang, Z., Wang, L. C., & Wei, H. S. (2021). Cyber deviance among adolescents in Taiwan: Prevalence and correlates. Children and Youth Services Review, 126, 106042. | No validated measure for PF |
| Chen, J. K., Pan, Z., & Wang, L. C. (2021). Parental beliefs and actual use of corporal punishment, school violence and bullying, and depression in early adolescence. International journal of environmental research and public health, 18(12), 6270. | Outcome not of interest |
| Chen, J. K., Wang, S. C., & Chen, Y. W. (2023). Social Relationships as Mediators of Material Deprivation, School Bullying Victimization, and Subjective Well-Being among Children Across 25 Countries: A Global and Cross-National Perspective. Applied Research in Quality of Life, 1-26. | Outcome not of interest |
| Chen, J. K., Wang, S. C., & Chen, Y. W. (2023). Social Relationships as Mediators of Material Deprivation, School Bullying Victimization, and Subjective Well-Being among Children Across 25 Countries: A Global and Cross-National Perspective. Applied Research in Quality of Life, 1-26. | Outcome not of interest |
| Chen, J. K., Wang, S. C., Chen, Y. W., & Huang, T. H. Family Climate, Social Relationships With Peers and Teachers at School, and School Bullying Victimization Among Third Grade Students in Elementary Schools in Taiwan. School Mental Health, 1-10. | No validated measure for PF |
| Chen, J. K., Wu, C., & Wei, H. S. (2020). Personal, family, school, and community factors associated with student victimization by teachers in Taiwanese junior high schools: A multi-informant and multilevel analysis. Child abuse & neglect, 99, 104246. | Outcome not of interest |
| Chen, J. K., Yang, B., Lin, C. Y., & Wang, L. C. (2023). Affiliation with delinquent peers as a mediator of the relationships between family conflict and school bullying: a short-term longitudinal panel study. Journal of interpersonal violence, 08862605231175517. | Outcome not of interest |
| Chen, L., Liu, X., & Tang, H. (2023). The interactive effects of parental mediation strategies in preventing cyberbullying on social media. Psychology Research and Behavior Management, 1009-1022. | No response |
| Chen, M., & Chan, K. L. (2016). Parental absence, child victimization, and psychological well-being in rural China. Child Abuse & Neglect, 59, 45-54. | Outcome not of interest |
| Chen, Q., & Zhu, Y. (2022). Cyberbullying victimisation among adolescents in China: Coping strategies and the role of self‐compassion. Health & Social Care in the Community, 30(3), e677-e686. | Outcome not of interest |
| Chen, Q., Lo, C. K., Zhu, Y., Cheung, A., Chan, K. L., & Ip, P. (2018). Family poly-victimization and cyberbullying among adolescents in a Chinese school sample. Child abuse & neglect, 77, 180-187. | Outcome not of interest |
| Chen, X., Jiang, J., Li, Z., Gong, Y., & Du, J. (2022). Influence of family cohesion on Chinese adolescents’ engagement in school bullying: A moderated mediation model. Frontiers in psychology, 13, 1040559. | Outcome not of interest |
| Chen, X., Li, L., Lv, G., & Li, H. (2021). Parental behavioral control and bullying and victimization of rural adolescents in China: the roles of deviant peer affiliation and gender. International journal of environmental research and public health, 18(9), 4816. | Outcome not of interest |
| Chen, Z., Ren, S., He, R., Liang, Y., Tan, Y., Liu, Y., ... & Tang, J. (2023). Prevalence and associated factors of depressive and anxiety symptoms among Chinese secondary school students. BMC psychiatry, 23(1), 580. | Outcome not of interest |
| Cheng, A. W., Chou, Y. C., & Lin, F. G. (2019). Psychological Distress in Bullied Deaf and Hard of Hearing Adolescents. Journal of deaf studies and deaf education, 24(4), 366-377. | No validated measure for PF |
| Cheng, S. T., Cheung, K. C., & Cheung, C. K. (2008). Peer victimization and depression among Hong Kong adolescents. Journal of clinical psychology, 64(6), 766-776. | Outcome not of interest |
| Cheng, T. C., & Lo, C. C. (2022). Factors Related to Immigrant/Nonimmigrant Children’s Experience of Being Bullied: An Analysis Using the Multiple Disadvantage Model. Community mental health journal, 58(4), 689-700. | No validated measure for PF |
| Cheng, T. C., & Lo, C. C. (2023). Factors Contributed to Bully Victimization Among 6-to 13-Year-Old Children Cared by Parents or Relative/Nonrelative Caregivers: A Multiple Disadvantage Model. Families in Society, 10443894231182496. | Full text not available |
| Chervonsky, E., & Hunt, C. (2019). Emotion regulation, mental health, and social wellbeing in a young adolescent sample: A concurrent and longitudinal investigation. Emotion, 19(2), 270. | Outcome not of interest |
| Chester, K. L., Magnusson, J., Klemera, E., Spencer, N. H., & Brooks, F. (2019). The mitigating role of ecological health assets in adolescent cyberbullying victimization. Youth & Society, 51(3), 291-317. | No response |
| Chiu, Y. L., Kao, S., Tou, S. W., & Lin, F. G. (2017). Effect of personal characteristics, victimization types, and family-and school-related factors on psychological distress in adolescents with intellectual disabilities. Psychiatry research, 248, 48-55. | No validated measure for PF |
| Cho, S., & Galehan, J. (2020). Stressful life events and negative emotions on delinquency among Korean youth: An empirical test of general strain theory assessing longitudinal mediation analysis. International journal of offender therapy and comparative criminology, 64(1), 38-62. | Outcome not of interest |
| Cho, S., Hong, J. S., Sterzing, P. R., & Woo, Y. (2017). Parental attachment and bullying in South Korean adolescents: Mediating effects of low self-control, deviant peer associations, and delinquency. Crime & Delinquency, 63(9), 1168-1188. | Outcome not of interest |
| Choi, B., & Park, S. (2018). Who Becomes a Bullying Perpetrator After the Experience of Bullying Victimization? The Moderating Role of Self-esteem. *Journal of Youth and Adolescence*, *47*(11), 2414–2423. | Secondary report |
| Choi, J., Han, C. K., & Kang, S. (2022). Bullying victimization, social withdrawal, and problematic internet use: A moderated mediation model of parental involvement in education among Korean adolescents. Child Indicators Research, 15(5), 1889-1903. | Outcome not of interest |
| Choi, Y. J., Shin, S. Y., & Lee, J. (2022). Change in factors affecting cyberbullying of Korean Elementary School students during the COVID-19 pandemic. International journal of environmental research and public health, 19(17), 11046. | Outcome not of interest |
| Chou, W. J., Liu, T. L., Yang, P., Yen, C. F., & Hu, H. F. (2018). Bullying victimization and perpetration and their correlates in adolescents clinically diagnosed with ADHD. Journal of attention disorders, 22(1), 25-34. | No response |
| Christie‐Mizell, C. A. (2003). Bullying: The consequences of interparental discord and child's self‐concept. Family Process, 42(2), 237-251. | Outcome not of interest |
| Chrysanthou, G. M., & Vasilakis, C. (2020). Protecting the mental health of future adults: Disentangling the determinants of adolescent bullying victimisation. Social Science & Medicine, 253, 112942. | No validated measure for PF |
| Chui, W. H., Weng, X., & Khiatani, P. V. (2020). Associations among bullying victimization, family dysfunction, negative affect, and bullying perpetration in Macanese adolescents. International journal of offender therapy and comparative criminology, 0306624X20983741. | Full text not available |
| Chun, J., Lee, H. K., Jeon, H., Kim, J., & Lee, S. (2023). Impact of COVID-19 on Adolescents’ Smartphone Addiction in South Korea. Social Work in Public Health, 38(4), 268-280. | No validated measure for PF |
| Chun, J., Lee, S., & Kim, J. (2021). Conceptualizing the protective factors of cyberbullying victimization in Korean adolescents. School mental health, 1-14. | Outcome not of interest |
| Chung, J. Y., Sun, M. S., & Kim, H. J. (2018). What makes bullies and victims in Korean elementary schools?. Children and Youth Services Review, 94, 132-139. | No validated measure for PF |
| Cimen, I. D. (2018). Cyberbullying in adolescents, the effect of internet parenting styles and family functioning/Ergenlerde siber zorbalik, internet aile tutumu ve aile islevselliginin etkisi. Anadolu Psikiyatri Dergisi, 19(4), 397-405. | Full text not available |
| Claes, L., Luyckx, K., Baetens, I., Van de Ven, M., & Witteman, C. (2015). Bullying and victimization, depressive mood, and non-suicidal self-injury in adolescents: The moderating role of parental support. Journal of child and family studies, 24(11), 3363-3371. | Population not of interest |
| Clarke, A., Olive, P., Akooji, N., & Whittaker, K. (2020). Violence exposure and young people’s vulnerability, mental and physical health. International journal of public health, 1-10. | Outcome not of interest |
| Clear, E. R., Coker, A. L., Cook-Craig, P. G., Bush, H. M., Garcia, L. S., Williams, C. M., ... & Fisher, B. S. (2014). Sexual harassment victimization and perpetration among high school students. Violence Against Women, 20(10), 1203-1219. | Outcome not of interest |
| Cluver, L., Bowes, L., & Gardner, F. (2010). Risk and protective factors for bullying victimization among AIDS-affected and vulnerable children in South Africa. Child abuse & neglect, 34(10), 793-803. | Population not of interest |
| Coe, J. L., Davies, P. T., Hentges, R. F., & Sturge-Apple, M. L. (2020). Detouring in the family system as an antecedent of children’s adjustment problems. Journal of Family Psychology, 34(7), 814. | Outcome not of interest |
| Cole, D. A., Martin, N. C., Sterba, S. K., Sinclair-McBride, K., Roeder, K. M., Zelkowitz, R., & Bilsky, S. A. (2014). Peer victimization (and harsh parenting) as developmental correlates of cognitive reactivity, a diathesis for depression. Journal of abnormal psychology, 123(2), 336. | Outcome not of interest |
| Cole, D. A., Sinclair-McBride, K. R., Zelkowitz, R., Bilsk, S. A., Roeder, K., & Spinelli, T. Peer Victimization and Harsh Parenting Predict Cognitive Diatheses for Depression in Children and Adolescents. | Outcome not of interest |
| Cole-Lewis, Y. C., Gipson, P. Y., Opperman, K. J., Arango, A., & King, C. A. (2016). Protective role of religious involvement against depression and suicidal ideation among youth with interpersonal problems. Journal of religion and health, 55(4), 1172-1188. | Outcome not of interest |
| Connell, N. M., Morris, R. G., & Piquero, A. R. (2016). Predicting Bullying: Exploring the Contributions of Childhood Negative Life Experiences in Predicting Adolescent Bullying Behavior. International journal of offender therapy and comparative criminology, 60(9), 1082-1096. | Outcome not of interest |
| Conners‐Burrow, N. A., Johnson, D. L., Whiteside‐Mansell, L., McKelvey, L., & Gargus, R. A. (2009). Adults matter: Protecting children from the negative impacts of bullying. Psychology in the Schools, 46(7), 593-604. | No response |
| Connolly, E. J., Kavish, N., & Cooke, E. M. (2019). Testing the causal hypothesis that repeated bullying victimization leads to lower levels of educational attainment: A sibling-comparison analysis. Journal of School Violence, 18(2), 272-284. | Outcome not of interest |
| Connolly, I., & O'Moore, M. (2003). Personality and family relations of children who bully. Personality and individual differences, 35(3), 559-567. | Outcome not of interest |
| Cortés-Pascual, A., Cano-Escorianza, J., Elboj-Saso, C., & Iñiguez-Berrozpe, T. (2020). Positive relationships for the prevention of bullying and cyberbullying: A study in Aragón (Spain). International Journal of Adolescence and Youth, 25(1), 182-199. | Outcome not of interest |
| Costa, M. R. D., Xavier, C. C., Andrade, A. C. D. S., Proietti, F. A., & Caiaffa, W. T. (2015). Bullying among adolescents in a Brazilian urban center–“Health in Beagá” Study. Revista de saude publica, 49, 56. | Outcome not of interest |
| Cotter, K. L., Wu, Q., & Smokowski, P. R. (2016). Longitudinal risk and protective factors associated with internalizing and externalizing symptoms among male and female adolescents. Child Psychiatry & Human Development, 47(3), 472-485. | Outcome not of interest |
| Cross, D., & Barnes, A. (2014). Using systems theory to understand and respond to family influences on children's bullying behavior: Friendly schools friendly families program. Theory into practice, 53(4), 293-299. | Qualitative study |
| Cross, D., Lester, L., Pearce, N., Barnes, A., & Beatty, S. (2018). A group randomized controlled trial evaluating parent involvement in whole-school actions to reduce bullying. The Journal of Educational Research, 111(3), 255-267. | Outcome not of interest |
| Cross, D., Monks, H., Hall, M., Shaw, T., Pintabona, Y., Erceg, E., ... & Lester, L. (2011). Three‐year results of the Friendly Schools whole‐of‐school intervention on children's bullying behaviour. British Educational Research Journal, 37(1), 105-129. | Outcome not of interest |
| Cross, D., Shaw, T., Epstein, M., Pearce, N., Barnes, A., Burns, S., ... & Runions, K. (2018). Impact of the Friendly Schools whole‐school intervention on transition to secondary school and adolescent bullying behaviour. European Journal of Education, 53(4), 495-513. | Outcome not of interest |
| Crouch, E., Figas, K., Radcliff, E., & Hunt, E. T. (2023). Examining bullying victimization, bullying perpetration, and positive childhood experiences. Journal of school health. | Full text not available |
| Cui, K., Xie, H., & Peng, H. (2023). How Past Cyber Victimization Affects Bystanders’ Position Taking in Offline Bullying Situations: A Moderated Mediation Model of Self-concept and Social Support. School mental health, 15(2), 416-430. | Outcome not of interest |
| Cunning, A., Campbell, K., Chermack, R., Weiss, A., & Rancourt, D. (2023). 61. Predictors and Correlates of Bullying Perpetration and Victimization Among Adolescent Bariatric Surgery Candidates. Journal of Adolescent Health, 72(3), S38. | Full text not available |
| Cunningham, S., Goff, C., Bagby, R. M., Stewart, J. G., Larocque, C., Mazurka, R., ... & Harkness, K. L. (2019). Maternal-versus paternal-perpetrated maltreatment and risk for sexual and peer bullying revictimization in young women with depression. Child abuse & neglect, 89, 111-121. | Population not of interest |
| D’Anna, G., Lazzeretti, M., Castellini, G., Ricca, V., Cassioli, E., Rossi, E., ... & Voller, F. (2022). Risk of eating disorders in a representative sample of Italian adolescents: prevalence and association with self-reported interpersonal factors. Eating and Weight Disorders-Studies on Anorexia, Bulimia and Obesity, 27(2), 701-708. | Population not of interest |
| D’Urso, G., Symonds, J., Sloan, S., & Devine, D. (2022). Bullies, victims, and meanies: the role of child and classmate social and emotional competencies. Social Psychology of Education, 25(1), 293-312. | Outcome not of interest |
| Dardas, L. A., Shahrour, G., Al-Khayat, A., Sweis, N., & Pan, W. (2022). Family environment and coping strategies as mediators of school bullying involvement. Journal of school violence, 21(4), 504-516. | No response |
| Davis, J. P., Ingram, K. M., Merrin, G. J., & Espelage, D. L. (2018). Exposure to parental and community violence and the relationship to bullying perpetration and victimization among early adolescents: A parallel process growth mixture latent transition analysis. Scandinavian Journal of Psychology, 61(1), 77-89. | Outcome not of interest |
| Davis, K., & Koepke, L. (2016). Risk and protective factors associated with cyberbullying: Are relationships or rules more protective?. Learning, Media and Technology, 41(4), 521-545. | Population not of interest |
| de Alcantara, S. C., González-Carrasco, M., Montserrat, C., Casas, F., Viñas-Poch, F., & de Abreu, D. P. (2019). Peer violence, school environment and developmental contexts: its effects on well-being/Violencia entre pares, clima escolar e contextos de desenvolvimento: suas implicacoes no bem-estar. Ciencia & saude coletiva, 24(2), 509-523. | Another language |
| De Looze, M. E., Cosma, A. P., Vollebergh, W. A. M., Duinhof, E. L., de Roos, S. A., van Dorsselaer, S., ... & Stevens, G. W. J. M. (2020). Trends over Time in Adolescent Emotional Wellbeing in the Netherlands, 2005-2017: Links with Perceived Schoolwork Pressure, Parent-Adolescent Communication and Bullying Victimization. Journal of Youth and Adolescence, 49(10), 2124. | Outcome not of interest |
| De Luca, S. M., Caramanis, C., & Zhang, A. (2021). A longitudinal study examining the associations of bullying victimization and suicidal ideation among sexual minority adolescents. Suicide and Life-Threatening Behavior, 51(6), 1138-1147. doi:10.1111/sltb.12796 | Outcome not of interest |
| de Oliveira, I. R., Matos-Ragazzo, A. C., Zhang, Y., Vasconcelos, N. M., Velasquez, M. L., Reis, D., ... & Cecil, C. A. (2018). Disentangling the mental health impact of childhood abuse and neglect: a replication and extension study in a Brazilian sample of high-risk youth. Child abuse & neglect, 80, 312-323. | Outcome not of interest |
| de Oliveira, W. A., Da Silva, J. L., Querino, R. A., Caravita, S. C. S., & Silva, M. A. I. (2019). FAMILY VARIABLES AND BULLYING AMONG BRAZILIAN ADOLESCENTS: A MIXED STUDY. Behavioral Psychology/Psicologia Conductual, 27(1). | Population not of interest |
| del Barco, B. L., Castaño, E. F., & del Río, M. I. P. (2015). Parental acceptance-rejection and profiles of victimization and ag-gression in bullying situations. Anales de Psicologia, 31(2), 600. | Another language |
| Del Rey, R., Casas, J. A., & Ortega, R. (2016). Impact of the ConRed program on different cyberbulling roles. Aggressive behavior, 42(2), 123-135. | Outcome not of interest |
| Deng, C., Gong, X., Liu, W., Huebner, E. S., & Tian, L. (2022). Co-developmental trajectories of interpersonal relationships in middle childhood to early adolescence: Associations with traditional bullying and cyberbullying. Journal of Social and Personal Relationships, 39(9), 2701-2723. | No response |
| Di Terresena, L. G., Diolosà, C., Inga, F., & Caruso, S. (2010). Bullying: Disease or Social Phenomenon? An Experimental Research. Rivista di psichiatria, 45(2), 107-111. | Full text not available |
| Dickson, D. J., Laursen, B., Valdes, O., & Stattin, H. (2019). Derisive parenting fosters dysregulated anger in adolescent children and subsequent difficulties with peers. Journal of youth and adolescence, 48(8), 1567-1579. | No validated measure for PF |
| Dilmaç, B., & Aydoğan, D. (2010). Parental attitudes as a predictor of cyber bullying among primary school children. World Academy of Science, Engineering and Technology, 67, 167-171. | No response |
| Ding, Y., Li, D., Li, X., Xiao, J., Zhang, H., & Wang, Y. (2020). Profiles of adolescent traditional and cyber bullying and victimization: The role of demographic, individual, family, school, and peer factors. Computers in Human Behavior, 111, 106439. | Population not of interest |
| dos Santos Mesquita, C., & da Costa Maia, Â. (2019). A step toward a better understanding of the relationship between victimization and emotional distress: indirect effect of adult attachment and interaction with household dysfunction. Journal of interpersonal violence, 34(15), 3252-3289. | Population not of interest |
| Doty, J. L., Gower, A. L., Rudi, J. H., McMorris, B. J., & Borowsky, I. W. (2017). Patterns of bullying and sexual harassment: Connections with parents and teachers as direct protective factors. Journal of youth and adolescence, 46(11), 2289-2304. | No validated measure for PF |
| Duncan, R. D. (1999). Maltreatment by parents and peers: The relationship between child abuse, bully victimization, and psychological distress. Child maltreatment, 4(1), 45-55. | Population not of interest |
| Duru, E., & Balkis, M. (2018). Exposure to school violence at school and mental health of victimized adolescents: The mediation role of social support. Child abuse & neglect, 76, 342-352. | Outcome not of interest |
| Eden, S., Heiman, T., & Olenik-Shemesh, D. (2016). Bully versus victim on the internet: The correlation with emotional-social characteristics. Education and Information Technologies, 21(3), 699-713. | Outcome not of interest |
| Eden, S., Heiman, T., Olenik-Shemesh, D., & Yablon, Y. B. (2023). Cyberbullying and PIU Among Adolescents Before and During COVID-19 Pandemic: The Association With Adolescents Relationships. Youth & Society, 0044118X231169493. | Couldn't provide correlation |
| Elgar, F. J., Napoletano, A., Saul, G., Dirks, M. A., Craig, W., Poteat, V. P., ... & Koenig, B. W. (2014). Cyberbullying Victimization and Mental Health in Adolescents and the Moderating Role of Family Dinners. | Outcome not of interest |
| Eliot, M., & Cornell, D. G. (2009). Bullying in middle school as a function of insecure attachment and aggressive attitudes. School Psychology International, 30(2), 201-214. | Outcome not of interest |
| Erath, S. A., Pettit, G. S., & Troop-Gordon, W. (2021). Direct and compensatory parental responses to peer victimization. The Journal of Early Adolescence, 41(1), 197-217. | Qualitative study |
| Erdogan, Y., Hammami, N., & Elgar, F. J. (2023). Bullying, Family Support, and Life Satisfaction in Adolescents of Single-Parent Households in 42 Countries. Child Indicators Research, 16(2), 739-753. | No response |
| Erginoz, E., Alikasifoglu, M., Ercan, O., Uysal, O., Alp, Z., Ocak, S., ... & Albayrak Kaymak, D. (2015). The role of parental, school, and peer factors in adolescent bullying involvement: Results from the Turkish HBSC 2005/2006 study. Asia Pacific Journal of Public Health, 27(2), NP1591-NP1603. | Outcome not of interest |
| Ersozlu, Z., Wildy, H., Ersozlu, A., Lawrence, D., Karakus, M., Sorgo, A., ... & Chun-Yen, C. H. A. N. G. (2020). Self-esteem, bullying perpetration/victimization and perceived parental support in a nationally representative sample of australian students. Revista de Cercetare si Interventie Sociala, 69, 49. | No validated measure for PF |
| Eslea, M., & Smith, P. K. (2000). Pupil and parent attitudes towards bullying in primary schools. European Journal of Psychology of Education, 15(2), 207-219. | Outcome not of interest |
| Espelage, D. L., Bosworth, K., & Simon, T. R. (2000). Examining the social context of bullying behaviors in early adolescence. Journal of counseling & development, 78(3), 326-333. | Outcome not of interest |
| Espelage, D. L., Van Ryzin, M. J., & Holt, M. K. (2018). Trajectories of bully perpetration across early adolescence: Static risk factors, dynamic covariates, and longitudinal outcomes. Psychology of violence, 8(2), 141. | No response |
| Estévez, E., Jiménez, T. I., Moreno, D., & Musitu, G. (2013). An analysis of the relationship between victimization and violent behavior at school. The Spanish Journal of Psychology, 16. | Outcome not of interest |
| Estévez-García, J. F., Cañas, E., & Estévez, E. (2023). Non-disclosure and suicidal ideation in adolescent victims of bullying: an analysis from the family and school context. Psychosocial intervention, 32(3), 191. | Outcome not of interest |
| Eugene, D. R., Du, X., & Kim, Y. K. (2021). School climate and peer victimization among adolescents: A moderated mediation model of school connectedness and parental involvement. Children and Youth Services Review, 121, 105854. | Outcome not of interest |
| Eze, J. E., Chukwuorji, J. C., Ettu, P. C., Zacchaeus, E. A., Iorfa, S. K., & Nwonyi, S. K. (2019). Bullying and suicide ideation: testing the buffering hypothesis of social support in a sub-Saharan African sample. Journal of Child & Adolescent Trauma, 1-9. | Outcome not of interest |
| Fan, H., Xue, L., Xiu, J., Chen, L., & Liu, S. (2023). Harsh parental discipline and school bullying among Chinese adolescents: The role of moral disengagement and deviant peer affiliation. Children and Youth Services Review, 145, 106767. | Outcome not of interest |
| Figueira, M. D. P., Okada, L. M., Leite, T. H., Azeredo, C. M., & Marques, E. S. (2022). Association between parental supervision and bullying victimization and perpetration in Brazilian adolescents, Brazilian National Survey of Student’s Health 2015. Epidemiologia e Serviços de Saúde, 31, e2021778. | No validated measure for PF |
| Figula, E., Margitics, F., Pauwlik, Z., & Szatmári, Á. (2011). School bullying: background factors of the victims, bullies, bully/victims in family socialization. Mentálhigiéné és Pszichoszomatika, 12(1), 47-72. | Full text not available |
| Finkelhor, D., & Dziuba-Leatherman, J. (1995). Victimization prevention programs: A national survey of children's exposure and reactions. Child abuse & neglect, 19(2), 129-139. | Outcome not of interest |
| Fisher, H. L., Caspi, A., Moffitt, T. E., Wertz, J., Gray, R., Newbury, J., ... & Arseneault, L. (2015). Measuring adolescents' exposure to victimization: the environmental risk (E-Risk) longitudinal twin study. Development and psychopathology, 27(4pt2), 1399-1416. | Outcome not of interest |
| Fisher, H. L., Moffitt, T. E., Houts, R. M., Belsky, D. W., Arseneault, L., & Caspi, A. (2012). Bullying victimisation and risk of self harm in early adolescence: longitudinal cohort study. Bmj, 344, e2683. | Outcome not of interest |
| Fisher, H. L., Schreier, A., Zammit, S., Maughan, B., Munafò, M. R., Lewis, G., & Wolke, D. (2013). Pathways between childhood victimization and psychosis-like symptoms in the ALSPAC birth cohort. Schizophrenia bulletin, 39(5), 1045-1055. | Outcome not of interest |
| Fitzpatrick, K. M., Dulin, A. J., & Piko, B. F. (2007). Not just pushing and shoving: School bullying among African American adolescents. Journal of School Health, 77(1), 16-22. | Outcome not of interest |
| Floros, G. D., Siomos, K. E., Fisoun, V., Dafouli, E., & Geroukalis, D. (2013). Adolescent online cyberbullying in Greece: The impact of parental online security practices, bonding, and online impulsiveness. Journal of School Health, 83(6), 445-453. | Population not of interest |
| Floros, G., Paradeisioti, A., Hadjimarcou, M., Mappouras, D. G., Kalakouta, O., Avagianou, P., & Siomos, K. (2013). Cyberbullying in Cyprus--associated Parenting Style and Psychopathology. Studies in health technology and informatics, 191, 85-89. | No response |
| Flouri, E., & Buchanan, A. (2002). Life satisfaction in teenage boys: The moderating role of father involvement and bullying. Aggressive Behavior: Official Journal of the International Society for Research on Aggression, 28(2), 126-133. | Population not of interest |
| Flouri, E., & Buchanan, A. (2003). The role of mother involvement and father involvement in adolescent bullying behavior. Journal of Interpersonal Violence, 18(6), 634-644. | Outcome not of interest |
| Fontes, L. F. C., Conceição, O. C., & Machado, S. (2017). Childhood and adolescent sexual abuse, victim profile and its impacts on mental health. Ciencia & saude coletiva, 22, 2919-2928. | Outcome not of interest |
| Forster, M., Dyal, S. R., Baezconde-Garbanati, L., Chou, C. P., Soto, D. W., & Unger, J. B. (2013). Bullying victimization as a mediator of associations between cultural/familial variables, substance use, and depressive symptoms among Hispanic youth. Ethnicity & health, 18(4), 415-432. | No response |
| Forster, M., Grigsby, T. J., Nuñez, V., Guan, S. S. A., Rogers, C. J., & Areba, E. (2023). Bicultural Stress and Bullying Behaviors Among Immigrant Origin Youth: The Promise of Developmental Assets. Youth and Society. | Full text not available |
| Fossati, A., Borroni, S., & Maffei, C. (2012). Bullying as a style of personal relating: Personality characteristics and interpersonal aspects of self‐reports of bullying behaviours among Italian adolescent high school students. Personality and Mental Health, 6(4), 325-339. | Outcome not of interest |
| Foster, C. E., Horwitz, A., Thomas, A., Opperman, K., Gipson, P., Burnside, A., ... & King, C. A. (2017). Connectedness to family, school, peers, and community in socially vulnerable adolescents. Children and youth services review, 81, 321-331. | Outcome not of interest |
| Foster, H., & Brooks-Gunn, J. (2013). Neighborhood, family and individual influences on school physical victimization. Journal of youth and adolescence, 42(10), 1596-1610. | Outcome not of interest |
| Fousiani, K., Dimitropoulou, P., Michaelides, M. P., & Van Petegem, S. (2016). Perceived parenting and adolescent cyber-bullying: Examining the intervening role of autonomy and relatedness need satisfaction, empathic concern and recognition of humanness. Journal of child and family studies, 25(7), 2120-2129. | Outcome not of interest |
| Fredj, M. B., Bennasrallah, C., Amor, I., Trimech, F., Abroug, H., Zemni, I., ... & Sriha, A. B. (2023). Associations of psychological factors, parental involvement, and adverse health behaviors with bullying among tunisian middle school students. BMC psychology, 11(1), 154. | No response |
| Fridh, M., Lindström, M., & Rosvall, M. (2015). Subjective health complaints in adolescent victims of cyber harassment: moderation through support from parents/friends-a Swedish population-based study. BMC public health, 15(1), 1-11. | No validated measure for PF |
| Fujikawa, S., Ando, S., Nishida, A., Usami, S., Koike, S., Yamasaki, S., ... & Kasai, K. (2018). Disciplinary slapping is associated with bullying involvement regardless of warm parenting in early adolescence. Journal of adolescence, 68, 207-216. | Outcome not of interest |
| Fujikawa, S., Ando, S., Shimodera, S., Koike, S., Usami, S., Toriyama, R., ... & Nishida, A. (2016). The association of current violence from adult family members with adolescent bullying involvement and suicidal feelings. Plos one, 11(10), e0163707. | No validated measure for PF |
| Gaete, J., Tornero, B., Valenzuela, D., Rojas-Barahona, C. A., Salmivalli, C., Valenzuela, E., & Araya, R. Substance Use among Adolescents Involved in Bullying: A Cross-Sectional Multilevel Study. Psychology, 8, 1056. | No validated measure for PF |
| Galal, Y. S., Emadeldin, M., & Mwafy, M. A. (2019). Prevalence and correlates of bullying and victimization among school students in rural Egypt. Journal of the Egyptian Public Health Association, 94(1), e18-e18. | No validated measure for PF |
| Galán-Arroyo, C., Gómez-Paniagua, S., Contreras-Barraza, N., Adsuar, J. C., Olivares, P. R., & Rojo-Ramos, J. (2023, August). Bullying and self-concept, factors affecting the mental health of school adolescents. In Healthcare (Vol. 11, No. 15, p. 2214). MDPI. | Outcome not of interest |
| Galand, B., & Hospel, V. (2013). Peer victimization and school disaffection: Exploring the moderation effect of social support and the mediation effect of depression. British journal of educational psychology, 83(4), 569-590. | Outcome not of interest |
| Galindo-Domínguez, H., & Losada Iglesias, D. (2023). Bullying victimization and suicidal ideation in adolescents: The moderation effect of family, teachers and peers support. Journal of Social and Personal Relationships, 02654075231199166. | Full text not available |
| Gallet, F., & Karray, A. (2018). Le devenir des adolescents victimes de harcèlement scolaire. Psychotherapies, 38(1), 27-38. | Full text not available |
| Gálvez-Nieto, J. L., Polanco-Levicán, K., Navarro-Aburto, B., & Béltran-Véliz, J. C. (2023). Explanatory Factors of School Climate and School Identification: An Analysis of Multilevel Latent Profiles. Sustainability, 15(19), 14064. | Outcome not of interest |
| Gan, X., Li, H., Xiang, G. X., Lai, X. H., Jin, X., Wang, P. Y., & Zhu, C. S. (2022). Cumulative family risk and cyberbullying among chinese adolescents: the chain mediating role of school connectedness and cyber victimization. Frontiers in Public Health, 10, 898362. | Outcome not of interest |
| Gan, X., Qin, K. N., Xiang, G. X., & Jin, X. (2023). The relationship between parental neglect and cyberbullying perpetration among Chinese adolescent: The sequential role of cyberbullying victimization and internet gaming disorder. *Frontiers in public health*, *11*, 1128123. | Outcome not of interest |
| Garcés-Prettel, M., Santoya-Montes, Y., & Jiménez-Osorio, J. (2020). Influence of family and pedagogical communication on school violence. Comunicar, 28(63), 77-86. | Outcome not of interest |
| García-Maldonado, G., Martínez-Salazar, G. J., Saldívar-González, A. H., Sánchez-Nuncio, R., Martínez-Perales, G. M., & del Carmen Barrientos-Gómez, M. (2012). Risk factors and consequences of cyberbullying in teenagers: association with bullying. Boletín Médico del Hospital Infantil de México, 69(6), 463-474. | No validated measure for PF |
| Garisch, J. A., & Wilson, M. S. (2015). Prevalence, correlates, and prospective predictors of non-suicidal self-injury among New Zealand adolescents: cross-sectional and longitudinal survey data. Mental Health, 9, 28. | Outcome not of interest |
| Geoffroy, M. C., Arseneault, L., Girard, A., Ouellet-Morin, I., & Power, C. (2022). Association of childhood bullying victimisation with suicide deaths: findings from a 50-year nationwide cohort study. Psychological medicine, 1-8. | Outcome not of interest |
| Geoffroy, M. C., Boivin, M., Arseneault, L., Turecki, G., Vitaro, F., Brendgen, M., ... & Côté, S. M. (2016). Associations between peer victimization and suicidal ideation and suicide attempt during adolescence: results from a prospective population-based birth cohort. Journal of the American Academy of Child & Adolescent Psychiatry, 55(2), 99-105. | Outcome not of interest |
| Georgiou, S. N. (2009). Personal and maternal parameters of peer violence at school. Journal of school violence, 8(2), 100-119. | Couldn't provide correlation |
| Georgiou, S. N., & Fanti, K. A. (2010). A transactional model of bullying and victimization. Social Psychology of Education, 13(3), 295-311. | Outcome not of interest |
| Georgiou, S. N., Ioannou, M., & Stavrinides, P. (2017). Parenting styles and bullying at school: The mediating role of locus of control. *International Journal of School & Educational Psychology*, *5*(4), 226–242. | Secondary report |
| Gibson, C. L., Fagan, A. A., & Antle, K. (2014). Avoiding violent victimization among youths in urban neighborhoods: The importance of street efficacy. American journal of public health, 104(2), e154-e161. | Outcome not of interest |
| Gómez-Ortiz, O., Apolinario, C., Romera, E. M., & Ortega-Ruiz, R. (2019). The Role of Family in Bullying and Cyberbullying Involvement: Examining a new typology of parental education management based on adolescents’ view of their parents. Social Sciences, 8(1), 25. | Population not of interest |
| Gómez-Ortiz, O., Del Rey, R., Casas, J. A., & Ortega-Ruiz, R. (2014). Parenting styles and bullying involvement/Estilos parentales e implicación en bullying. Cultura y Educación, 26(1), 132-158. | No response |
| Gómez-Ortiz, O., Romera, E. M., & Ortega-Ruiz, R. (2016). Parenting styles and bullying. The mediating role of parental psychological aggression and physical punishment. Child abuse & neglect, 51, 132-143. | No population of interest |
| Gómez-Ortiz, O., Romera, E. M., Ortega-Ruiz, R., & Del Rey, R. (2018). Parenting practices as risk or preventive factors for adolescent involvement in cyberbullying: Contribution of children and parent gender. International journal of environmental research and public health, 15(12), 2664. | No population of interest |
| González, B. M., Nieto, I. D., & Mandujano, M. A. G. (2020). Student profile Not involved in bullying: description based on gender stereotypes, parenting practices, cognitive-social strategies and food over-intake. Anales de Psicología/Annals of Psychology, 36(3), 483-491. | No response |
| González-Díez, Z., Orue, I., & Calvete, E. (2017). The role of emotional maltreatment and looming cognitive style in the development of social anxiety symptoms in late adolescents. Anxiety, Stress, & Coping, 30(1), 26-38. | Population not of interest |
| Greeff, A. P., & Van den Berg, E. (2013). Resilience in families in which a child is bullied. British Journal of Guidance & Counselling, 41(5), 504-517. | Outcome not of interest |
| Guedes, M., Santos, A. J., Ribeiro, O., Freitas, M., Rubin, K. H., & Veríssimo, M. (2018). Perceived attachment security to parents and peer victimization: Does adolescent's aggressive behaviour make a difference?. Journal of adolescence, 65, 196-206. | Outcome not of interest |
| Guo, J., Li, M., Wang, X., Ma, S., & Ma, J. (2020). Being bullied and depressive symptoms in Chinese high school students: The role of social support. Psychiatry research, 284, 112676. | No response |
| Guo, S. (2021, January). A Comparison of Traditional Victims, Cyber Victims, Traditional-Cyber Victims, and Uninvolved Adolescents: A Social-Ecological Framework. In Child & Youth Care Forum (pp. 1-21). Springer US. | Outcome not of interest |
| Guo, S. (2022). Cyberbullying and delinquency in adolescence: the potential mediating effects of social attachment and delinquent peer association. Journal of interpersonal violence, 37(19-20), NP18837-NP18864. | Full text not available |
| Guo, S., Liu, J., & Wang, J. (2020). Cyberbullying Roles Among Adolescents: A Social-Ecological Theory Perspective. Journal of School Violence, 1-15. | Full text not available |
| Guo, Y., Tan, X., & Zhu, Q. J. (2022). Chains of tragedy: The impact of bullying victimization on mental health through mediating role of aggressive behavior and perceived social support. Frontiers in psychology, 13, 988003. | Outcome not of interest |
| Gur, N., Eray, S., Makinecioglu, I., Sigirli, D., & Vural, A. P. (2020). The relationship of peer bullying with familial expressed emotion and psychopathology/Akran zorbaligi ile aile duygu disa vurumu ve psikopatoloji arasindaki iliski. Anadolu Psikiyatri Dergisi, 21(1), 77-87. | Another language |
| Hagborg, J. M., Berglund, K., & Fahlke, C. (2018). Evidence for a relationship between child maltreatment and absenteeism among high-school students in Sweden. Child abuse & neglect, 75, 41-49. | Outcome not of interest |
| Hales, G., Debowska, A., Rowe, R., Boduszek, D., & Levita, L. (2023). Comparison of person-centered and cumulative risk approaches in explaining the relationship between adverse childhood experiences and behavioral and emotional problems. Journal of interpersonal violence, 08862605231153877. | Outcome not of interest |
| Hamal, M., Kekkonen, V., Kraav, S. L., Kivimäki, P., Rissanen, M. L., Hintikka, J., & Tolmunen, T. (2023). Depression and dissociation mediate the association between bullying victimization and self-cutting. Nordic journal of psychiatry, 1-10. | No validated measure for PF |
| Hamel, N., Schwab, S., & Wahl, S. (2021). Bullying: Group differences of being victim and being bully and the influence of social relations. Studies in Educational Evaluation, 68, 100964. | Outcome not of interest |
| Hamilton, J. L., Connolly, S. L., Liu, R. T., Stange, J. P., Abramson, L. Y., & Alloy, L. B. (2015). It gets better: Future orientation buffers the development of hopelessness and depressive symptoms following emotional victimization during early adolescence. Journal of abnormal child psychology, 43(3), 465-474. | Outcome not of interest |
| Hamilton, J. L., Potter, C. M., Olino, T. M., Abramson, L. Y., Heimberg, R. G., & Alloy, L. B. (2016). The temporal sequence of social anxiety and depressive symptoms following interpersonal stressors during adolescence. Journal of abnormal child psychology, 44(3), 495-509. | Outcome not of interest |
| Han, Y., Kang, H. R., Choe, J. W., & Kim, H. (2021). The moderating role of parental support in the relationship between latent profiles of bullying victimization and sense of school belonging: A cross-national comparison. Children and youth services review, 122, 105827. | No response |
| Harlow, K. C., & Roberts, R. (2010). An exploration of the relationship between social and psychological factors and being bullied. Children & Schools. | No validated measure for PF |
| Hasan, M. M., Fatima, Y., Pandey, S., Tariqujjaman, M., Cleary, A., Baxter, J., & Mamun, A. A. (2021). Pathways linking bullying victimisation and suicidal behaviours among adolescents. Psychiatry research, 302, 113992. | Outcome not of interest |
| Hasekiu, F. (2013). Risky and Protective Factors of Bullying’s Acts. Mediterranean Journal of Social Sciences, 4(10), 594-594. | Qualitative study |
| Hawkins, L. G., Brown, C. C., Goad, C., Rhynehart, A., Hemphill, T., & Snyder, H. (2021). Bullying, family cohesion, and school connectedness: a moderated-mediation multigroup analysis of adolescents. International Journal of Systemic Therapy, 32(2), 93-114. | Outcome not of interest |
| Hay, C., & Meldrum, R. (2010). Bullying victimization and adolescent self-harm: Testing hypotheses from general strain theory. Journal of youth and adolescence, 39(5), 446-459. | Population not of interest |
| Hayes, B., James, A., Barn, R., & Watling, D. (2022). Children's risk and benefit behaviours on social networking sites. Computers in Human Behavior, 130, 107147. | Outcome not of interest |
| Haynie, D. L., Nansel, T., Eitel, P., Crump, A. D., Saylor, K., Yu, K., & Simons-Morton, B. (2001). Bullies, victims, and bully/victims: Distinct groups of at-risk youth. The Journal of Early Adolescence, 21(1), 29-49. | Outcome not of interest |
| Hazemba, A., Siziya, S., Muula, A. S., & Rudatsikira, E. (2008). Prevalence and correlates of being bullied among in-school adolescents in Beijing: results from the 2003 Beijing Global School-Based Health Survey. Annals of General Psychiatry, 7(1), 1-6. | No response |
| He, D., Liu, Q. Q., & Li, X. P. (2023). Parental conflict and cyberbullying among Chinese adolescents: A moderated mediation analysis. Children and Youth Services Review, 152, 107084. | Outcome not of interest |
| He, E., Ye, X., & Zhang, W. (2023). The effect of parenting styles on adolescent bullying behaviours in China: The mechanism of interpersonal intelligence and intrapersonal intelligence. Heliyon, 9(4). | Outcome not of interest |
| Healy, K. L., & Sanders, M. R. (2017). Antecedents of treatment resistant depression in children victimized by peers. Child Psychiatry & Human Development, 48(1), 107-119. | Outcome not of interest |
| Healy, K. L., & Sanders, M. R. (2018). Mechanisms through which supportive relationships with parents and peers mitigate victimization, depression and internalizing problems in children bullied by peers. Child Psychiatry & Human Development, 49(5), 800-813. | Outcome not of interest |
| Healy, K. L., Grzazek, O. Y., & Sanders, M. R. (2020). Attributions for improvement in children bullied at school. Journal of school violence, 19(2), 219-233. | Outcome not of interest |
| Hébert, M., Cénat, J. M., Blais, M., Lavoie, F., & Guerrier, M. (2016). Child sexual abuse, bullying, cyberbullying, and mental health problems among high schools students: a moderated mediated model. Depression and anxiety, 33(7), 623-629. | Outcome not of interest |
| Hebron, J., & Humphrey, N. (2014). Exposure to bullying among students with autism spectrum conditions: A multi-informant analysis of risk and protective factors. Autism, 18(6), 618-630. | Outcome not of interest |
| Heino, E., Ellonen, N., & Kaltiala, R. (2020). Transgender identity is associated with bullying involvement among Finnish adolescents. Frontiers in psychology, 11. | No validated measure for PF |
| Helfrich, E. L., Doty, J. L., Su, Y. W., Yourell, J. L., & Gabrielli, J. (2020). Parental views on preventing and minimizing negative effects of cyberbullying. Children and Youth Services Review, 118, 105377. | Population not of interest |
| Hemphill, S. A., & Heerde, J. A. (2014). Adolescent predictors of young adult cyberbullying perpetration and victimization among Australian youth. Journal of Adolescent Health, 55(4), 580-587. | Outcome not of interest |
| Hemphill, S. A., Kotevski, A., Tollit, M., Smith, R., Herrenkohl, T. I., Toumbourou, J. W., & Catalano, R. F. (2012). Longitudinal predictors of cyber and traditional bullying perpetration in Australian secondary school students. Journal of Adolescent Health, 51(1), 59-65. | Outcome not of interest |
| Hemphill, S. A., Tollit, M., & Herrenkohl, T. I. (2014). Protective factors against the impact of school bullying perpetration and victimization on young adult externalizing and internalizing problems. Journal of school violence, 13(1), 125-145. | Outcome not of interest |
| Hemphill, S. A., Tollit, M., Kotevski, A., & Heerde, J. A. (2015). Predictors of traditional and cyber-bullying victimization: A longitudinal study of Australian secondary school students. Journal of interpersonal violence, 30(15), 2567-2590. | Outcome not of interest |
| Herba, C. M., Ferdinand, R. F., Stijnen, T., Veenstra, R., Oldehinkel, A. J., Ormel, J., & Verhulst, F. C. (2008). Victimisation and suicide ideation in the TRAILS study: specific vulnerabilities of victims. Journal of Child Psychology and Psychiatry, 49(8), 867-876. | No response |
| Hernández Carrillo, M., & Gutiérrez Martínez, M. I. (2013). Risk Factors Associated With School Bullying in Local Authority Schools in Four Municipalities of Valle del Cauca, Colombia. Year 2009. Revista colombiana de psiquiatria, 42(3), 238-247. | Another language |
| Hernandez de Frutos, T. (2014). Factors of risk and protection/resilience in adolescent scholar bullying: longitudinal analysis. Revista Internacional de Sociología (RIS), vol. 72 (3)(2014), págs. 583-608. | No validated measure for PF |
| Hernandez de Frutos, T. (2014). Factors of risk and protection/resilience in adolescent scholar bullying: longitudinal analysis. Revista Internacional de Sociología (RIS), vol. 72 (3)(2014), págs. 583-608. | Population not of interest |
| Hernández-Vásqueza, A., Vargas-Fernándezb, R., Díaz-Seijasc, D., Tapia-Lópeze, E., & Bendezu-Quispef, G. (2019). Prevalence of suicidal behaviors and associated factors among Peruvian adolescent students. | Outcome not of interest |
| Herrero, J., Estévez, E., & Musitu, G. (2006). The relationships of adolescent school-related deviant behaviour and victimization with psychological distress: Testing a general model of the mediational role of parents and teachers across groups of gender and age. Journal of adolescence, 29(5), 671-690. | Outcome not of interest |
| Higuita-Gutiérrez, L. F., & Cardona-Arias, J. A. (2017). Variables of the family, school, and social environment context that determine bullying in adolescents in Medellin, Colombia, 2014. Journal of school violence, 16(1), 68-85. | Population not of interest |
| Himmelstein, M. S., & Puhl, R. M. (2019). Weight‐based victimization from friends and family: implications for how adolescents cope with weight stigma. Pediatric obesity, 14(1), e12453. | No validated measure for PF |
| Hinduja, S., & Patchin, J. W. (2022). Bullying and cyberbullying offending among US youth: The influence of six parenting dimensions. Journal of Child and Family Studies, 31(5), 1454-1473. | Outcome not of interest |
| Ho, H. Y., Chen, Y. L., & Yen, C. F. (2022). Moderating effects of friendship and family support on the association between bullying victimization and perpetration in adolescents. Journal of interpersonal violence, 37(7-8), NP4640-NP4659. | No response |
| Ho, H. Y., Chen, Y. L., & Yen, C. F. Moderating Effects of Friendship and Family Support on the Association Between Bullying Victimization and Perpetration in Adolescents. Journal of Interpersonal Violence, 0886260520985503. | No response |
| Hogye, S. I., Jansen, P. W., Lucassen, N., & Keizer, R. (2022). The relation between harsh parenting and bullying involvement and the moderating role of child inhibitory control: A population‐based study. Aggressive behavior, 48(2), 141-151. | Outcome not of interest |
| Holt, M. K., & Espelage, D. L. (2007). Perceived social support among bullies, victims, and bully-victims. Journal of Youth and Adolescence, 36(8), 984-994. | Population not of interest |
| Holt, M. K., Finkelhor, D., & Kantor, G. K. (2007). Hidden forms of victimization in elementary students involved in bullying. School Psychology Review, 36(3), 345-360. | Outcome not of interest |
| Holt, M. K., Kaufman Kantor, G., & Finkelhor, D. (2008). Parent/child concordance about bullying involvement and family characteristics related to bullying and peer victimization. Journal of School Violence, 8(1), 42-63. | Outcome not of interest |
| Hong, J. S., Choi, J., Espelage, D. L., Wu, C. F., Boraggina-Ballard, L., & Fisher, B. W. (2020, November). Are Children of Welfare Recipients at a Heightened Risk of Bullying and Peer Victimization?. In Child & Youth Care Forum (pp. 1-22). Springer US. | No validated measure for PF |
| Hong, J. S., Choi, J., Espelage, D. L., Wu, C. F., Boraggina-Ballard, L., & Fisher, B. W. (2021, June). Are children of welfare recipients at a heightened risk of bullying and peer victimization?. In Child & Youth Care Forum (Vol. 50, pp. 547-568). Springer US. | No validated measure for PF |
| Hong, J. S., Choi, M. J., Kim, I., Butler-Barnes, S., Kruman Mountain, S., & Voisin, D. R. (2021). Identifying protective factors in the association between peer victimization and internalizing symptoms of African American adolescents in four Chicago’s southside neighborhoods. School mental health, 1-14. | No validated measure for PF |
| Hong, J. S., Hunter, S. C., Kim, J., Piquero, A. R., & Narvey, C. (2021). Racial differences in the applicability of Bronfenbrenner’s ecological model for adolescent bullying involvement. Deviant Behavior, 42(3), 404-424. | No response |
| Hong, J. S., Hunter, S. C., Kim, J., Piquero, A. R., & Narvey, C. (2021). Racial differences in the applicability of Bronfenbrenner’s ecological model for adolescent bullying involvement. Deviant Behavior, 42(3), 404-424. | No response |
| Hong, J. S., Kim, D. H., & Hunter, S. C. (2019). Applying the social–ecological framework to explore bully–victim subgroups in South Korean schools. Psychology of violence, 9(3), 267. | Outcome not of interest |
| Hong, J. S., Kim, D. H., & Piquero, A. R. (2017). Assessing the links between punitive parenting, peer deviance, social isolation and bullying perpetration and victimization in South Korean adolescents. Child abuse & neglect, 73, 63-70. | Outcome not of interest |
| Hong, J. S., Kim, D. H., Allen, J. L., Okumu, M., Lee, J. J., & Voisin, D. R. (2021). Bullying victimization and STIs: Parental communication and parental sexual communication as moderators. Research on Social Work Practice, 31(7), 706-715. | Full text not available |
| Hong, J. S., Kim, D. H., deLara, E. W., Wei, H. S., Prisner, A., & Alexander, N. B. (2021). Parenting style and bullying and victimization: Comparing foreign-born Asian, US-born Asian, and White American adolescents. Journal of family violence, 36, 799-811. | No response |
| Hong, J. S., Kim, D. H., Narvey, C., Piquero, A. R., deLara, E., & Padilla, Y. C. (2020). Understanding the link between family economic hardship and children’s bullying behavior. Youth & Society, 0044118X20932594. | Outcome not of interest |
| Hong, J. S., Kim, D. H., Thornberg, R., Kang, J. H., & Morgan, J. T. (2018). Correlates of direct and indirect forms of cyberbullying victimization involving South Korean adolescents: An ecological perspective. Computers in Human Behavior, 87, 327-336. | Population not of interest |
| Hong, J. S., Lee, J. M., Rivas-Koehl, M. M., Butler-Barnes, S., de Lara, E. W., & Voisin, D. R. (2023). Correlates of Perceived School Safety Among Black Adolescents in Chicago: Are There Sexual Orientation Differences?. Clinical Social Work Journal, 51(1), 86-99. | Population not of interest |
| Hong, J. S., Lee, J., McCloskey, L. A., Victor, B. G., Wei, H. S., & Voisin, D. R. (2021). Pathways from witnessing parental intimate partner violence to involvement in bullying: empirically testing a proposed conceptual framework. The journal of primary prevention, 42, 583-602. | Outcome not of interest |
| Hong, J. S., Lee, J., Thornberg, R., Peguero, A. A., Washington, T., & Voisin, D. R. (2020). Social-ecological pathways to school motivation and future orientation of African American adolescents in Chicago. The Journal of Educational Research, 113(5), 384-395. | Population not of interest |
| Hong, J. S., Ryou, B., Wei, H. S., Allen-Meares, P., & Espelage, D. L. (2019). Identifying protective factors that potentially buffer the association between peer victimization and weapon-carrying behavior among US adolescents. School psychology international, 40(4), 381-402. | Population not of interest |
| Hong, J. S., Song, E. J., Peguero, A. A., Wu, C. F., & Cameron Schmaeman, A. (2020). Can Family and Neighborhood Cohesiveness Buffer the Association Between Family Economic Hardship and Children’s Peer Victimization?. Families in Society, 101(3), 382-394. | No validated measure for PF |
| Hong, J. S., Valido, A., Espelage, D. L., Lee, S. J., DeLara, E. W., & Lee, J. M. (2021). Adolescent bullying victimization and psychosomatic symptoms: Can relationship quality with fathers buffer this association?. Journal of affective disorders, 295, 1387-1397. | No response |
| Hong, J. S., Valido, A., Rivas‐Koehl, M. M., Wade, R. M., Espelage, D. L., & Voisin, D. R. (2021). Bullying victimization, psychosocial functioning, and protective factors: Comparing African American heterosexual and sexual minority adolescents in Chicago's Southside. Journal of community psychology. | Population not of interest |
| Hong, J. S., Williams-Butler, A. B., Garthe, R. C., Kim, J., & Voisin, D. R. (2020, April). Relationship Between Coping Strategies and Peer Victimization Among Low-Income African American Youth Living in Chicago. In Child & Youth Care Forum (Vol. 49, No. 2, pp. 287-302). Springer US. | Population not of interest |
| Hong, J. S., Yan, Y., Clark Goings, T. T., Takahashi, L. M., Tabb, K. M., & Voisin, D. R. (2022). Potential pathways from peer victimization to alcohol use among biracial adolescents: the intervening role of parental support. Journal of school health, 92(8), 786-793. | Full text not available |
| Hong, J. S., Yan, Y., Espelage, D. L., Tabb, K. M., Caravita, S. C., & Voisin, D. R. (2022). Peer victimization and adverse psychosocial wellbeing of Black/White biracial adolescents: Is ease of talking with family a protective buffer?. School Psychology Review, 1-14. | Full text not available |
| Hong, J. S., Yan, Y., Gonzalez‐Prendes, A. A., Espelage, D. L., & Allen‐Meares, P. (2021). Correlates of school bullying victimization among Black/White biracial adolescents: Are they similar to their monoracial Black and White peers?. Psychology in the Schools, 58(3), 601-621. | No response |
| Hong, J. S., Zhang, S., Espelage, D. L., & Allen-Meares, P. (2023). Dimensions of Parenting and Children’s Bullying Victimization: A Look at the Racial/Ethnic and Grade Level Differences. The Journal of Genetic Psychology, 1-15. | Full text not available |
| Hong, J. S., Zhang, S., Gonzalez-Prendes, A. A., & Albdour, M. (2020). Exploring whether talking with parents, siblings, and friends moderates the association between peer victimization and adverse psychosocial outcomes. Journal of interpersonal violence, 0886260519898432. | No response |
| Hong, J. S., Zhang, S., Wright, M. F., & Wachs, S. (2023). Racial and ethnic differences in the antecedents of cyberbullying victimization in early adolescence: an ecological systems framework. The Journal of Early Adolescence, 43(1), 59-89. | No response |
| Hood, M., & Duffy, A. L. (2018). Understanding the relationship between cyber-victimisation and cyber-bullying on Social Network Sites: The role of moderating factors. Personality and Individual Differences, 133, 103-108. | Population not of interest |
| Horwitz, A. G., Grupp-Phelan, J., Brent, D., Barney, B. J., Casper, T. C., Berona, J., ... & Pediatric Emergency Care Applied Research Network. (2021). Risk and protective factors for suicide among sexual minority youth seeking emergency medical services. Journal of Affective Disorders, 279, 274-281. | Outcome not of interest |
| Horwitza, A. G., Grupp-Phelanb, J., Brentc, D., Barneyd, B. J., Charles, T., Casperd, J. B., ... & Cheryl, A. (2021). Risk and Protective Factors for Suicide among Sexual Minority Youth seeking Emergency Medical Services. J Affect Disord, 279, 274-281. | Outcome not of interest |
| Hu, B. (2021). Is bullying victimization in childhood associated with mental health in old age. Journals of Gerontology-Series B Psychological Sciences and Social Sciences, 76(1), 161-172. | Population not of interest |
| Hu, Q., Bernardo, A. B., Lam, S. W., & Cheang, P. K. (2018). Individualism-collectivism orientations and coping styles of cyberbullying victims in Chinese culture. Current Psychology, 37(1), 65-72. | Population not of interest |
| Huang, C. L., Alimu, Y., & Yang, S. C. (2023). Factors influencing ethnic minority students' helping behavior in cyberbullying: perceived severity of cyberbullying from various perspectives, the online disinhibition effect, and parental online discipline style. European Journal of Psychology of Education, 1-23. | Population not of interest |
| Huang, H. W., Chen, J. L., & Wang, R. H. (2018). Factors associated with peer victimization among adolescents in Taiwan. Journal of Nursing Research, 26(1), 52-59. | Outcome not of interest |
| Huang, L., Chen, Y., Zhu, J., & Zhang, W. (2022). Association of paternal rejection with externalizing problems of adolescents: A moderated mediation model. PsyCh Journal, 11(4), 470-480. | Full text not available |
| Husky, M. M., Sadikova, E., Lee, S., Alonso, J., Auerbach, R. P., Bantjes, J., ... & Kessler, R. C. (2023). Childhood adversities and mental disorders in first-year college students: results from the World Mental Health International College Student Initiative. Psychological Medicine, 53(7), 2963-2973. | Population not of interest |
| Iffland, B., Sansen, L. M., Catani, C., & Neuner, F. (2012). Emotional but not physical maltreatment is independently related to psychopathology in subjects with various degrees of social anxiety: a web-based internet survey. BMC psychiatry, 12(1), 1-8. | Population not of interest |
| Ighaede-Edwards, I. G., Liu, X., Olawade, D. B., Ling, J., Odetayo, A., & David-Olawade, A. C. (2023). Prevalence and predictors of bullying among in-school adolescents in Nigeria. Journal of Taibah University Medical Sciences, 18(6), 1329. | No validated measure for PF |
| Ingram, K. M., Espelage, D. L., Davis, J. P., & Merrin, G. J. (2020). Family violence, sibling, and peer aggression during adolescence: associations with behavioral health outcomes. Frontiers in psychiatry, 11, 26. | Outcome not of interest |
| Iñiguez-Berrozpe, T., Orejudo-Hernández, S., Ruiz-Eugenio, L., & Elboj-Saso, C. (2021). School networks of positive relationships, attitudes against violence, and prevention of relational bullying in victim, bystander, and aggressor agents. Journal of School Violence, 1-16. | Outcome not of interest |
| Innamorati, M., Parolin, L., Tagini, A., Santona, A., Bosco, A., De Carli, P., ... & Sarracino, D. (2018). Attachment, social value orientation, sensation seeking, and bullying in early adolescence. Frontiers in psychology, 9, 239. | No response |
| Ioannidou, L., & Zafiropoulou, M. (2021). Parenting practices and internalising symptoms in children: the role of victimisation and behavioural inhibition through complex moderated-mediation models. Emotional and Behavioural Difficulties, 26(3), 280-292. | Secondary report |
| Iorga, M., Pop, L. M., Croitoru, I., Hanganu, E., & Anton-Păduraru, D. T. (2022). Exploring the importance of gender, family affluence, parenting style and loneliness in cyberbullying victimization and aggression among Romanian adolescents. Behavioral Sciences, 12(11), 457. | Population not of interest |
| Iotti, N. O., Menin, D., Longobardi, C., & Jungert, T. (2023). Investigating the effects of autonomy-supportive parenting practices on Italian young adolescent students’ motivation to defend victims of bullying: findings on the mediating roles of reactance, depression, anxiety, and stress. Frontiers in psychology, 14, 1156807. | Outcome not of interest |
| Islam, M. I., Yunus, F. M., Kabir, E., & Khanam, R. (2022). Evaluating Risk and Protective Factors for Suicidality and Self-Harm in Australian Adolescents With Traditional Bullying and Cyberbullying Victimizations. American journal of health promotion: AJHP, 36(1), 73-83. | No validated measure for PF |
| Ismail, S., Odland, M. L., Malik, A., Weldegiorgis, M., Newbigging, K., Peden, M., ... & Davies, J. (2021). The relationship between psychosocial circumstances and injuries in adolescents. | Outcome not of interest |
| Itani, T., Jacobsen, K. H., & Kraemer, A. (2017). Suicidal ideation and planning among Palestinian middle school students living in Gaza Strip, West Bank, and United Nations Relief and Works Agency (UNRWA) camps. International journal of pediatrics and adolescent medicine, 4(2), 54-60. | No validated measure for PF |
| Iwanaga, M., Nishi, D., Obikane, E., & Kawakami, N. (2023). Age of victimization and moderating role of social support for the relationship between school-age bullying and life satisfaction in middle-age. Scandinavian journal of public health, 14034948221148788. | Full text not available |
| Jadambaa, A., Graves, N., Cross, D., Pacella, R., Thomas, H. J., Scott, J. G., ... & Brain, D. (2022). Economic evaluation of an intervention designed to reduce bullying in Australian schools. Applied health economics and health policy, 20, 79-89. | Outcome not of interest |
| Jankauskiene, R., Kardelis, K., Sukys, S., & Kardeliene, L. (2008). Associations between school bullying and psychosocial factors. Social Behavior and Personality: an international journal, 36(2), 145-162. | No validated measure for PF |
| Jantzer, V., Haffner, J., Parzer, P., Resch, F., & Kaess, M. (2015). Does parental monitoring moderate the relationship between bullying and adolescent nonsuicidal self-injury and suicidal behavior? A community-based self-report study of adolescents in Germany. BMC public health, 15(1), 1-8. | No validated measure for PF |
| Jaskulska, S., Jankowiak, B., Pérez-Martínez, V., Pyżalski, J., Sanz-Barbero, B., Bowes, N., ... & Vives-Cases, C. (2022). Bullying and Cyberbullying Victimization and Associated Factors among Adolescents in Six European Countries. Sustainability, 14(21), 14063. | Outcome not of interest |
| Jatrana, S., Hasan, M. M., Mamun, A. A., & Fatima, Y. (2021). Global variation in hand hygiene practices among adolescents: the role of family and school-level factors. International journal of environmental research and public health, 18(9), 4984. | Outcome not of interest |
| Jattamart, A., & Kwangsawad, A. (2021). What awareness variables are associated with motivation for changing risky behaviors to prevent recurring victims of cyberbullying?. Heliyon, 7(10). | Outcome not of interest |
| Jeong, S., Davis, J., & Han, Y. (2015). Who becomes more violent among Korean adolescents? Consequences of victimisation in school. Criminal behaviour and mental health, 25(2), 141-155. | No validated measure for PF |
| Joronen, K., Konu, A., Rankin, H. S., & Åstedt-Kurki, P. (2012). An evaluation of a drama program to enhance social relationships and anti-bullying at elementary school: a controlled study. Health promotion international, 27(1), 5-14. | Outcome not of interest |
| Ju, H. J., & Lee, S. H. (2019). Mothers’ Perceptions of the Phenomenon of Bullying among Young Children in South Korea. Social Sciences, 8(1), 12. | Outcome not of interest |
| Jun, W. (2020). A study on the cause analysis of cyberbullying in Korean adolescents. International journal of environmental research and public health, 17(13), 4648. | No validated measure for PF |
| Kadiroğlu, T., Hendekci, A., & Tosun, Ö. (2018). Investigation of the relationship between peer victimization and quality of life in school-age adolescents. Archives of psychiatric nursing, 32(6), 850-854. | Outcome not of interest |
| Karakus, M., Courtney, M., & Aydin, H. (2023). Understanding the academic achievement of the first-and second-generation immigrant students: A multi-level analysis of PISA 2018 data. Educational assessment, evaluation and accountability, 35(2), 233-278. | No response |
| Karakus, O., Altinel, B., & Kocak Uyaroglu, A. (2023). The relationship between peer bullying, loneliness, and social support in refugee adolescents. Journal of child and adolescent psychiatric nursing. | Full text not available |
| Karlsson, E., Stickley, A., Lindblad, F., Schwab-Stone, M., & Ruchkin, V. (2014). Risk and protective factors for peer victimization: a 1-year follow-up study of urban American students. European child & adolescent psychiatry, 23(9), 773-781. | No validated measure for PF |
| Katzer, C., Fetchenhauer, D., & Belschak, F. (2009). Cyberbullying: Who are the victims? A comparison of victimization in Internet chatrooms and victimization in school. Journal of Media Psychology, 21(1), 25-36. | No validated measure for PF |
| Kaufman, T. M., & Baams, L. (2022). Disparities in perpetrators, locations, and reports of victimization for sexual and gender minority adolescents. Journal of Adolescent Health, 70(1), 99-107. | No validated measure for PF |
| Kaufman, T. M., Kretschmer, T., Huitsing, G., & Veenstra, R. (2018). Why does a universal anti-bullying program not help all children? Explaining persistent victimization during an intervention. Prevention Science, 19(6), 822-832. | Outcome not of interest |
| Kaufman, T. M., Kretschmer, T., Huitsing, G., & Veenstra, R. (2020). Caught in a vicious cycle? Explaining bidirectional spillover between parent-child relationships and peer victimization. Development and psychopathology, 32(1), 11-20. | Outcome not of interest |
| Keelan, C. M., Schenk, A. M., McNally, M. R., & Fremouw, W. J. (2014). The interpersonal worlds of bullies: Parents, peers, and partners. Journal of interpersonal violence, 29(7), 1338-1353. | Population not of interest |
| Kehusmaa, J., Ruotsalainen, H., Männikkö, N., Alakokkare, A. E., Niemelä, M., Jääskeläinen, E., & Miettunen, J. (2022). The association between the social environment of childhood and adolescence and depression in young adulthood-A prospective cohort study. Journal of affective disorders, 305, 37-46. | Outcome not of interest |
| Kelleher, I., Harley, M., Lynch, F., Arseneault, L., Fitzpatrick, C., & Cannon, M. (2008). Associations between childhood trauma, bullying and psychotic symptoms among a school-based adolescent sample. The British Journal of Psychiatry, 193(5), 378-382. | Outcome not of interest |
| Kelly, A. B., Mason, W. A., Chmelka, M. B., Herrenkohl, T. I., Kim, M. J., Patton, G. C., ... & Catalano, R. F. (2016). Depressed mood during early to middle adolescence: A bi-national longitudinal study of the unique impact of family conflict. Journal of youth and adolescence, 45(8), 1604-1613. | No validated measure for PF |
| Kenny, K. S., Merry, L., Brownbridge, D. A., & Urquia, M. L. (2020). Factors associated with cyber-victimization among immigrants and non-immigrants in Canada: a cross-sectional nationally-representative study. BMC public health, 20(1), 1-13. | Population not of interest |
| Kerr, D. C., Gini, G., Owen, L. D., & Capaldi, D. M. (2018). Peer teasing experiences of fathers and their children: Intergenerational associations and transmission mechanisms. Child abuse & neglect, 86, 33-44. | Outcome not of interest |
| Khamis, V. (2015). Bullying among school-age children in the greater Beirut area: Risk and protective factors. Child abuse & neglect, 39, 137-146. | No response |
| Khetarpal, S. K., Szoko, N., Culyba, A. J., Shaw, D., & Ragavan, M. I. (2022). Associations between parental monitoring and multiple types of youth violence victimization: a brief report. Journal of interpersonal violence, 37(19-20), NP19216-NP19227. | No response |
| Khurana, A., Bleakley, A., Jordan, A. B., & Romer, D. (2015). The protective effects of parental monitoring and internet restriction on adolescents’ risk of online harassment. Journal of youth and Adolescence, 44(5), 1039-1047. | Outcome not of interest |
| Kim, D., & Lee, C. S. (2023). Cyberbullying Victimization and Perpetration in South Korean Youth: Structural Equation Modeling and Latent Means Analysis. Crime & Delinquency, 00111287231193992. | Full text not available |
| Kim, J. H. (2021). Factors associated with smartphone addiction tendency in Korean adolescents. International Journal of Environmental Research and Public Health, 18(21), 11668. | Outcome not of interest |
| Kim, J. H., Hahlweg, K., & Schulz, W. (2021). Early childhood parenting and adolescent bullying behavior: Evidence from a randomized intervention at ten-year follow-up. Social Science & Medicine, 282, 114114. | No validated measure for PF |
| Kim, J., & Ko, Y. (2021). Influence of experiencing bullying victimization on suicidal ideation and behaviors in Korean adolescents. International journal of environmental research and public health, 18(20), 10853. | No validated measure for PF |
| Kim, J., Lee, Y., Leban, L., & Jennings, W. G. (2023). Association between school bullying victimization and sexual risk-taking among South Korean adolescents: the role of teacher and parental relationships. Archives of sexual behavior, 1-11. | No validated measure for PF |
| Kim, S. (2022). The Role of Social Capital in the Onset of Victimization against Children. Child Indicators Research, 15(1), 67-86. | Outcome not of interest |
| Kim, S. S., Craig, W. M., King, N., Bilz, L., Cosma, A., Molcho, M., ... & Pickett, W. (2022). Bullying, mental health, and the moderating role of supportive adults: a cross-national analysis of adolescents in 45 countries. International journal of public health, 67, 1604264. | Outcome not of interest |
| Klinck, M., Vannucci, A., Fagle, T., & Ohannessian, C. M. (2020). Appearance-related teasing and substance use during early adolescence. Psychology of addictive behaviors. | Outcome not of interest |
| Klomek, A. B. (2020). Peer and parents’ support are crucial protective factors against adolescent victimization by bullying. EClinicalMedicine, 22. | Qualitative study |
| Klomek, A. B., Snir, A., Apter, A., Carli, V., Wasserman, C., Hadlaczky, G., ... & Wasserman, D. (2016). Association between victimization by bullying and direct self injurious behavior among adolescence in Europe: a ten-country study. European child & adolescent psychiatry, 25(11), 1183-1193. | No validated measure for PF |
| Knous-Westfall, H. M., Ehrensaft, M. K., MacDonell, K. W., & Cohen, P. (2012). Parental intimate partner violence, parenting practices, and adolescent peer bullying: A prospective study. Journal of child and Family studies, 21(5), 754-766. | Outcome not of interest |
| Konac, D., Young, K. S., Lau, J., & Barker, E. D. (2021). Comorbidity between depression and anxiety in adolescents: Bridge symptoms and relevance of risk and protective factors. Journal of psychopathology and behavioral assessment, 43, 583-596. | Outcome not of interest |
| Konishi, C., & Hymel, S. (2009). Bullying and stress in early adolescence: The role of coping and social support. The Journal of Early Adolescence, 29(3), 333-356. | Outcome not of interest |
| Konishi, C., & Saewyc, E. (2014). Still a target: Sexual diversity and power of caring. School Psychology International, 35(5), 504-515. | No validated measure for PF |
| Kouwenberg, M., Rieffe, C., Theunissen, S. C., & de Rooij, M. (2012). Peer victimization experienced by children and adolescents who are deaf or hard of hearing. PLoS One, 7(12), e52174. | No validated measure for PF |
| Kretschmer, T., Sentse, M., Meeus, W., Verhulst, F. C., Veenstra, R., & Oldehinkel, A. J. (2016). Configurations of adolescents' peer experiences: Associations with parent–child relationship quality and parental problem behavior. Journal of Research on Adolescence, 26(3), 474-491. | Outcome not of interest |
| Kretschmer, T., Veenstra, R., Branje, S., Reijneveld, S. A., Meeus, W. H., Deković, M., ... & Oldehinkel, A. J. (2018). How competent are adolescent bullying perpetrators and victims in mastering normative developmental tasks in early adulthood?. Journal of abnormal child psychology, 46(1), 41-56. | Outcome not of interest |
| Ladd, G. W., & Ladd, B. K. (1998). Parenting behaviors and parent-child relationships: correlates of peer victimization in kindergarten?. Developmental Psychology, 34(6), 1450-1458. | Outcome not of interest |
| Lahav-Kadmiel, Z., & Brunstein-Klomek, A. (2018). Bullying victimization and depressive symptoms in adolescence: The moderating role of parent-child conflicts among boys and girls. Journal of adolescence, 68, 152-158. | Outcome not of interest |
| Lan, M., Pan, Q., Tan, C. Y., & Law, N. W. Y. (2022). Understanding protective and risk factors affecting adolescents’ well-being during the COVID-19 pandemic. npj Science of Learning, 7(1), 32. | Outcome not of interest |
| Lardier Jr, D. T., Bermea, A. M., Pinto, S. A., Garcia-Reid, P., & Reid, R. J. (2017). The relationship between sexual minority status and suicidal ideations among urban Hispanic adolescents. Journal of LGBT issues in counseling, 11(3), 174-189. | Outcome not of interest |
| Lardier, D. T., Pinto, S. A., Brammer, M. K., Garcia-Reid, P., & Reid, R. J. (2020). The Relationship Between Queer Identity, Social Connection, School Bullying, and Suicidal Ideations Among Youth of Color. Journal of LGBT Issues in Counseling, 14(2), 74-99. | No response |
| Larrañaga, E., Yubero, S., & Navarro, R. (2018). Parents’ responses to coping with bullying: Variations by adolescents’ self-reported victimization and parents’ awareness of bullying involvement. Social Sciences, 7(8), 121. | No validated measure for PF |
| Latham, R. M., Meehan, A. J., Arseneault, L., Stahl, D., Danese, A., & Fisher, H. L. (2019). Development of an individualized risk calculator for poor functioning in young people victimized during childhood: A longitudinal cohort study. Child abuse & neglect, 98, 104188. | Outcome not of interest |
| Lätsch, D. C., Nett, J. C., & Hümbelin, O. (2017). Poly-victimization and its relationship with emotional and social adjustment in adolescence: Evidence from a national survey in Switzerland. Psychology of violence, 7(1), 1. | Outcome not of interest |
| Lawson, M. A., Alameda-Lawson, T., Downer, J., & Anderson, E. (2013). Analyzing sub-population profiles and risk factors for school bullying. Children and Youth Services Review, 35(6), 973-983. | No validated measure for PF |
| Le, H. T. H., Dunne, M. P., Campbell, M. A., Gatton, M. L., Nguyen, H. T., & Tran, N. T. (2017). Temporal patterns and predictors of bullying roles among adolescents in Vietnam: a school-based cohort study. Psychology, health & medicine, 22(sup1), 107-121. | No response |
| Le, L. K. D., Chatterton, M. L., Rapee, R. M., Fitzpatrick, S., Bussey, K., Hudson, J., ... & Mihalopoulos, C. (2023). Burden and preference-based quality of life associated with bullying in children. European Child & Adolescent Psychiatry, 32(1), 53-62. | Outcome not of interest |
| Leadbeater, B., & Hoglund, W. (2006). Changing the social contexts of peer victimization. Journal of the Canadian Academy of Child and Adolescent Psychiatry, 15(1), 21. | Outcome not of interest |
| Leadbeater, B., Sukhawathanakul, P., Smith, D., & Bowen, F. (2015). Reciprocal associations between interpersonal and values dimensions of school climate and peer victimization in elementary school children. Journal of Clinical Child & Adolescent Psychology, 44(3), 480-493. | No validated measure for PF |
| Lebrun-Harris, L. A., Sherman, L. J., Limber, S. P., Miller, B. D., & Edgerton, E. A. (2019). Bullying victimization and perpetration among US children and adolescents: 2016 National Survey of Children’s Health. Journal of Child and Family Studies, 28(9), 2543-2557. | No validated measure for PF |
| Ledwell, M., & King, V. (2015). Bullying and internalizing problems: Gender differences and the buffering role of parental communication. Journal of family issues, 36(5), 543-566. | No validated measure for PF |
| Lee, C. H. (2011). An ecological systems approach to bullying behaviors among middle school students in the United States. Journal of interpersonal violence, 26(8), 1664-1693. | No validated measure for PF |
| Lee, C. H., & Song, J. (2012). Functions of parental involvement and effects of school climate on bullying behaviors among South Korean middle school students. Journal of interpersonal violence, 27(12), 2437-2464. | Outcome not of interest |
| Lee, J. M., Hong, J. S., Resko, S. M., Gonzalez-Prendes, A. A., & Voisin, D. R. (2021). Ecological correlates of bullying and peer victimization among urban African American adolescents. The Journal of Educational Research, 114(4), 346-356. | Population not of interest |
| Lee, J., Cheung, H. S., Chee, G., & Chai, V. E. (2021). The moderating roles of empathy and attachment on the association between latent class typologies of bullying involvement and depressive and anxiety symptoms in Singapore. School mental health, 1-17. | Population not of interest |
| Lee, J., Choi, M. J., Thornberg, R., & Hong, J. S. (2020). Exploring Sex Differences in the Association between Bullying Involvement and Alcohol and Marijuana Use among US Adolescents in 6th to 10th Grade. Substance use & misuse, 55(8), 1203-1213. | No validated measure for PF |
| Lee, J., Chun, J., Kim, J., Lee, J., & Lee, S. (2021). A social-ecological approach to understanding the relationship between cyberbullying victimization and suicidal ideation in South Korean adolescents: The moderating effect of school connectedness. International Journal of Environmental Research and Public Health, 18(20), 10623. | Population not of interest |
| Lee, J., Hong, J. S., Resko, S. M., & Tripodi, S. J. (2018). Face-to-face bullying, cyberbullying, and multiple forms of substance use among school-age adolescents in the USA. School mental health, 10(1), 12-25. | No validated measure for PF |
| Lee, J., Roh, B. R., & Yang, K. E. (2022). Exploring the association between social support and patterns of bullying victimization among school-aged adolescents. Children and youth services review, 136, 106418. | Population not of interest |
| Lee, N., Ray, J. V., & Peck, J. H. (2023). Examining the mediating role of peer antisocial behavior on the relationship between parenting and bullying behaviors. Journal of Crime and Justice, 46(3), 348-368. | Population not of interest |
| Lee, S., & Chun, J. (2020). Conceptualizing the impacts of cyberbullying victimization among Korean male adolescents. Children and Youth Services Review, 117, 105275. | Outcome not of interest |
| Lee, S., & Mun, I. B. (2022). How does perceived parental rejection influence cyberbullying by children? A serial mediation model of children’s depression and smartphone addiction. The Social Science Journal, 1-16. | Full text not available |
| Lee, Y., & Kim, S. (2017). The role of anger and depressive mood in transformation process from victimization to perpetration. Child abuse & neglect, 63, 131-140. | No validated measure for PF |
| Leff, S. S., Paskewich, B. S., & Blum, N. J. (2020). Exploring early childhood factors as an avenue to address chronic peer victimization. Pediatrics, 145(5). | Outcome not of interest |
| Lemstra, M., Rogers, M., Redgate, L., Garner, M., & Moraros, J. (2011). Prevalence, risk indicators and outcomes of bullying among on-reserve first nations youth. Canadian journal of public health, 102(6), 462-466. | No validated measure for PF |
| Lereya, S. T., & Wolke, D. (2013). Prenatal family adversity and maternal mental health and vulnerability to peer victimisation at school. Journal of Child Psychology and Psychiatry, 54(6), 644-652. | Outcome not of interest |
| Lereya, S. T., Winsper, C., Heron, J., Lewis, G., Gunnell, D., Fisher, H. L., & Wolke, D. (2013). Being bullied during childhood and the prospective pathways to self-harm in late adolescence. Journal of the American Academy of Child & Adolescent Psychiatry, 52(6), 608-618. | Outcome not of interest |
| Lester, L., Pearce, N., Waters, S., Barnes, A., Beatty, S., & Cross, D. (2017). Family involvement in a whole-school bullying intervention: Mothers’ and fathers’ communication and influence with children. Journal of Child and Family Studies, 26(10), 2716-2727. | Outcome not of interest |
| Levi‐Belz, Y., Gavish‐Marom, T., Barzilay, S., Apter, A., Carli, V., Hoven, C., ... & Wasserman, D. (2019). Psychosocial factors correlated with undisclosed suicide attempts to significant others: findings from the adolescence SEYLE study. Suicide and Life‐Threatening Behavior, 49(3), 759-773. | Outcome not of interest |
| Li, C., Tan, X., Zhao, Q., & Ren, P. (2022). Parental warmth may not always be beneficial: high parental warmth impairs victimized adolescents’ academic achievement via elevated school burnout. Journal of Social and Personal Relationships, 39(6), 1863-1884. | Full text not available |
| Li, D. J., Chang, Y. P., Chen, Y. L., & Yen, C. F. (2020). The impact of emotional symptoms and family support on the association between homophobic bullying and sedative/hypnotic use among gay and bisexual men in Taiwan: A moderated mediation model. International journal of environmental research and public health, 17(11), 3870. | Population not of interest |
| Li, J., & Hesketh, T. (2021). Experiences and perspectives of traditional bullying and cyberbullying among adolescents in Mainland China-implications for policy. Frontiers in psychology, 12, 672223. | No validated measure for PF |
| Li, J., Sha, S., Luo, W., Zhou, W., Dong, Y., & Zhang, S. (2023). Prevalence and associated factors of bullying victimization among Chinese high school students in Shandong, China. Journal of affective disorders, 323, 667-674. | No response |
| Li, J., Sidibe, A. M., Shen, X., & Hesketh, T. (2019). Incidence, risk factors and psychosomatic symptoms for traditional bullying and cyberbullying in Chinese adolescents. Children and Youth Services Review, 107, 104511. | No validated measure for PF |
| Li, Y., Kang, Y., Zhu, L., Yuan, M., Li, Y., Xu, B., ... & Su, P. (2023). Longitudinal correlates of bullying victimization among Chinese early adolescents: a cross-lagged panel network analysis. Journal of affective disorders, 339, 203-210. | No validated measure for PF |
| Lian, Q., Yu, C., Tu, X., Deng, M., Wang, T., Su, Q., & Zuo, X. (2021). Grade repetition and bullying victimization in adolescents: A global cross-sectional study of the Program for International Student Assessment (PISA) data from 2018. PLoS medicine, 18(11), e1003846. | Outcome not of interest |
| Lin, P. C., Yen, C. N., Lin, H. C., Wang, P. W., Liu, T. L., Hu, H. F., & Yen, C. F. (2018). Relationships between involvement in school bullying and quality of life in Taiwanese adolescents. Applied Research in Quality of Life, 13(3), 545-559. | No response |
| Lin, Y. C., Latner, J. D., Fung, X. C., & Lin, C. Y. (2018). Poor health and experiences of being bullied in adolescents: Self‐perceived overweight and frustration with appearance matter. Obesity, 26(2), 397-404. | No validated measure for PF |
| Liu, J., Guo, S., Weissman, R., & Liu, H. (2020). Investigating factors associated with bullying utilizing latent class analysis among adolescents. School Psychology International, 0143034320967061. | No validated measure for PF |
| Liu, J., Guo, S., Weissman, R., & Liu, H. (2021). Investigating factors associated with bullying utilizing latent class analysis among adolescents. School psychology international, 42(1), 11-32. | No response |
| Liu, T. L., Hsiao, R. C., Chou, W. J., & Yen, C. F. (2021). Self-reported depressive symptoms and suicidality in adolescents with attention-deficit/hyperactivity disorder: roles of bullying involvement, frustration intolerance, and hostility. International journal of environmental research and public health, 18(15), 7829. | Outcome not of interest |
| Liu, T., & Li, T. Social bonds, Internet usage time and bullying involvement: A study of rural secondary school students in China. Child & Family Social Work. | Full text not available |
| Liu, W., Qiu, G., Zhang, S. X., & Fan, Q. (2022). Corporal punishment, self-control, parenting style, and school bullying among Chinese adolescents: a conditional process analysis. Journal of school violence, 21(1), 4-18. | Outcome not of interest |
| Liu, X., Chen, G., Hu, P., Guo, G., & Xiao, S. (2017). Does perceived social support mediate or moderate the relationship between victimisation and suicidal ideation among Chinese adolescents?. Journal of psychologists and counsellors in schools, 27(1), 123. | No response |
| Liu, Y., & Hu, F. (2021). Being bullied at school as a child, worse health as an adult? Evidence from China. International Journal of Educational Development, 82, 102374. | Population not of interest |
| Liu, Y., Mendonça, M., Bartmann, P., & Wolke, D. (2022). Very preterm birth and trajectories of domain-specific self-concept from childhood into adulthood. Development and Psychopathology, 34(5), 1926-1937. | No validated measure for PF |
| Livazović, G., & Ham, E. (2019). Cyberbullying and emotional distress in adolescents: the importance of family, peers and school. Heliyon, 5(6), e01992. | Population not of interest |
| Lo, C. C., Ash-Houchen, W., Cheng, T. C., & Chu, D. C. (2022). Victimization and Bullying in Taiwan. Violence and Victims, 37(6), 799-819. | Full text not available |
| Lo, C. K., Ho, F. K., Emery, C., Chan, K. L., Wong, R. S., Tung, K. T., & Ip, P. (2021). Association of harsh parenting and maltreatment with internet addiction, and the mediating role of bullying and social support. Child Abuse & Neglect, 113, 104928. | Outcome not of interest |
| Lo, C. K., Ho, F. K., Emery, C., Chan, K. L., Wong, R. S., Tung, K. T., & Ip, P. (2021). Association of harsh parenting and maltreatment with internet addiction, and the mediating role of bullying and social support. Child Abuse & Neglect, 113, 104928. | Outcome not of interest |
| Lohbeck, A., & Petermann, F. (2018). Cybervictimization, self-esteem, and social relationships among German secondary school students. Journal of school violence, 17(4), 472-486. | Outcome not of interest |
| Lopes, B. C. (2013). Differences between victims of bullying and nonvictims on levels of paranoid ideation and persecutory symptoms, the presence of aggressive traits, the display of social anxiety and the recall of childhood abuse experiences in a Portuguese mixed clinical sample. Clinical psychology & psychotherapy, 20(3), 254-266. | Population not of interest |
| López-Catalán, B., Mäkela, T., Sánchez Sánchez, F. J., & López Catalán, L. (2022). Implementing kiva antibullying program in pioner schools in Spain. | Outcome not of interest |
| Lorenzo-Blanco, E. I., Unger, J. B., Oshri, A., Baezconde-Garbanati, L., & Soto, D. (2016). Profiles of bullying victimization, discrimination, social support, and school safety: Links with Latino/a youth acculturation, gender, depressive symptoms, and cigarette use. American journal of orthopsychiatry, 86(1), 37. | Outcome not of interest |
| Low, S., & Liu, L. (2022). Contribution of School and Family Factors to Racial Disparities in Bullying Involvement. Journal of Early Adolescence. | Outcome not of interest |
| Lucas, S., Jernbro, C., Tindberg, Y., & Janson, S. (2016). Bully, bullied and abused. Associations between violence at home and bullying in childhood. Scandinavian journal of public health, 44(1), 27-35. | Outcome not of interest |
| Luk, J. W., Gilman, S. E., Haynie, D. L., & Simons-Morton, B. G. (2018). Sexual orientation and depressive symptoms in adolescents. Pediatrics, 141(5). | Outcome not of interest |
| Lussier, P., Chouinard-Thivierge, S., McCuish, E., Nadeau, D., & Lacerte, D. (2019). Early life adversities and polyvictimization in young persons with sexual behavior problems: A longitudinal study of child protective service referrals. Child abuse & neglect, 88, 37-50. | Outcome not of interest |
| Ma, L., Phelps, E., Lerner, J. V., & Lerner, R. M. (2009). The development of academic competence among adolescents who bully and who are bullied. Journal of applied developmental psychology, 30(5), 628-644. | Outcome not of interest |
| Ma, X. (2001). Bullying and being bullied: To what extent are bullies also victims?. American Educational Research Journal, 38(2), 351-370. | No validated measure for PF |
| Maftei, A., & Măirean, C. (2023). Not so funny after all! Humor, parents, peers, and their link with cyberbullying experiences. Computers in Human Behavior, 138, 107448. | Outcome not of interest |
| Mahumud, R. A., Dawson, A. J., Chen, W., Biswas, T., Keramat, S. A., Morton, R. L., & Renzaho, A. M. (2022). The risk and protective factors for suicidal burden among 251 763 school-based adolescents in 77 low-and middle-income to high-income countries: assessing global, regional and national variations. Psychological medicine, 52(2), 379-397. | Outcome not of interest |
| Malaeb, D., Awad, E., Haddad, C., Salameh, P., Sacre, H., Akel, M., ... & Hallit, S. (2020). Bullying victimization among Lebanese adolescents: The role of child abuse, Internet addiction, social phobia and depression and validation of the Illinois Bully Scale. BMC pediatrics, 20(1), 1-11. | Outcome not of interest |
| Mallmann, C. L., de Macedo Lisboa, C. S., & Zanatta Calza, T. (2018). Cyberbullying and coping strategies in adolescents from Southern Brazil. Acta Colombiana de Psicología, 21(1), 13-43. | Outcome not of interest |
| Malta, D. C., Antunes, J. T., Prado, R. R. D., Assunção, A. Á., & Freitas, M. I. D. (2019). Factors associated with family violence against adolescents based on the results of the National School Health Survey (PeNSE). Ciencia & saude coletiva, 24, 1287-1298. | No validated measure for PF |
| Malta, D. C., Mello, F. C. M. D., Prado, R. R. D., Sá, A. C. M. G. N. D., Marinho, F., Pinto, I. V., ... & Silva, M. A. I. (2019). Prevalence of bullying and associated factors among Brazilian schoolchildren in 2015. Ciencia & saude coletiva, 24, 1359-1368. | No validated measure for PF |
| Malta, D. C., Prado, R. R. D., Dias, A. J. R., Mello, F. C. M., Silva, M. A. I., Costa, M. R. D., & Caiaffa, W. T. (2014). Bullying and associated factors among Brazilian adolescents: analysis of the National Adolescent School-based Health Survey (PeNSE 2012). Revista Brasileira de Epidemiologia, 17, 131-145. | No validated measure for PF |
| Man, X., Liu, J., & Xue, Z. (2022). Effects of bullying forms on adolescent mental health and protective factors: a global cross-regional research based on 65 countries. International journal of environmental research and public health, 19(4), 2374. | No response |
| Mang, L., Huang, N., Liu, X., Zhen, C., & Guo, J. (2023). The interaction effects of social support and four types of bullying on sleep quality in Chinese adolescents. Journal of affective disorders, 341, 119-127. | Outcome not of interest |
| Mann, M. J., Kristjansson, A. L., Sigfusdottir, I. D., & Smith, M. L. (2015). The role of community, family, peer, and school factors in group bullying: implications for school‐based intervention. Journal of school health, 85(7), 477-486. | No validated measure for PF |
| Marengo, N., Borraccino, A., Charrier, L., Berchialla, P., Dalmasso, P., Caputo, M., & Lemma, P. (2021). Cyberbullying and problematic social media use: an insight into the positive role of social support in adolescents—data from the Health Behaviour in School-aged Children study in Italy. Public Health, 199, 46-50. | Outcome not of interest |
| Marini, Z. A., Dane, A. V., Bosacki, S. L., & CURA, Y. (2006). Direct and indirect bully‐victims: Differential psychosocial risk factors associated with adolescents involved in bullying and victimization. Aggressive Behavior: Official Journal of the International Society for Research on Aggression, 32(6), 551-569. | Outcome not of interest |
| Markota, M., McKean, A. J., Romanowicz, M., Schak, K. M., Croarkin, P. E., & Voort, J. L. V. (2018). Rehospitalization to a child and adolescent psychiatry unit: Role of trauma and bullying. General hospital psychiatry, 55, 10-14. | Outcome not of interest |
| Martin-Criado, J. M., Casas, J. A., Ortega-Ruiz, R., & Del Rey, R. (2021). Supervisión parental y víctimas de ciberbullying: influencia del uso de redes sociales y la extimidad online. Revista de Psicodidáctica. | Another language |
| Martin-Criado, J. M., Casas, J. A., Ortega-Ruiz, R., & Del Rey, R. (2021). Parental supervision and victims of cyberbullying: Influence of the use of social networks and online extimacy. Revista de Psicodidáctica (English ed.), 26(2), 160-167. | Another language |
| Martínez, I., Murgui, S., García, O. F., & García, F. (2019). Parenting in the digital era: Protective and risk parenting styles for traditional bullying and cyberbullying victimization. Computers in Human Behavior, 90, 84-92. | No response |
| Martins, M. J. D., Simão, A. M. V., Freire, I., Caetano, A. P., & Matos, A. (2017). Cyber-victimization and cyber-aggression among Portuguese adolescents: The relation to family support and family rules. In Violence and society: Breakthroughs in research and practice (pp. 134-149). IGI Global. | No validated measure for PF |
| Matsunaga, M. (2009). Parents Don’t (Always) Know Their Children Have Been Bullied: Child‐Parent Discrepancy on Bullying and Family‐Level Profile of Communication Standards. Human Communication Research, 35(2), 221-247. | Population not of interest |
| Mauer, V. A., Waterman, E. A., Edwards, K. M., & Banyard, V. L. (2022). Adolescents' Relationships With Important Adults: Exploring This Novel Protective Factor Against Interpersonal Violence Victimization and Perpetration. Journal of interpersonal violence, 37(19-20), NP19176-NP19187. | Full text not available |
| Maurya, C., Muhammad, T., Das, A., Fathah, A., & Dhillon, P. (2023). The role of self-efficacy and parental communication in the association between cyber victimization and depression among adolescents and young adults: a structural equation model. BMC psychiatry, 23(1), 337. | Population not of interest |
| Mazaba-Liwewe, M. L., Pasupulati, S., Rudatsikira, E., Babaniyi, O., Ndumba, I., Masaninga, F., ... & Siziya, S. (2015). Bullying victimization in Benin: Prevalence and its correlates among in-school adolescents. International Journal of Child and Adolescent Health, 8(1), 75. | No response |
| McComb, S. E., & Dane, A. V. (2019). Evolutionary psychological perspective on peer victimization: Relations with attachment security and dating and sexual history. Evolutionary Psychological Science, 5(2), 243-255. | Outcome not of interest |
| McField, A. A., Lawrence, T. I., & Okoli, I. C. (2023). Examining the relationships between cyberbullying, relational victimization, and family support on depressive symptoms and substance use among adolescents. Clinical Child Psychology and Psychiatry, 28(1), 224-236. | No validated measure for PF |
| McVie, S. (2014). The impact of bullying perpetration and victimization on later violence and psychological distress: a study of resilience among a Scottish youth cohort. Journal of School Violence, 13(1), 39-58. | Outcome not of interest |
| Meinck, F., Cluver, L. D., Boyes, M. E., & Ndhlovu, L. D. (2015). Risk and protective factors for physical and emotional abuse victimisation amongst vulnerable children in South Africa. Child Abuse Review, 24(3), 182-197. | Population not of interest |
| Melancon, C., & Gagne, M. H. (2011). Father’s and mother’s psychological violence and adolescent behavioral adjustment. Journal of Interpersonal Violence, 26(5), 991-1011. | Outcome not of interest |
| Melander, L. A., Hartshorn, K. J. S., & Whitbeck, L. B. (2013). Correlates of bullying behaviors among a sample of North American Indigenous adolescents. Journal of adolescence, 36(4), 675-684. | Couldn't provide correlation |
| Mello, F. C. M., Malta, D. C., Prado, R. R. D., Farias, M. S., Alencastro, L. C. D. S., & Silva, M. A. I. (2016). Bullying and associated factors in adolescents in the Southeast region according to the National School-based Health Survey. Revista Brasileira de Epidemiologia, 19, 866-877. | No validated measure for PF |
| Mello, F. C. M., Silva, J. L. D., Oliveira, W. A. D., Prado, R. R. D., Malta, D. C., & Silva, M. A. I. (2017). The practice of bullying among Brazilian schoolchildren and associated factors, National School Health Survey 2015. Ciencia & saude coletiva, 22, 2939-2948. | Outcome not of interest |
| Mena, A. J., & Arteche, A. (2019). INFLUENCIA DE LA PERSONALIDAD, LA REGULACIÓN EMOCIONAL, LOS ESTILOS PARENTALES Y ALGUNAS CARACTERÍSTICAS SOCIODEMOGRÁFICAS SOBRE EL ACOSO ESCOLAR EN UN GRUPO DE ADOLESCENTES BRASILEÑOS. Revista Interamericana de Psicologia/Interamerican Journal of Psychology (IJP), 53(3), 380-398. | Another language |
| Mendoza González, B. (2017). Upbringing and bullying: a description of students at the basic education level. Innovación educativa (México, DF), 17(74), 125-141. | Another language |
| Meng, Y., Yang, Y., Lin, P., Xiao, Y., Sun, Y., Qian, Y., ... & Zhang, X. (2023). School bullying victimization and associated factors among school-aged adolescents in China. Journal of interpersonal violence, 38(1-2), 1787-1814. | No response |
| Merrick, M. T., Henly, M., Turner, H. A., David-Ferdon, C., Hamby, S., Kacha-Ochana, A., ... & Finkelhor, D. (2018). Beyond residential mobility: A broader conceptualization of instability and its impact on victimization risk among children. Child abuse & neglect, 79, 485-494. | Outcome not of interest |
| Mesch, G. S. (2009). Parental mediation, online activities, and cyberbullying. CyberPsychology & Behavior, 12(4), 387-393. | No validated measure for PF |
| Mesch, G. S. (2018). Parent–child connections on social networking sites and cyberbullying. Youth & Society, 50(8), 1145-1162. | No validated measure for PF |
| Michaelson, V., Donnelly, P., Morrow, W., King, N., Craig, W., & Pickett, W. (2021). Violence, adolescence, and Canadian religious communities: a quantitative study. Journal of interpersonal violence, 36(7-8), 3613-3637. | Outcome not of interest |
| Mikhaylova, O., & Bochaver, A. (2022, May). Family Environment Hostility as the Missing Element Between School Bullying Victimization and Anorexia. In Frontiers in Education (Vol. 7, p. 880994). Frontiers Media SA. | Qualitative study |
| Mintz, S., Valido, A., Rivas-Koehl, M., Kuehl, T., Espelage, D. L., Woolweaver, A., & Ingram, K. M. (2021). Supporting sexual minority youth: Protective factors of adverse health outcomes and implications for public health. Journal of Adolescent Health, 69(6), 983-990. | No validated measure for PF |
| Miranda, R., Oriol, X., Amutio, A., & Ortúzar, H. (2019). Adolescent bullying victimization and life satisfaction: Can family and school adult support figures mitigate this effect?. Revista de Psicodidáctica (English ed.), 24(1), 39-45. | Outcome not of interest |
| Mischel, J., & Kitsantas, A. (2020). Middle school students' perceptions of school climate, bullying prevalence, and social support and coping. Social Psychology of Education, 23(1), 51-72. | Outcome not of interest |
| Mishna, F., Khoury-Kassabri, M., Gadalla, T., & Daciuk, J. (2012). Risk factors for involvement in cyber bullying: Victims, bullies and bully–victims. Children and Youth Services Review, 34(1), 63-70. | No validated measure for PF |
| Mishna, F., Khoury-Kassabri, M., Schwan, K., Wiener, J., Craig, W., Beran, T., ... & Daciuk, J. (2016). The contribution of social support to children and adolescents' self-perception: The mediating role of bullying victimization. Children and Youth Services Review, 63, 120-127. | Outcome not of interest |
| Miura, H., & Fuchigami, Y. (2020). Influence of maltreatment, bullying, and neurocognitive impairment on recidivism in adolescents with conduct disorder: A 3-Year prospective study. Applied Neuropsychology: Child, 1-10. | Outcome not of interest |
| Mlisa, L. N., Ward, C. L., Flisher, A. J., & Lombard, C. J. (2008). Bullying at rural high schools in the Eastern Cape Province, South Africa: Prevalence, and risk and protective factors at school and in the family. Journal of Psychology in Africa, 18(2), 261-267. | Population not of interest |
| Mobin, A., Feng, C. X., & Neudorf, C. (2017). Cybervictimization among preadolescents in a community-based sample in Canada: Prevalence and predictors. Canadian journal of public health, 108(5), e475-e481. | No validated measure for PF |
| Moffat, A. K., Redmond, G., & Raghavendra, P. (2019). The impact of social network characteristics and gender on covert bullying in Australian students with disability in the middle years. Journal of school violence, 18(4), 613-629. | No response |
| Mohapatra, S., Irving, H., Paglia‐Boak, A., Wekerle, C., Adlaf, E., & Rehm, J. (2010). History of family involvement with child protective services as a risk factor for bullying in Ontario schools. Child and Adolescent Mental Health, 15(3), 157-163. | Population not of interest |
| Mohebbi, M., Mirnasab, M., & Wiener, J. (2016). Parental and school bonding in Iranian adolescent perpetrators and victims of bullying. School psychology international, 37(6), 583-605. | Population not of interest |
| Monks, C. P., Mahdavi, J., & Rix, K. (2016). The emergence of cyberbullying in childhood: Parent and teacher perspectives. Psicología Educativa, 22(1), 39-48. | Qualitative study |
| Monks, C. P., Smith, P. K., & Swettenham, J. (2005). Psychological correlates of peer victimisation in preschool: Social cognitive skills, executive function and attachment profiles. Aggressive Behavior: Official Journal of the International Society for Research on Aggression, 31(6), 571-588. | Outcome not of interest |
| Monopoli, W. J., Evans, S. W., & Himawan, L. K. (2022). Risk and protective factors for patterns of bullying involvement in middle school students. Journal of school violence, 21(2), 175-189. | No response |
| Moon, B., Blurton, D., & McCluskey, J. D. (2008). General strain theory and delinquency: Focusing on the influences of key strain characteristics on delinquency. Crime & delinquency, 54(4), 582-613. | No validated measure for PF |
| Moon, S. S., Kim, H., Seay, K., Small, E., & Kim, Y. K. (2016). Ecological factors of being bullied among adolescents: A classification and regression tree approach. Child indicators research, 9(3), 743-756. | Outcome not of interest |
| Moral Jiménez, M. D. L. V., & Ovejero, A. (2021). Adolescents’ attitudes to bullying and its relationship to perceived family social climate [actitudes de los adolescentes hacia el bullying y su relación con el clima social familiar percibido.]. Psicothema. | Outcome not of interest |
| Moreno–Ruiz, D., Martínez–Ferrer, B., & García–Bacete, F. (2019). Parenting styles, cyberaggression, and cybervictimization among adolescents. Computers in Human Behavior, 93, 252-259. | No response |
| Morgan, P. L., Farkas, G., Woods, A. D., Wang, Y., Hillemeier, M. M., & Oh, Y. (2023). Factors Predictive of Being Bullies or Victims of Bullies in US Elementary Schools. School Mental Health, 1-17. | Outcome not of interest |
| Morgan, P. L., Woods, A. D., Wang, Y., Farkas, G., Oh, Y., Hillemeier, M. M., & Mitchell, C. (2022). Which children are frequently victimized in US elementary schools? Population-based estimates. School mental health, 14(4), 1011-1023. | Outcome not of interest |
| Morin, H. K., Bradshaw, C. P., & Berg, J. K. (2015). Examining the link between peer victimization and adjustment problems in adolescents: The role of connectedness and parent engagement. Psychology of Violence, 5(4), 422. | Outcome not of interest |
| Mortier, P., Alonso, J., Auerbach, R. P., Bantjes, J., Benjet, C., Bruffaerts, R., ... & Kessler, R. C. (2022). Childhood adversities and suicidal thoughts and behaviors among first-year college students: results from the WMH-ICS initiative. Social psychiatry and psychiatric epidemiology, 57(8), 1591-1601. | Population not of interest |
| Muller, R. D., Skues, J. L., & Wise, L. Z. (2017). Cyberbullying in Australian primary schools: How victims differ in attachment, locus of control, self-esteem, and coping styles compared to non-victims. Journal of psychologists and counsellors in schools, 27(1), 85. | No response |
| Mulvey, K. L., Hoffman, A. J., Gönültaş, S., Hope, E. C., & Cooper, S. M. (2018). Understanding experiences with bullying and bias-based bullying: What matters and for whom?. Psychology of violence, 8(6), 702. | Outcome not of interest |
| Murphy, T. P., Laible, D., & Augustine, M. (2017). The influences of parent and peer attachment on bullying. Journal of child and family studies, 26(5), 1388-1397. | Outcome not of interest |
| Murray-Harvey, R., & Slee, P. T. (2007). Supportive and stressful relationships with teachers, peers and family and their influence on students' social/emotional and academic experience of school. Australian Journal of Guidance and Counselling, 17(2), 126. | No response |
| Mustanoja, S., Luukkonen, A. H., Hakko, H., Räsänen, P., Säävälä, H., & Riala, K. (2011). Is exposure to domestic violence and violent crime associated with bullying behaviour among underage adolescent psychiatric inpatients?. Child Psychiatry & Human Development, 42(4), 495-506. | Outcome not of interest |
| Mutiso, V. N., Musyimi, C. W., Krolinski, P., Neher, C. M., Musau, A. M., Tele, A., & Ndetei, D. M. (2019). Relationship between bullying, substance use, psychiatric disorders, and social problems in a sample of Kenyan secondary schools. Prevention science, 20(4), 544-554. | Population not of interest |
| Muula, A. S., Herring, P., Siziya, S., & Rudatsikira, E. (2009). Bullying victimization and physical fighting among Venezuelan adolescents in Barinas: results from the Global School-Based Health Survey 2003. Italian Journal of Pediatrics, 35(1), 1-5. | No response |
| Myklestad, I., & Straiton, M. (2021). The relationship between self-harm and bullying behaviour: results from a population based study of adolescents. BMC public health, 21(1), 1-15. | Population not of interest |
| Narita, Z., DeVylder, J., Yamasaki, S., Ando, S., Endo, K., Miyashita, M., ... & Nishida, A. (2023). Uncovering associations between gender nonconformity, psychosocial factors, and mental health in adolescents: a birth cohort study. Psychological medicine, 1-10. | Outcome not of interest |
| Natesan, P., Mitchell, M. E., & Glover, R. J. (2018, June). Early Predictors of Child's Bully and Victim Statuses: A Longitudinal Investigation Using Parent, Teacher, and Student Reports From National Data. In Frontiers in Education (Vol. 3, p. 48). Frontiers. | No response |
| Nation, M., Vieno, A., Perkins, D. D., & Santinello, M. (2008). Bullying in school and adolescent sense of empowerment: An analysis of relationships with parents, friends, and teachers. Journal of community & applied social psychology, 18(3), 211-232. | No validated measure for PF |
| Ngo, A. T., Nguyen, L. H., Dang, A. K., Hoang, M. T., Nguyen, T. H. T., Vu, G. T., ... & Ho, C. S. (2021). Bullying experience in urban adolescents: Prevalence and correlations with health-related quality of life and psychological issues. PloS one, 16(6), e0252459. | Outcome not of interest |
| Ngo, A. T., Tran, A. Q., Tran, B. X., Nguyen, L. H., Hoang, M. T., Nguyen, T. H. T., ... & Ho, C. S. (2021). Cyberbullying among school adolescents in an urban setting of a developing country: experience, coping strategies, and mediating effects of different support on psychological well-being. Frontiers in psychology, 12, 661919. | No validated measure for PF |
| Nguyen, H. T. L., Nakamura, K., & Seino, K. (2020). Relationships among cyberbullying, parental attitudes, self-harm and suicidal behavior among adolescents: results from a school-based survey in Vietnam. BMC public health, 20(1), 1-9. | No response |
| Nicholson, J. V., Chen, Y., & Huang, C. C. (2018). Children's exposure to intimate partner violence and peer bullying victimization. Children and Youth Services Review, 91, 439-446. | Outcome not of interest |
| Nicholson, J. V., Chen, Y., & Huang, C. C. (2018). Children's exposure to intimate partner violence and peer bullying victimization. Children and Youth Services Review, 91, 439-446. | Outcome not of interest |
| Nickerson, A. B., Shisler, S., Eiden, R. D., Ostrov, J. M., Schuetze, P., Godleski, S. A., & Delmerico, A. M. (2020). A longitudinal study of gun violence attitudes: role of childhood aggression and exposure to violence, and early adolescent bullying perpetration and victimization. Journal of school violence, 19(1), 62-76. | Outcome not of interest |
| Nilsson, D. K., Gustafsson, P. E., & Svedin, C. G. (2012). Lifetime polytraumatization in adolescence and being a victim of bullying. The Journal of nervous and mental disease, 200(11), 954-961. | Outcome not of interest |
| Noipom, R., Lim, A., Sittichai, R., Sukkhum, S., & Dureh, N. (2023). Factors Associated with Negative Emotional Responses and Maladaptive Coping Strategies to Cyberbullying Among Young People in Southernmost Thailand. Progress in Psychiatry and Behavioral Sciences, (In Press). | Population not of interest |
| Nolan, E., Bunting, L., McCartan, C., Davidson, G., Grant, A., Schubotz, D., ... & Shevlin, M. (2023). Prevalence of probable eating disorders and associated risk factors: An analysis of the Northern Ireland Youth Wellbeing Survey using the SCOFF. British Journal of Clinical Psychology, 62(1), 180-195. | Outcome not of interest |
| Noret, N., Hunter, S. C., & Rasmussen, S. (2020). The role of perceived social support in the relationship between being bullied and mental health difficulties in adolescents. School Mental Health, 12(1), 156-168. | No validated measure for PF |
| Norton, J. R., & Duke, A. M. (2021). The influence of parenting on bullying prevention: Parenting as a moderator of adolescents’ bullying behaviors. Journal of Child and Family Studies, 30, 2913-2924. | Outcome not of interest |
| Nurius, P., LaValley, K., & Kim, M. H. (2020). Victimization, poverty, and resilience resources: Stress process considerations for adolescent mental health. School mental health, 12(1), 124-135. | No validated measure for PF |
| Obeid, S., Sacre, H., Hallit, S., & Salameh, P. (2020). School Bullying—The Silent Epidemic: A Cross-Sectional Study of Factors Associated With Peer Victimization Among Lebanese Adolescents. Journal of interpersonal violence, 0886260520922376. | Outcome not of interest |
| O'Hara, M. A. (2020). Peer victimization of maltreated youth: distinct risk for physically abused versus neglected children. Journal of school health, 90(6), 457-464. | Outcome not of interest |
| Ohene, S. A., Ireland, M., McNeely, C., & Borowsky, I. W. (2006). Parental expectations, physical punishment, and violence among adolescents who score positive on a psychosocial screening test in primary care. Pediatrics, 117(2), 441-447. | No validated measure for PF |
| Ohene, S. A., Johnson, K., Atunah-Jay, S., Owusu, A., & Borowsky, I. W. (2015). Sexual and physical violence victimization among senior high school students in Ghana: Risk and protective factors. Social Science & Medicine, 146, 266-275. | Population not of interest |
| Okada, L. M., Miranda, R. R., das Graças Pena, G., Levy, R. B., & Azeredo, C. M. (2019). Association between exposure to interpersonal violence and social isolation, and the adoption of unhealthy weight control practices. Appetite, 142, 104384. | No validated measure for PF |
| OLIVEIRA, W. A. D., SILVA, J. L., Fernandez, J. E. R., SANTOS, M. A. D., Caravita, S. C. S., & Silva, M. A. I. (2020). Family interactions and the involvement of adolescents in bullying situations from a bioecological perspective. Estudos de Psicologia (Campinas), 37. | No response |
| Olweus, D. (1997). Bully/victim problems in school: Facts and intervention. European journal of psychology of education, 12(4), 495-510. | Qualitative study |
| O'Moore, A. M., & Minton, S. J. (2005). Evaluation of the effectiveness of an anti‐bullying programme in primary schools. Aggressive Behavior: Official Journal of the International Society for Research on Aggression, 31(6), 609-622. | Outcome not of interest |
| Ong, M. S., Lakoma, M., Gees Bhosrekar, S., Hickok, J., McLean, L., Murphy, M., ... & Ross‐Degnan, D. (2021). Risk factors for suicide attempt in children, adolescents, and young adults hospitalized for mental health disorders. Child and adolescent mental health, 26(2), 134-142. | Population not of interest |
| Ortega-Barón, J., Buelga-Vasquez, S., & Cava-Caballero, M. J. (2016). The Influence of School Climate and Family Climate among Adolescents Victims of Cyberbullying= Influencia del clima escolar y familiar en adolescentes, víctimas de ciberacoso. Comunicar, 24(46), 57-65. | Methodological problems |
| Ossorno García, S. D., Martín Babarro, J., & Toldos, M. (2019). Ecological-transaction model approach of adolescents’ parental maltreatment and peer-bullying: the moderating role of bullying at the classroom. | Outcome not of interest |
| Ostrov, J. M., Perry, K. J., Eiden, R. D., Nickerson, A. B., Schuetze, P., Godleski, S. A., & Shisler, S. (2022). Development of bullying and victimization: An examination of risk and protective factors in a high-risk sample. Journal of interpersonal violence, 37(9-10), 5958-5984. | Outcome not of interest |
| Otake, Y., Liu, X., & Luo, X. (2019). Involvement in bullying among left-behind children in provincial chinese cities: the role of perceived emotional support. Journal of Aggression, Maltreatment & Trauma, 28(8), 943-957. | No response |
| Ouellet-Morin, I., Danese, A., Bowes, L., Shakoor, S., Ambler, A., Pariante, C. M., ... & Arseneault, L. (2011). A discordant monozygotic twin design shows blunted cortisol reactivity among bullied children. Journal of the American Academy of Child & Adolescent Psychiatry, 50(6), 574-582. | Outcome not of interest |
| Ouyang, C., Li, D., Li, X., Xiao, J., Sun, W., & Wang, Y. (2020). Cyber victimization and tobacco and alcohol use among adolescents: A moderated mediation model. Children and youth services review, 114, 105041. | Outcome not of interest |
| Özdemir, E. Z., & Bektaş, M. (2021). The effects of self-efficacy and locus of control on cyberbully/victim status in adolescents. Journal of Pediatric Nursing, 61, e15-e21. | Couldn't provide correlation |
| Özdemir, E. Z., & Bektaş, M. (2022). Effects of parental attitude and proactive and reactive aggression on cyberbullying and victimization among secondary school students. Perspectives in Psychiatric Care, 58(2), 830-839. | Couldn't provide correlation |
| Özdemir, S. (2018). Analysis of Deviant Friends' Mediator Effect on Relationships Between Adolescent Risk Behaviours and Peer Bullying, Abuse Experiences and Psychological Resilience. Education & Science/Egitim ve Bilim, 43(195). | Outcome not of interest |
| Pabian, S. (2019). An investigation of the effectiveness and determinants of seeking support among adolescent victims of cyberbullying. The Social Science Journal, 56(4), 480-491. | No validated measure for PF |
| Pace, U., D’Urso, G., & Fontanesi, L. (2020). The vicissitudes of homophobic victimization in adolescence: an explorative study. Frontiers in psychology, 11, 43. | Population not of interest |
| Paek, S. Y., Lee, J., & Choi, Y. J. (2022). The impact of parental monitoring on cyberbullying victimization in the COVID‐19 era. Social Science Quarterly, 103(2), 294-305. | No validated measure for PF |
| Palladino, B. E., Pini, S., Nocentini, A., & Menesini, E. (2014). Il ciclo della violenza: maltrattamento familiare, bullismo e dating aggression psicologico. Maltrattamento e abuso all’infanzia. | Another language |
| Pandey, A. R., Neupane, T., Chalise, B., Shrestha, N., Chaudhary, S., Dhungana, R. R., & Bista, B. (2021). Factors associated with physical and sexual violence among school-going adolescents in Nepal: Findings from Global School-based Student Health Survey. PLoS ONE, 16(3). | Outcome not of interest |
| Paniagua, C., García-Moya, I., Sánchez-Queija, I., & Moreno, C. (2022). Bullying, cyberbullying, and adoption: What is the role of student–teacher connectedness?. School psychology. | Full text not available |
| Panzer, B. M., & Dhuper, S. (2014). Designing a group therapy program for coping with childhood weight bias. Social work, 59(2), 141-147. | Outcome not of interest |
| Paredes, P. P., & Calvete, E. (2014). Cognitive vulnerabilities as mediators between emotional abuse and depressive symptoms. Journal of abnormal child psychology, 42(5), 743-753. | Outcome not of interest |
| Park, I. Y., Walls, N. E., Atteberry-Ash, B., & Kattari, L. (2023). Trusted Adults as a Protective Factor for Bullying and Suicide Attempts Among Youth Across Differences in Race, Sexual Orientation, and Gender Identity. Journal of the Society for Social Work and Research, 14(2), 313-336. | Full text not available |
| Park, J., Grogan-Kaylor, A., & Han, Y. (2021). Trajectories of Childhood Maltreatment and Bullying of Adolescents in South Korea. Journal of Child and Family Studies, 30(4), 1059-1070. | Outcome not of interest |
| Park, S., Na, E. Y., & Kim, E. M. (2014). The relationship between online activities, netiquette and cyberbullying. Children and youth services review, 42, 74-81. | No validated measure for PF |
| Park, T. Y., Cui, C., Park, Y., Kim, K., Moon, H., Kim, H., ... & Lee, H. (2023). Multiple Case Study on Family Therapy for Middle School Bullying Victims in South Korea. The American Journal of Family Therapy, 51(4), 356-376. | Qualitative study |
| Pascual‐Sanchez, A., Mateu, A., Martinez‐Herves, M., Hickey, N., Kramer, T., & Nicholls, D. (2022). How are parenting practices associated with bullying in adolescents? A cross‐sectional study. Child and adolescent mental health, 27(3), 223-231. | Population not of interest |
| Paterson, J., Lusitini, L., & Taylor, S. (2014). Pacific Islands Families Study: depressive symptoms in 9-year-old Pacific children living in New Zealand. The New Zealand Medical Journal (Online), 127(1390). | Outcome not of interest |
| Peker, A. (2015). Analyzing the risk factors predicting the cyberbullying status of secondary school students. Egitim Ve Bilim, 40(181). | No validated measure for PF |
| Peltzer, K., & Pengpid, S. (2020). Prevalence of bullying victimisation and associated factors among in-school adolescents in Mozambique. Journal of psychology in Africa, 30(1), 64-68. | No response |
| Peng, K., Zhu, X., Gillespie, A., Wang, Y., Gao, Y., Xin, Y., ... & Chen, R. (2019). Self-reported rates of abuse, neglect, and bullying experienced by transgender and gender-nonbinary adolescents in China. JAMA network open, 2(9), e1911058-e1911058. | No validated measure for PF |
| Pengpi, S., & Peltzer, K. (2021). Prevalence and Correlates of Frequent and Infrequent Bullying Victimization Among School Adolescents from Five Southeast Asian Countries. | No response |
| Pengpid, S., & Peltzer, K. (2017). Parental involvement, health behaviors and mental health among school-going adolescents in six Asian countries. | No response |
| Pengpid, S., & Peltzer, K. (2020). Prevalence and Associated Factors of Loneliness Among National Samples of In-School Adolescents in Four Caribbean Countries. Psychological Reports, 0033294120968502. | Outcome not of interest |
| Pengpid, S., & Peltzer, K. (2021). Prevalence and associated factors of psychological distress among a national sample of in-school adolescents in Liberia. Journal of Psychology in Africa, 31(2), 197-202. | Population not of interest |
| Peprah, P., Asare, B. Y. A., Nyadanu, S. D., Asare-Doku, W., Adu, C., Peprah, J., ... & Gyasi, R. M. (2023). Bullying victimization and suicidal behavior among adolescents in 28 countries and territories: a moderated mediation model. Journal of adolescent health, 73(1), 110-117. | Population not of interest |
| Pérez-Martínez, V., Sanz-Barbero, B., Ferrer-Cascales, R., Bowes, N., Ayala, A., Sánchez-SanSegundo, M., ... & Vives-Cases, C. (2020). The role of social support in machismo and acceptance of violence among adolescents in Europe. Lights4Violence baseline results. Journal of Adolescent Health. | Outcome not of interest |
| Perren, S., & Hornung, R. (2005). Bullying and delinquency in adolescence: Victims’ and perpetrators’ family and peer relations. Swiss Journal of Psychology, 64(1), 51-64. | No validated measure for PF |
| Perret, L. C., Orri, M., Boivin, M., Ouellet‐Morin, I., Denault, A. S., Côté, S. M., ... & Geoffroy, M. C. (2020). Cybervictimization in adolescence and its association with subsequent suicidal ideation/attempt beyond face‐to‐face victimization: a longitudinal population‐based study. Journal of child psychology and psychiatry, 61(8), 866-874. | Outcome not of interest |
| Picazo, M. F., Redondo, S. C., & Martinez-Pampliega, A. (2021). Interparental Conflict and Bullying: Assessment of the Mediating Role of Mentalization and Emotion Regulation/Conflicto Interparental y Acoso Escolar: Evaluación del Rol Mediador de la Mentalización y Regulación Emocional. Revista Iberoamericana de Diagnóstico y Evaluación-e Avaliação Psicológica, (61), 131-147. | Another language |
| Pinquart, M., & Pfeiffer, J. P. (2015). Bullying in students with and without hearing loss. Deafness & Education International, 17(2), 101-110. | No response |
| Pistella, J., Ioverno, S., & Russell, S. T. (2019). The role of peer victimization, sexual identity, and gender on unhealthy weight control behaviors in a representative sample of Texas youth. International journal of eating disorders, 52(5), 597-601. | Outcome not of interest |
| Pittet, I., Berchtold, A., Akré, C., Michaud, P. A., & Suris, J. C. (2010). Are adolescents with chronic conditions particularly at risk for bullying?. Archives of Disease in Childhood, 95(9), 711-716. | Population not of interest |
| Plexousakis, S. S., Kourkoutas, E., Giovazolias, T., Chatira, K., & Nikolopoulos, D. (2019). School bullying and post-traumatic stress disorder symptoms: the role of parental bonding. Frontiers in public health, 7, 75. | No response |
| Pouwelse, M., Bolman, C., Lodewijkx, H., & Spaa, M. (2011). Gender differences and social support: Mediators or moderators between peer victimization and depressive feelings?. Psychology in the Schools, 48(8), 800-814. | Outcome not of interest |
| Puhl, R. M., Lessard, L. M., Foster, G. D., & Cardel, M. I. (2023). Parent–child communication about weight: Priorities for parental education and support. Pediatric Obesity, e13027. | Outcome not of interest |
| Pulido, R., Banks, C., Ragan, K., Pang, D., Blake, J. J., & McKyer, E. L. (2019). The impact of school bullying on physical activity in overweight youth: exploring race and ethnic differences. Journal of school health, 89(4), 319-327. | No response |
| Quarshie, E. N. B. (2021). Self-harm among school-going adolescent survivors of sexual violence victimisation: a cross-sectional study. Frontiers in sociology, 6, 605865. | Population not of interest |
| Quinlan-Davidson, M., Kiss, L., Devakumar, D., Cortina-Borja, M., Eisner, M., & Tourinho Peres, M. F. (2021). The role of social support in reducing the impact of violence on adolescents’ mental health in São Paulo, Brazil. PLoS ONE, 16(10), e0258036. | Population not of interest |
| Rahman, M. M., Rahman, M. M., Khan, M. M. A., Hasan, M., & Choudhury, K. N. (2020). Bullying victimization and adverse health behaviors among school‐going adolescents in South Asia: Findings from the global school‐based student health survey. Depression and anxiety, 37(10), 995-1006. | No validated measure for PF |
| Rajendran, K., Kruszewski, E., & Halperin, J. M. (2016). Parenting style influences bullying: a longitudinal study comparing children with and without behavioral problems. Journal of child psychology and psychiatry, 57(2), 188-195. | No validated measure for PF |
| Rakic, L., Santric-Milicevic, M., Nikolic, D., Vasic, M., Babic, U., Todorovic, J., ... & Milenkovic, S. (2021). The relationship between individual and family characteristics and cyberbullying exposure in a nationally representative sample of school-aged children living in Serbia. International Journal of Environmental Research and Public Health, 18(14), 7443. | Couldn't provide correlation |
| Ramos, C. P., Torres-Toukoumidis, A., Pedreira, M. C. C., & Havránková, T. (2022). The Perception of Cyberbullying by Adolescents in Rural and Urban Spain. In Communication and Smart Technologies: Proceedings of ICOMTA 2021 (pp. 573-582). Springer Singapore. | No validated measure for PF |
| Ran, H., Cai, L., He, X., Jiang, L., Wang, T., Yang, R., ... & Xiao, Y. (2020). Resilience mediates the association between school bullying victimization and self-harm in Chinese adolescents. Journal of affective disorders, 277, 115-120. | No response |
| Ran, H., Yang, Q., Fang, D., Che, Y., Chen, L., Liang, X., ... & Xiao, Y. (2023). Social indicators with serious injury and school bullying victimization in vulnerable adolescents aged 12–15 years: Data from the Global School-Based Student Survey. Journal of affective disorders, 324, 469-476. | Outcome not of interest |
| Ranum, B. M., Wichstrøm, L., Pallesen, S., Falch-Madsen, J., & Steinsbekk, S. (2021). Persistent short sleep from childhood to adolescence: child, parent and peer predictors. Nature and science of sleep, 163-175. | No validated measure for PF |
| Rao, J., Wang, H., Pang, M., Yang, J., Zhang, J., Ye, Y., ... & Dong, X. (2019). Cyberbullying perpetration and victimisation among junior and senior high school students in Guangzhou, China. Injury prevention, 25(1), 13-19. | No validated measure for PF |
| Rasalingam, A., Clench-Aas, J., & Raanaas, R. K. (2017). Peer victimization and related mental health problems in early adolescence: The mediating role of parental and peer support. The Journal of Early Adolescence, 37(8), 1142-1162. | No validated measure for PF |
| Rehima, M. H., Al-Tkhaynehb, K. M., & Al-Shamsc, W. K. The Factors of Cyberbullying among the Basic Stage Students in the City of Al-Ain: A study on a Sample of Parents in The City of Al-Ain. | Outcome not of interest |
| Reid, J. A., & Sullivan, C. J. (2009). A latent class typology of juvenile victims and exploration of risk factors and outcomes of victimization. Criminal Justice and Behavior, 36(10), 1001-1024. | No validated measure for PF |
| Reisen, A., Viana, M. C., & dos Santos Neto, E. T. (2019). Adverse childhood experiences and bullying in late adolescence in a metropolitan region of Brazil. Child abuse & neglect, 92, 146-156. | Population not of interest |
| Reiter-Purtill, J., Gowey, M. A., Austin, H., Smith, K. C., Rofey, D. L., Jenkins, T. M., ... & TeenView Study Group and in cooperation with Teen-LABS Consortium. (2017). Peer victimization in adolescents with severe obesity: the roles of self-worth and social support in associations with psychosocial adjustment. Journal of pediatric psychology, 42(3), 272-282. | Outcome not of interest |
| Renner, L. M., Schwab-Reese, L. M., Coppola, E. C., & Boel-Studt, S. (2020). The contribution of interpersonal violence victimization types to psychological distress among youth. Child Abuse & Neglect, 106, 104493. | Outcome not of interest |
| Rhee, S., Lee, S. Y., & Jung, S. H. (2017). Ethnic differences in bullying victimization and psychological distress: A test of an ecological model. Journal of adolescence, 60, 155-160. | No validated measure for PF |
| Rican, P. (1995). Family values may be responsible for bullying. Studia psychologica, 37(1), 31. | Full text not available |
| Říčan, P., Klicperová, M., & Koucká, T. Á. (1993). Families of bullies and their victims: A children's view. Studia Psychologica. | Full text not available |
| Rigby, K. (1993). School children's perceptions of their families and parents as a function of peer relations. The Journal of Genetic Psychology, 154(4), 501-513. | Outcome not of interest |
| Rigby, K. (1994). Psychosocial functioning in families of Australian adolescent schoolchildren involved in bully/victim problems. Journal of family therapy, 16(2), 173-187. | Outcome not of interest |
| Rigby, K. E. N. (2000). Effects of peer victimization in schools and perceived social support on adolescent well-being. Journal of adolescence, 23(1), 57-68. | No validated measure for PF |
| Rigby, K., & Slee, P. (1999). Suicidal ideation among adolescent school children, involvement in bully—victim problems, and perceived social support. Suicide and Life‐Threatening Behavior, 29(2), 119-130. | No validated measure for PF |
| Riley, F., Wright, M., Bokszczanin, A., & Essau, C. A. (2020). Poly-victimization in Polish adolescents: Risk factors and the moderating role of coping. Journal of interpersonal violence, 35(5-6), 1515-1538. | Population not of interest |
| Ringdal, R., Bjørnsen, H. N., Espnes, G. A., Bradley Eilertsen, M. E., & Moksnes, U. K. (2020). Bullying, social support and adolescents’ mental health: Results from a follow-up study. Scandinavian journal of public health, 1403494820921666. | Population not of interest |
| Risner, D. (2014). Bullying victimisation and social support of adolescent male dance students: an analysis of findings. Research in Dance Education, 15(2), 179-201. | Qualitative study |
| Roberto, A. J., Eden, J., Deiss, D. M., Savage, M. W., & Ramos-Salazar, L. (2017). The short-term effects of a cyberbullying prevention intervention for parents of middle school students. International journal of environmental research and public health, 14(9), 1038. | Outcome not of interest |
| Rodriguez-Rivas, M. E., Varela, J. J., González, C., & Chuecas, M. J. (2022). The role of family support and conflict in cyberbullying and subjective well-being among Chilean adolescents during the Covid-19 period. Heliyon, 8(4). | No validated measure for PF |
| Romero-Abrio, A., León-Moreno, C., Musitu-Ferrer, D., & Villarreal-González, M. E. (2019). Family functioning, self-concept and Cybervictimization: an analysis based on gender. Social Sciences, 8(2), 69. | No response |
| Roques, M., Laimou, D., Camps, F. D., Mazoyer, A. V., & El Husseini, M. (2020). Using a mixed-methods approach to analyze traumatic experiences and factors of vulnerability among adolescent victims of bullying. Frontiers in psychiatry, 10, 890. | Qualitative study |
| Ruiz, A. P., Oteiza-Nascimento, A., Toldos, M. P., Serrano-Marugán, I., & Martín-Babarro, J. (2019). Bullying and depression: the moderating effect of social support, rejection and victimization profile. Anales De Psicología/Annals of Psychology, 35(1), 1-10. | Outcome not of interest |
| Russo, L. X. (2020). Association of victimization from bullying with body mass index in schoolchildren. Cadernos de Saúde Pública, 36(10). | Another language |
| Sabah, A., Aljaberi, M. A., Lin, C. Y., & Chen, H. P. (2022). The Associations between Sibling Victimization, Sibling Bullying, Parental Acceptance-Rejection, and School Bullying. INTERNATIONAL JOURNAL OF ENVIRONMENTAL RESEARCH AND PUBLIC HEALTH, 19(23). doi:10.3390/ijerph192316346 | Population not of interest |
| Sabanci, Y., & Çekiç, A. (2019). The Relationship between Irrational Beliefs, Resilience, Psychological Needs, Cyberbullying and Cyber Victimization. Universal Journal of Educational Research, 7(3), 700-706. | Outcome not of interest |
| Sabramani, V., Idris, I. B., Ismail, H., Nadarajaw, T., Zakaria, E., & Kamaluddin, M. R. (2021). Bullying and Its Associated Individual, Peer, Family and School Factors: Evidence from Malaysian National Secondary School Students. INTERNATIONAL JOURNAL OF ENVIRONMENTAL RESEARCH AND PUBLIC HEALTH, 18(13). doi:10.3390/ijerph18137208 | No validated measure for PF |
| Saeed Ali, T., Karmaliani, R., Mcfarlane, J., Khuwaja, H. M., Somani, Y., Chirwa, E. D., & Jewkes, R. (2017). Attitude towards gender roles and violence against women and girls (VAWG): baseline findings from an RCT of 1752 youths in Pakistan. Global health action, 10(1), 1342454. | Outcome not of interest |
| Saeed Ali, T., Karmaliani, R., Mcfarlane, J., Khuwaja, H. M., Somani, Y., Chirwa, E. D., & Jewkes, R. (2017). Attitude towards gender roles and violence against women and girls (VAWG): baseline findings from an RCT of 1752 youths in Pakistan. Global health action, 10(1), 1342454. | Outcome not of interest |
| Sahin, M., & Sarı, S. V. (2011). Observation of peer bullying in Turkish primary schools according to different variables. Cypriot Journal of Educational Sciences, 5(4), 258-270. | Full text not available |
| Sahli, J., Mellouli, M., El Ghardallou, M., Limam, M., Gallas, M., Ammar, A., ... & Zedini, C. (2018). Bullying among Tunisian middle school students: the prevalence, psychosocial associated factors and perceived involvement of parents, teachers and classmates. Journal of research in health sciences, 18(2), 414. | Outcome not of interest |
| Saiz, M. J. S., Chacón, R. M. F., Abejar, M. G., Parra, M. D. S., Rubio, M. E. L., & Jiménez, S. Y. (2019). Personal and social factors which protect against bullying victimization. Enfermería Global, 18(2), 13-24. | No response |
| Saleh, A., Hapsah, H., Krisnawati, W., & Erfina, E. (2021). Parenting style and bullying behavior in adolescents. ENFERMERIA CLINICA, 31, S640-S643. doi:10.1016/j.enfcli.2021.07.009 | Full text not available |
| Salimi, N., Karimi-Shahanjarini, A., Rezapur-Shahkolai, F., Hamzeh, B., Roshanaei, G., & Babamiri, M. (2019). The effect of an anti-bullying intervention on male students' bullying-victimization behaviors and social competence: a randomized controlled trial in deprived urban areas. Journal of research in health sciences, 19(4), e00461. | Outcome not of interest |
| Saltz, S. B., Rozon, M., Pogge, D. L., & Harvey, P. D. (2020). Cyberbullying and its relationship to current symptoms and history of early life trauma: a study of adolescents in an acute inpatient psychiatric unit. The Journal of clinical psychiatry, 81(1), 0-0. | Full text not available |
| Sam, J., Wisniewski, P., Xu, H., Rosson, M. B., & Carroll, J. M. (2017, July). How Are Social Capital and Parental Mediation Associated with Cyberbullying and Cybervictimization Among Youth in the United States?. In the International Conference on Human-Computer Interaction (pp. 638-644). Springer, Cham. | No response |
| Samara, M., Massarwi, A. A., El-Asam, A., Hammuda, S., Smith, P. K., & Morsi, H. (2021). The Mediating Role of Bullying and Victimisation on the Relationship Between Problematic Internet Use and Substance Abuse Among Adolescents in the UK: The Parent-Child Relationship as a Moderator. FRONTIERS IN PSYCHIATRY, 12. doi:10.3389/fpsyt.2021.493385 | No validated measure for PF |
| Sampasa-Kanyinga, H., Bakwa-Kanyinga, F., Hamilton, H. A., & Chaput, J.-P. (2022). Cyberbullying involvement, parental support, and cannabis use among adolescents. Child Abuse & Neglect, 133, 1-8. doi:10.1016/j.chiabu.2022.105830 | Population not of interest |
| Sampasa-Kanyinga, H., Lalande, K., & Colman, I. (2020). Cyberbullying victimisation and internalising and externalising problems among adolescents: the moderating role of parent–child relationship and child's sex. Epidemiology and psychiatric sciences, 29. | Population not of interest |
| Sandhu, D., & Kaur, S. (2016). Reducing cyber-bullying and problem behaviors among students through parental group therapy. Pakistan Journal of Psychological Research, 383-401. | Outcome not of interest |
| Sansen, L. M., Iffland, B., & Neuner, F. (2014). Peer victimization predicts psychological symptoms beyond the effects of child maltreatment. Psychiatry research, 220(3), 1051-1058. | Population not of interest |
| Sapouna, M., & Wolke, D. (2013). Resilience to bullying victimization: The role of individual, family and peer characteristics. Child abuse & neglect, 37(11), 997-1006. | Outcome not of interest |
| Sapouna, M., Wolke, D., Vannini, N., Watson, S., Woods, S., Schneider, W., ... & Aylett, R. (2012). Individual and social network predictors of the short‐term stability of bullying victimization in the United Kingdom and Germany. British Journal of Educational Psychology, 82(2), 225-240. | Outcome not of interest |
| Schilling, R. A., & Wang, W. J. (2023). Cyberbullying victimization and the COVID-19 pandemic: a routine activity perspective. Journal of School Violence, 22(4), 517-528. doi:10.1080/15388220.2023.2248869 | No validated measure for PF |
| Schwartz, D., Dodge, K. A., Pettit, G. S., & Bates, J. E. (1997). The early socialization of aggressive victims of bullying. Child development, 68(4), 665-675. | Outcome not of interest |
| Segovia-González, M. M., Ramírez-Hurtado, J. M., & Contreras, I. (2023). Analyzing the Risk of Being a Victim of School Bullying. The Relevance of Students' Self-Perceptions. CHILD INDICATORS RESEARCH, 16(5), 2141-2163. doi:10.1007/s12187-023-10045-x | No response |
| Seiler, S. J., & Navarro, J. N. (2014). Bullying on the pixel playground: Investigating risk factors of cyberbullying at the intersection of children’s online-offline social lives. Cyberpsychology: Journal of Psychosocial Research on Cyberspace, 8(4). | No validated measure for PF |
| Semiz, S., & Lindberg, E. N. (2022). Investigation of Peer Bullying in Early Years on The Basis of Emotional Intelligence and Family Variables. HACETTEPE UNIVERSITESI EGITIM FAKULTESI DERGISI-HACETTEPE UNIVERSITY JOURNAL OF EDUCATION, 37(2), 764-781. doi:10.16986/HUJE.2020063983 | Another language |
| Sentenac, M., Gavin, A., Arnaud, C., Molcho, M., Godeau, E., & Gabhainn, S. N. (2011). Victims of bullying among students with a disability or chronic illness and their peers: a cross-national study between Ireland and France. Journal of Adolescent Health, 48(5), 461-466. | No validated measure for PF |
| Seo, H. J., Jung, Y. E., Kim, M. D., & Bahk, W. M. (2017). Factors associated with bullying victimization among Korean adolescents. Neuropsychiatric disease and treatment, 13, 2429. | No validated measure for PF |
| Shah, S. A., & Majoka, M. I. (2017). Bullying in schools: The role of parents. Rawal Medical Journal, 42(4), 567-570. | Outcome not of interest |
| Shah, S. A., & Majoka, M. I. (2017). Bullying in schools: The role of parents. Rawal Medical Journal, 42(4), 567-570. | No validated measure for PF |
| Shaheen, A. M., Hamdan, K. M., Albqoor, M., Othman, A. K., Amre, H. M., & Hazeem, M. N. A. (2019). Perceived social support from family and friends and bullying victimization among adolescents. Children and Youth Services Review, 107, 104503. | Population not of interest |
| Shahrour, G., Dardas, L. A., Al-Khayat, A., & Al-Qasem, A. (2020). Prevalence, correlates, and experiences of school bullying among adolescents: A national study in Jordan. School psychology international, 41(5), 430-453. | Outcome not of interest |
| Shaikh, M. A., Abio, A., Celedonia, K. L., & Lowery Wilson, M. (2019). Physical fighting among school-attending adolescents in Pakistan: Associated factors and contextual influences. International journal of environmental research and public health, 16(24), 5039. | Outcome not of interest |
| Shams, H., Garmaroudi, G., Nedjat, S., & Yekaninejad, M. S. (2018). Effect of education based on socio-ecological theory on bullying in students: an educational study. Electronic physician, 10(7), 7046. | Outcome not of interest |
| Sharma, B., Nam, E. W., Kim, H. Y., & Kim, J. K. (2016). The influence of witnessing inter-parental violence and bullying victimization in involvement in fighting among adolescents: evidence from a school-based cross-sectional survey in Peru. Journal of lifestyle medicine, 6(1), 27. | Population not of interest |
| Shaw, R. J., Currie, D. B., Smith, G. S., Brown, J., Smith, D. J., & Inchley, J. C. (2019). Do social support and eating family meals together play a role in promoting resilience to bullying and cyberbullying in Scottish school children?. SSM-population health, 9, 100485. | Outcome not of interest |
| Shen, A. C. T., Feng, J. Y., Feng, J. Y., Wei, H. S., Hsieh, Y. P., Huang, S. C. Y., & Hwa, H. L. (2019). Who gets protection? A national study of multiple victimization and child protection among Taiwanese children. Journal of interpersonal violence, 34(17), 3737-3761. | Outcome not of interest |
| Shields, A., & Cicchetti, D. (2001). Parental maltreatment and emotion dysregulation as risk factors for bullying and victimization in middle childhood. Journal of clinical child psychology, 30(3), 349-363. | Outcome not of interest |
| Shields, A., & Cicchetti, D. (2001). Parental maltreatment and emotion dysregulation as risk factors for bullying and victimization in middle childhood. Journal of clinical child psychology, 30(3), 349-363. | Outcome not of interest |
| Shongwe, M. C., Dlamini, L. P., Simelane, M. S., Masuku, S. K., & Shabalala, F. S. (2021). Are there Gender Differences in the Prevalence and Correlates of Bullying Victimization Among in-School Youth in Eswatini?. School Mental Health, 1-13. | Full text not available |
| Sidiropoulou, K., Drydakis, N., Harvey, B., & Paraskevopoulou, A. (2019). Family support, school-age and workplace bullying for LGB people. International Journal of Manpower. | Population not of interest |
| Sitnik-Warchulska, K., Wajda, Z., Wojciechowski, B., & Izydorczyk, B. (2021). The Risk of Bullying and Probability of Help-Seeking Behaviors in School Children: A Bayesian Network Analysis. FRONTIERS IN PSYCHIATRY, 12. doi:10.3389/fpsyt.2021.640927 | Outcome not of interest |
| Smith, A. U., & Norris, A. E. (2020). Parent communication and bullying among Hispanic adolescent girls. The Journal of School Nursing, 36(3), 222-232. | No validated measure for PF |
| Smith, J., Twemlow, S. W., & Hoover, D. W. (1999). Bullies, victims and bystanders: A method of in-school intervention and possible parental contributions. Child psychiatry and human development, 30(1), 29-37. | Outcome not of interest |
| Smokowski, P. R., Evans, C. B., & Cotter, K. L. (2014). The Differential Impacts of Episodic, Chronic, and Cumulative Physical Bullying and Cyberbullying: The Effects of Victimization on the School Experiences, Social Support, and Mental Health of Rural Adolescents. Violence and victims, 29(6), 1029-1046. | No validated measure for PF |
| Sorrentino, A., Santamato, M., & Aquino, A. (2023). Individual, Familial, and School Risk Factors Affecting Teen Dating Violence in Early Adolescents: A Longitudinal Path Analysis Model. SOCIETIES, 13(9). doi:10.3390/soc13090213 | Outcome not of interest |
| Spears, B. A., Taddeo, C. M., Daly, A. L., Stretton, A., & Karklins, L. T. (2015). Cyberbullying, help-seeking and mental health in young Australians: implications for public health. International journal of public health, 60(2), 219-226. | Outcome not of interest |
| Speechley, M., & Stuart, J. (2022). The conditional effects of parental internet supervision on online victimization for early adolescent boys. Journal of Broadcasting & Electronic Media, 66(3), 420-439. doi:10.1080/08838151.2022.2099394 | Full text not available |
| Spriggs, A. L., Iannotti, R. J., Nansel, T. R., & Haynie, D. L. (2007). Adolescent bullying involvement and perceived family, peer and school relations: Commonalities and differences across race/ethnicity. Journal of Adolescent Health, 41(3), 283-293. | Outcome not of interest |
| Ssenyonga, J., Muwonge, C. M., & Hecker, T. (2019). Prevalence of family violence and mental health and their relation to peer victimization: A representative study of adolescent students in Southwestern Uganda. Child abuse & neglect, 98, 104194. | Outcome not of interest |
| Stange, J. P., Hamilton, J. L., Abramson, L. Y., & Alloy, L. B. (2014). A vulnerability-stress examination of response styles theory in adolescence: Stressors, sex differences, and symptom specificity. Journal of Clinical Child & Adolescent Psychology, 43(5), 813-827. | Outcome not of interest |
| Stavrinides, P., Tantaros, S., Georgiou, S., & Tricha, L. (2018). Longitudinal associations between parental rejection and bullying/victimization. Emotional and behavioural difficulties, 23(2), 203-212. | Outcome not of interest |
| Sterzing, P. R., Auslander, W. F., & Goldbach, J. T. (2014). An exploratory study of bullying involvement for sexual minority youth: Bully-only, victim-only, and bully-victim roles. Journal of the Society for Social Work and Research, 5(3), 321-337. | Population not of interest |
| Sterzing, P. R., Auslander, W. F., Ratliff, G. A., Gerke, D. R., Edmond, T., & Jonson-Reid, M. (2020). Exploring bullying perpetration and victimization among adolescent girls in the child welfare system: Bully-only, victim-only, bully-victim, and noninvolved roles. Journal of interpersonal violence, 35(5-6), 1311-1333. | Population not of interest |
| Sterzing, P. R., Ratliff, G. A., Gartner, R. E., McGeough, B. L., & Johnson, K. C. (2017). Social ecological correlates of polyvictimization among a national sample of transgender, genderqueer, and cisgender sexual minority adolescents. Child Abuse & Neglect, 67, 1-12. | Population not of interest |
| Stevens, V., De Bourdeaudhuij, I., & Van Oost, P. (2002). Relationship of the family environment to children's involvement in bully/victim problems at school. Journal of youth and Adolescence, 31(6), 419-428. | No response |
| Strøm, I. F., Thoresen, S., Wentzel-Larsen, T., Sagatun, Å., & Dyb, G. (2014). A prospective study of the potential moderating role of social support in preventing marginalization among individuals exposed to bullying and abuse in junior high school. Journal of youth and adolescence, 43(10), 1642-1657. | No validated measure for PF |
| Stubbs-Richardson, M., & May, D. C. (2020). Social Contagion in Bullying: an Examination of Strains and Types of Bullying Victimization in Peer Networks. American Journal of Criminal Justice, 1-22. | Outcome not of interest |
| Sullivan, T. N., Ross, K. M., O'Connor, K. E., Walsh, C. S., & Bishop, D. (2023). Relations Between Violence Exposure and Gun Carriage: Identifying Protective Factors Among African American Youth Living in Low-Income Urban Communities. Youth & Society. doi:10.1177/0044118X231172531 | No validated measure for PF |
| Sumter, S. R., & Baumgartner, S. E. (2017). Psychosomatic complaints in adolescence: Untangling the relationship between offline and online peer victimization, psychosomatic complaints and social support. European journal of developmental psychology, 14(4), 399-415. | Outcome not of interest |
| Sun, L., Liang, L., & Bian, Y. (2017). Parental psychological control and peer victimization among Chinese adolescents: The effect of peer pressure as a mediator. Journal of Child and Family Studies, 26(12), 3278-3287. | Outcome not of interest |
| Sušac, N., Ajduković, M., & Rimac, I. (2016). The Frequency of Peer Violence with Respect to Characteristics of Adolescents and Experienced Violence in the Family. Psychological Topics, 25(2), 197-221. | Another language |
| Taliaferro, L. A., & Muehlenkamp, J. J. (2017). Nonsuicidal self-injury and suicidality among sexual minority youth: risk factors and protective connectedness factors. Academic pediatrics, 17(7), 715-722. | Outcome not of interest |
| Tarablus, T., Heiman, T., & Olenik-Shemesh, D. (2015). Cyber bullying among teenagers in Israel: An examination of cyber bullying, traditional bullying, and socioemotional functioning. Journal of Aggression, Maltreatment & Trauma, 24(6), 707-720. | Couldn't provide correlation |
| Tarafa, H., Alemayehu, Y., Bete, T., & Tarecha, D. (2022). Bullying victimization and its associated factors among adolescents in Illu Abba Bor Zone, Southwest Ethiopia: a cross-sectional study. BMC psychology, 10(1), 260. | Population not of interest |
| Tennakoon, H., Saridakis, G., & Mohammed, A. M. (2018). Child online safety and parental intervention: a study of Sri Lankan internet users. Information Technology & People. | Outcome not of interest |
| Terranova, A. M. (2009, October). Factors that influence children’s responses to peer victimization. In Child & Youth Care Forum (Vol. 38, No. 5, pp. 253-271). Springer US. | Outcome not of interest |
| Terranova, A. M., Harris, J., Kavetski, M., & Oates, R. (2011, December). Responding to peer victimization: A sense of control matters. In Child & Youth Care Forum (Vol. 40, No. 6, pp. 419-434). Springer US. | Outcome not of interest |
| Terrell, A., Wickrama, T., Merten, M. J., & Becnel, J. (2022). Adolescents’ adverse family context and intimate partner violence: Mediating role of social media experience. Current Psychology: A Journal for Diverse Perspectives on Diverse Psychological Issues, No Pagination Specified-No Pagination Specified. doi:10.1007/s12144-022-03160-5 | Outcome not of interest |
| Thrasher, S. S., Malm, E. K., & Kim, C. (2022). The Associations between Father Involvement and Father-Daughter Relationship Quality on Girls' Experience of Social Bullying Victimization. CHILDREN-BASEL, 9(12). doi:10.3390/children9121976 | Outcome not of interest |
| Tillyer, M. S., Ray, J. V., & Hinton, M. E. (2018). Protecting high-risk youth in high-risk contexts: Neighborhoods, parenting, and victimization. Journal of youth and adolescence, 47(10), 2027-2040. | Outcome not of interest |
| Timmermanis, V., & Wiener, J. (2011). Social correlates of bullying in adolescents with attention-deficit/hyperactivity disorder. Canadian Journal of School Psychology, 26(4), 301-318. | No response |
| Tintori, A., Ciancimino, G., Bombelli, I., De Rocchi, D., & Cerbara, L. (2023). Children's Online Safety: Predictive Factors of Cyberbullying and Online Grooming Involvement. SOCIETIES, 13(2). doi:10.3390/soc13020047 | Population not of interest |
| Tintori, A., Ciancimino, G., Giovanelli, G., & Cerbara, L. (2021). Bullying and Cyberbullying among Italian Adolescents: The Influence of Psychosocial Factors on Violent Behaviours. INTERNATIONAL JOURNAL OF ENVIRONMENTAL RESEARCH AND PUBLIC HEALTH, 18(4). doi:10.3390/ijerph18041558 | No validated measure for PF |
| Tippett, N., & Wolke, D. (2015). Aggression between siblings: Associations with the home environment and peer bullying. Aggressive behavior, 41(1), 14-24. | No response |
| Ttofi, M. M., & Farrington, D. P. (2008). Reintegrative shaming theory, moral emotions and bullying. Aggressive Behavior: Official Journal of the International Society for Research on Aggression, 34(4), 352-368. | Outcome not of interest |
| Tu, K. M., Erath, S. A., Pettit, G. S., & Vandenberg, C. (2021). Parents’ responses to peer victimization: Associations with early adolescent coping and peer victimization. The Journal of Early Adolescence, 41(1), 167-196. | No validated measure for PF |
| Tucker, C. J., Finkelhor, D., & Turner, H. (2020). Family predictors of sibling versus peer victimization. Journal of Family Psychology, 34(2), 186. | Outcome not of interest |
| Tugran, A. Z., & Dost, M. T. (2022). The Relationship Between Parental Attitudes, Basic Psychological Needs, and Bullying Status in High School Students. EGITIM VE BILIM-EDUCATION AND SCIENCE, 47(209), 293-309. doi:10.15390/EB.2022.11034 | Full text not available |
| Türkileri, N., Soydas, D. K., & Uçanok, Z. (2021). Does the Associations among Perceived Parental Monitoring, Cyberbullying, and Life Satisfaction of Adolescents Differ in Boys and Girls? STUDIES IN PSYCHOLOGY-PSIKOLOJI CALISMALARI DERGISI, 41(3), 959-990. doi:10.26650/SP2020-849445 | Full text not available |
| Turner, H. A., Finkelhor, D., Shattuck, A., & Hamby, S. (2012). Recent victimization exposure and suicidal ideation in adolescents. Archives of pediatrics & adolescent medicine, 166(12), 1149-1154. | Outcome not of interest |
| Turner, H. A., Shattuck, A., Hamby, S., & Finkelhor, D. (2013). Community disorder, victimization exposure, and mental health in a national sample of youth. Journal of health and social behavior, 54(2), 258-275. | Outcome not of interest |
| Tyler, K. A., & Schmitz, R. M. (2018). Bullying at school and on the street: risk factors and outcomes among homeless youth. Journal of interpersonal violence, 0886260518794024. | Outcome not of interest |
| Uludasdemir, D., & Kucuk, S. (2019). Cyber bullying experiences of adolescents and parental awareness: Turkish example. Journal of pediatric nursing, 44, e84-e90. | No response |
| Unnever, J. D. (2005). Bullies, aggressive victims, and victims: Are they distinct groups?. Aggressive Behavior: Official Journal of the International Society for Research on Aggression, 31(2), 153-171. | No response |
| Vale, A., Pereira, F., Gonçalves, M., & Matos, M. (2018). Cyber-aggression in adolescence and internet parenting styles: A study with victims, perpetrators and victim-perpetrators. Children and Youth Services Review, 93, 88-99. | No response |
| Valido, A., Rivas-Koehl, M., Rivas-Koehl, D., Espelage, D. L., Lawrence, T. I., & Robinson, L. E. (2022). Latent Class Analysis of Victimization Patterns and Associated Protective Factors among LGBTQ Youth. INTERNATIONAL JOURNAL OF ENVIRONMENTAL RESEARCH AND PUBLIC HEALTH, 19(16). doi:10.3390/ijerph19169953 | No validated measure for PF |
| van Harmelen, A. L., Gibson, J. L., St Clair, M. C., Owens, M., Brodbeck, J., Dunn, V., ... & Goodyer, I. M. (2016). Friendships and family support reduce subsequent depressive symptoms in at-risk adolescents. PloS one, 11(5), e0153715. | Outcome not of interest |
| Van Niejenhuis, C., Huitsing, G., & Veenstra, R. (2020). Working with parents to counteract bullying: A randomized controlled trial of an intervention to improve parent‐school cooperation. Scandinavian journal of psychology, 61(1), 117-131. | Outcome not of interest |
| Vega López, M. G., González Pérez, G. J., Valle Barbosa, M. A., Flores Villavicencio, M. E., & Vega López, A. (2013). Bullying in the metropolitan area of Guadalajara, Mexico: prevalence and associated factors. Salud colectiva, 9(2), 183-194. | Outcome not of interest |
| Velki, T., & Kuterovac Jagodić, G. (2016). Can We Also Predict Electronic Peer Violence Based On the Predictors of Traditional Peer Violence?. Društvena istraživanja: časopis za opća društvena pitanja, 25(4), 523-545. | Another language |
| Vezne, R., Atman Uslu, N., & Yildiz Durak, H. (2022). Modeling of variables related to parents’ awareness in cyberbullying prevention. Current Psychology: A Journal for Diverse Perspectives on Diverse Psychological Issues, No Pagination Specified-No Pagination Specified. doi:10.1007/s12144-022-03837-x | Outcome not of interest |
| Vieira, M. A., Rønning, J. A., Mari, J. D. J., & Bordin, I. A. (2019). Does cyberbullying occur simultaneously with other types of violence exposure?. Brazilian Journal of Psychiatry, 41(3), 234-237. | Outcome not of interest |
| Vliek, L., Overbeek, G., & Orobio de Castro, B. (2019). Effects of Topper Training on psychosocial problems, self-esteem, and peer victimisation in Dutch children: A randomised trial. PloS one, 14(11), e0225504. | Outcome not of interest |
| Volikova, S. V., & Kalinkina, E. A. (2015). Parent-child relationships as a factor of school bullying. Konsul’tativnaya psikhologiya i psikhoterapiya [Counseling Psychology and Psychotherapy], 23(4), 138-161. | Another language |
| von Klitzing, K., Perren, S., Klein, A. M., Stadelmann, S., White, L. O., Groeben, M., ... & Hatzinger, M. (2012). The interaction of social risk factors and HPA axis dysregulation in predicting emotional symptoms of five-and six-year-old children. Journal of Psychiatric Research, 46(3), 290-297. | Outcome not of interest |
| Waasdorp, T. E., Bradshaw, C. P., & Duong, J. (2011). The link between parents' perceptions of the school and their responses to school bullying: Variation by child characteristics and the forms of victimization. Journal of Educational Psychology, 103(2), 324. | No validated measure for PF |
| Walters, G. D., & Espelage, D. L. (2018). Exploring the victimization‒early substance misuse relationship: In search of moderating and mediating effects. Child abuse & neglect, 81, 354-365. | No validated measure for PF |
| Wandera, S. O., Clarke, K., Knight, L., Allen, E., Walakira, E., Namy, S., ... & Devries, K. (2017). Violence against children perpetrated by peers: A cross-sectional school-based survey in Uganda. Child abuse & neglect, 68, 65-73. | Outcome not of interest |
| Wang, C. X., Liu, J. L., Atwal, K., & Do, K. A. (2021). Bullying Prevention for Asian American Families <i>Collaborations With School Districts and Community Organizations</i>. | Qualitative study |
| Wang, C., La Salle, T., Wu, C., Do, K. A., & Sullivan, K. E. (2018). School climate and parental involvement buffer the risk of peer victimization on suicidal thoughts and behaviors among Asian American middle school students. *Asian American Journal of Psychology*, *9*(4), 296–307. | Secondary report |
| Wang, G. F., Jiang, L., Wang, L. H., Hu, G. Y., Fang, Y., Yuan, S. S., ... & Su, P. Y. (2019). Examining childhood maltreatment and school bullying among adolescents: a cross-sectional study from Anhui Province in China. Journal of interpersonal violence, 34(5), 980-999. | No validated measure for PF |
| Wang, H., Tang, J. J., Dill, S. E., Xiao, J. S., Boswell, M., Cousineau, C., & Rozelle, S. (2022). Bullying Victims in Rural Primary Schools: Prevalence, Correlates, and Consequences. INTERNATIONAL JOURNAL OF ENVIRONMENTAL RESEARCH AND PUBLIC HEALTH, 19(2). doi:10.3390/ijerph19020765 | Outcome not of interest |
| Wang, H., Zhou, X., Lu, C., Wu, J., Deng, X., Hong, L., ... & He, Y. (2012). Adolescent bullying involvement and psychosocial aspects of family and school life: A cross-sectional study from Guangdong Province in China. PLoS One, 7(7), e38619. | Population not of interest |
| Wang, M. S., Hong, J. S., Wei, H. S., & Hwang, Y. T. (2019). Multiple level factors associated with bullying victimization in Taiwanese middle school students. Journal of school violence, 18(3), 375-387. | No validated measure for PF |
| Whelan, Y. M., Kretschmer, T., & Barker, E. D. (2014). MAOA, early experiences of harsh parenting, irritable opposition, and bullying–victimization: a moderated indirect-effects analysis. Merrill-Palmer Quarterly (1982-), 60(2), 217-237. | Outcome not of interest |
| Wienke Totura, C. M., MacKinnon-Lewis, C., Gesten, E. L., Gadd, R., Divine, K. P., Dunham, S., & Kamboukos, D. (2009). Bullying and victimization among boys and girls in middle school: The influence of perceived family and school contexts. The Journal of Early Adolescence, 29(4), 571-609. | Outcome not of interest |
| Williams, T., Connolly, J., Pepler, D., & Craig, W. (2005). Peer victimization, social support, and psychosocial adjustment of sexual minority adolescents. Journal of Youth and Adolescence, 34(5), 471-482. | Population not of interest |
| Williamson, R. E., Reed, D. E., Wickham, R. E., & Field, N. P. (2018). The mediational role of posttraumatic stress in the relationship between domestic violence exposure and peer victimisation: a Cambodian sample. Emotional and behavioural difficulties, 23(1), 28-38. | Population not of interest |
| Wilson, M. L., Celedonia, K. L., & Kamala, B. A. (2013). Patterns, characteristics, and correlates of adolescent bully-victims in urban Tanzania. Social Sciences, 2(4), 234-246. | No response |
| Winsper, C., Hall, J., Strauss, V. Y., & Wolke, D. (2017). Aetiological pathways to Borderline Personality Disorder symptoms in early adolescence: childhood dysregulated behaviour, maladaptive parenting and bully victimisation. Borderline personality disorder and emotion dysregulation, 4(1), 1-10. | No validated measure for PF |
| Wolfe, D. A., Crooks, C. C., Chiodo, D., & Jaffe, P. (2009). Child maltreatment, bullying, gender-based harassment, and adolescent dating violence: Making the connections. Psychology of Women Quarterly, 33(1), 21-24. | Qualitative study |
| Wood, C., & Orpinas, P. (2020). Victimization of children with disabilities: coping strategies and protective factors. Disability & Society, 1-20. | Outcome not of interest |
| Wood, C., & Orpinas, P. (2021). Victimization of children with disabilities: Coping strategies and protective factors. Disability & Society, 36(9), 1469-1488. doi:10.1080/09687599.2020.1802578 | Population not of interest |
| Wormington, S. V., Anderson, K. G., Tomlinson, K. L., & Brown, S. A. (2013). Alcohol and other drug use in middle school: The interplay of gender, peer victimization, and supportive social relationships. The Journal of early adolescence, 33(5), 610-634. | Outcome not of interest |
| Worsley, J. D., McIntyre, J. C., & Corcoran, R. (2019). Cyberbullying victimisation and mental distress: Testing the moderating role of attachment security, social support, and coping styles. Emotional and behavioural difficulties, 24(1), 20-35. | Population not of interest |
| Wright, M. (2018). Cyberbullying victimization through social networking sites and adjustment difficulties: The role of parental mediation. Journal of the Association for Information Systems, 19(2), 1. | No validated measure for PF |
| Wright, M. F. (2015). Cyber victimization and adjustment difficulties: The mediation of Chinese and American adolescents’ digital technology usage. Cyberpsychology: Journal of Psychosocial Research on Cyberspace, 9(1). | No validated measure for PF |
| Wright, M. F. (2015). Cyber victimization and perceived stress: Linkages to late adolescents’ cyber aggression and psychological functioning. Youth & Society, 47(6), 789-810. | Outcome not of interest |
| Wright, M. F. (2016). The buffering effect of parental mediation in the relationship between adolescents' cyberbullying victimisation and adjustment difficulties. Child abuse review, 25(5), 345-358. | No validated measure for PF |
| Wright, M. F. (2016). The roles of bullying involvement and parental warmth in non-suicidal self-harm and suicidal ideation among adolescents from residential program. Journal of criminal psychology. | Outcome not of interest |
| Wright, M. F. (2017). Parental mediation, cyberbullying, and cybertrolling: The role of gender. Computers in Human Behavior, 71, 189-195. | No validated measure for PF |
| Wright, M. F. (2017). Victimized children’s adjustment difficulties: The role of parenting styles and parents’ childhood peer victimization status. Journal of Aggression, Maltreatment & Trauma, 26(5), 493-506. | Outcome not of interest |
| Wright, M. F. (2018). Cyberstalking victimization, depression, and academic performance: The role of perceived social support from parents. Cyberpsychology, Behavior, and Social Networking, 21(2), 110-116. | Population not of interest |
| Wright, M. F., & Wachs, S. (2018). Does parental mediation moderate the longitudinal association among bystanders and perpetrators and victims of cyberbullying?. Social Sciences, 7(11), 231. | No validated measure for PF |
| Wright, M. F., & Wachs, S. (2019). Does parental mediation of technology use moderate the associations between cyber aggression involvement and substance use? A three-year longitudinal study. International journal of environmental research and public health, 16(13), 2425. | No validated measure for PF |
| Wright, M. F., Wachs, S., & Gámez-Guadix, M. (2021). Youths’ coping with cyberhate: Roles of parental mediation and family support. Comunicar, 29(67). | Outcome not of interest |
| Wu, C. Y., Astor, R. A., & Benbenishty, R. (2023). A Large-Scale Longitudinal Study of School Factors as Mediators from Depression and Parental Monitoring to Peer Victimization in Mainland Chinese Middle Schools. Journal of School Violence. doi:10.1080/15388220.2023.2261365 | Outcome not of interest |
| Wu, N. N., Mo, J. H., Wen, A. L., Ou, H. E., Gu, W. X., Qiu, Y. Q., . . . Lan, X. Y. (2023). Longitudinal Relationship between Bullying Victimization and Non-Suicidal Self-Injury among Chinese Adolescents: The Buffering Roles of Gratitude and Parental Autonomy Support. INTERNATIONAL JOURNAL OF ENVIRONMENTAL RESEARCH AND PUBLIC HEALTH, 20(2). doi:10.3390/ijerph20021440 | Outcome not of interest |
| Xiao, Y., Jiang, L., Yang, R., Ran, H., Wang, T., He, X., ... & Lu, J. (2021). Childhood maltreatment with school bullying behaviors in Chinese adolescents: A cross-sectional study. Journal of affective disorders, 281, 941-948. | Outcome not of interest |
| Xiao, Y., Ran, H., Fang, D., Che, Y., Donald, A. R., Wang, S., . . . Lu, J. (2022). School bullying associated suicidal risk in children and adolescents from Yunnan, China: The mediation of social support [Elsevier Science doi:10.1016/j.jad.2022.01.032]. Retrieved | No response |
| Xie, Q. W., Zhao, G. L., Lu, J. J., Chen, R. J., Xu, J. Y., Wang, M. W., . . . Zhou, X. D. (2023). Mental Health Problems amongst Left-behind Adolescents in China: Serial Mediation Roles of Parent-Adolescent Communication and School Bullying Victimisation. BRITISH JOURNAL OF SOCIAL WORK, 53(2), 994-1018. doi:10.1093/bjsw/bcac168 | No response |
| Xin, M. C., Chen, P., Liang, Q., Yu, C. F., Zhen, S. J., & Zhang, W. (2021). Cybervictimization and Adolescent Internet Addiction: A Moderated Mediation Model. INTERNATIONAL JOURNAL OF ENVIRONMENTAL RESEARCH AND PUBLIC HEALTH, 18(5). doi:10.3390/ijerph18052427 | No validated measure for PF |
| Xing, J., Fong, T. C. T., & Ho, R. T. H. (2021). Validation of the Actually Received Support scale for Chinese adolescents experiencing school bullying [Springer doi:10.1007/s10826-021-01976-6]. Retrieved | Outcome not of interest |
| Xiong, Q., Shi, S., Chen, J., Hu, Y., Zheng, X., Li, C., & Yu, Q. (2020). Examining the link between academic achievement and adolescent bullying: a moderated moderating model. Psychology research and behavior management, 13, 919. | Outcome not of interest |
| Xu, S., Ren, J., Li, F., Wang, L., & Wang, S. (2020). School bullying among vocational school students in China: prevalence and associations with personal, relational, and school factors. Journal of interpersonal violence, 0886260520907360. | Outcome not of interest |
| YABAN, E., Sayil, M., & KINDAP TEPE, Y. E. L. İ. Z. (2013). The relations between perceived parental support and peer bullying/victimization in male adolescents: The role of friendship quality. | Another language |
| Yang, C., Sharkey, J. D., Chen, C., & Jimerson, S. (2019). Teacher–Home Communication and Bullying Victimization: Do Parents’ Perceptions of Fairness of Rules Matter?. School psychology review, 48(3), 251-266. | Outcome not of interest |
| Yang, Y. D., Zheng, C. J., Xie, M., Yuan, S. Q., Zeng, Y., Zhou, M. L., . . . Baker, J. S. (2021). Bullying Victimization and Life Satisfaction Among Rural Left-Behind Children in China: A Cross-Sectional Study. FRONTIERS IN PEDIATRICS, 9. doi:10.3389/fped.2021.671543 | No response |
| Yang, Z. M., Tu, Y., Qin, Z. H., Liu, X. Q., & Lu, D. L. (2022). School bullying among migrant children in China: A cross-sectional study. Frontiers in Psychology, 13. doi:10.3389/fpsyg.2022.1027506 | No validated measure for PF |
| Ybarra, M. L., Espelage, D. L., & Mitchell, K. J. (2007). The co-occurrence of Internet harassment and unwanted sexual solicitation victimization and perpetration: Associations with psychosocial indicators. Journal of adolescent health, 41(6), S31-S41. | No validated measure for PF |
| Yen, C. F., Lai, C. Y., Ko, C. H., Liu, T. L., Tang, T. C., Wu, Y. Y., & Yang, P. (2014). The associations between suicidal ideation and attempt and anxiety symptoms and the demographic, psychological, and social moderators in Taiwanese adolescents. Archives of suicide research, 18(1), 104-116. | Outcome not of interest |
| Yen, C. F., Liu, T. L., Yang, P., & Hu, H. F. (2015). Risk and protective factors of suicidal ideation and attempt among adolescents with different types of school bullying involvement. Archives of Suicide Research, 19(4), 435-452. | Outcome not of interest |
| Yiğit, M. F., Keskin, S., & Yurdugül, H. (2018). Investigating the relationship between cyberbullying and perceived family support in middle-school students in relation to gender, frequency of internet use, and grade. Addicta: The Turkish Journal on Addictions, 5(2), 1-36. | Outcome not of interest |
| Yıldız İnanıcı, S. Different Types of Bully Experiences and Their Relationship with Attachment to Father and Moral Maturity. Child and Adolescent Social Work Journal, 1-10. | No response |
| YILDIZ İNANICI, S. İ. N. E. M. (2020). Different Types of Bully Experiences and Their Relationship with Attachment to Father and Moral Maturity. | No response |
| Yoo, C. (2021). What are the characteristics of cyberbullying victims and perpetrators among South Korean students and how do their experiences change?. Child Abuse & Neglect, 113, 104923. | Full text not available |
| Yoo, C. (2021). What are the characteristics of cyberbullying victims and perpetrators among South Korean students and how do their experiences change? Child Abuse & Neglect, 113. doi:10.1016/j.chiabu.2020.104923 | Outcome not of interest |
| Yoon, D., Shipe, S. L., Park, J., & Yoon, M. (2021). Bullying patterns and their associations with child maltreatment and adolescent psychosocial problems. Children and Youth Services Review, 129. doi:10.1016/j.childyouth.2021.106178 | Outcome not of interest |
| Yoon, D., Yoon, S., Park, J., & Yoon, M. (2018). A pernicious cycle: Finding the pathways from child maltreatment to adolescent peer victimization. Child abuse & neglect, 81, 139-148. | Outcome not of interest |
| Yosep, I., Pramukti, I., Agustina, H. R., Kurniawan, K., Agustina, H. S., & Hikmat, R. (2023). Triple-P e-Parenting to Improve Awareness of Psychiatric Nurses on Preventing Cyberbullying in Adolescents. HEALTHCARE, 11(1). doi:10.3390/healthcare11010019 | Outcome not of interest |
| You, S., Kim, E., & Kim, M. (2014). An ecological approach to bullying in Korean adolescents. Journal of Pacific Rim Psychology, 8(1), 1-10. | No validated measure for PF |
| Yourell, J. L., & Doty, J. L. (2022). Associations Between Weight-Based Bullying, Developmental Internal Assets, and Perceived Social Support Among Youth. JOURNAL OF SCHOOL HEALTH, 92(1), 42-51. doi:10.1111/josh.13101 | No validated measure for PF |
| Yuan, M.-Y., Li, Y.-H., Chang, J.-J., Zhang, T.-T., Wang, G.-F., & Su, P.-Y. (2022). Exploring the correlates of homicidal ideation in Chinese early adolescents: A network analysis. Journal of Affective Disorders, 314, 241-248. doi:10.1016/j.jad.2022.07.032 | No validated measure for PF |
| Yucel, D., & Yuan, A. S. V. (2016). Parents, siblings, or friends? Exploring life satisfaction among early adolescents. Applied Research in Quality of Life, 11(4), 1399-1423. | No validated measure for PF |
| Yudes, C., Rey, L., & Extremera, N. (2020). Predictive factors of cyberbullying perpetration amongst spanish adolescents. International journal of environmental research and public health, 17(11), 3967. | No validated measure for PF |
| Yüksel, A. S., Palmer, S. B., & Rutland, A. (2021). Developmental Differences in Bystander Behavior Toward Intergroup and Intragroup Exclusion. DEVELOPMENTAL PSYCHOLOGY, 57(8), 1342-1349. doi:10.1037/dev0001202 | Outcome not of interest |
| Yun, I., & Kim, S. G. (2016). Bullying among South Korean adolescents: Prevalence and association with psychological adjustment. Violence and victims, 31(1), 167-184. | No validated measure for PF |
| Yun, I., & Kim, S. G. (2016). Bullying among South Korean adolescents: Prevalence and association with psychological adjustment. Violence and victims, 31(1), 167-184. | No validated measure for PF |
| Zequinão, M. A., Oliveira, W. A. D., Medeiros, P. D., Cidade, P., Pereira, B., & Cardoso, F. L. (2020). Physical punishment at home and grade retention related to bullying. Journal of Human Growth and Development, 30(3), 434-442. | No validated measure for PF |
| Zervos, A. P., Hensel, D. J., James, R., Hunt, A., & Ott, M. A. (2022). The role of trauma and positive youth development in polysubstance use among rural middle school students: a latent class analysis. BMC Public Health, 22(1). doi:10.1186/s12889-022-14795-1 | Outcome not of interest |
| Zhai, B., Li, D., Jia, J., Liu, Y., Sun, W., & Wang, Y. (2019). Peer victimization and problematic internet use in adolescents: The mediating role of deviant peer affiliation and the moderating role of family functioning. Addictive behaviors, 96, 43-49. | Outcome not of interest |
| Zhang, H., Zhou, H., & Cao, R. (2019). Bullying victimization among left-behind children in rural China: Prevalence and associated risk factors. Journal of interpersonal violence, 0886260519843287. | Outcome not of interest |
| Zhang, H., Zhou, H., & Cao, R. (2021). Bullying victimization among left-behind children in rural China: Prevalence and associated risk factors. Journal of Interpersonal Violence, 36(15-16), NP8414-NP8430. doi:10.1177/0886260519843287 | No response |
| Zhang, S., Hong, J. S., Hao, Y., Lee, N. Y., & Piquero, A. R. (2020). A Latent Transition Analysis of Youth Bullying Victimization Patterns over Time and Their Relations to Delinquency. Journal of Interpersonal Violence, 0886260520958635. | Outcome not of interest |
| Zhang, X., Han, Z., & Ba, Z. (2020). Cyberbullying involvement and psychological distress among Chinese adolescents: the moderating effects of family cohesion and school cohesion. International journal of environmental research and public health, 17(23), 8938. | No validated measure for PF |
| Zhao, D. X., Wang, D., He, Z., Yuan, S. L., Zhu, D., & Liu, H. Y. (2022). Victim profiles and the protective role of school anti-bullying norms: a study of Chinese adolescents. CURRENT PSYCHOLOGY. doi:10.1007/s12144-022-04204-6 | No validated measure for PF |
| Zhao, M., Xiao, D., Wang, W., Wu, R., Zhang, W., Guo, L., & Lu, C. (2020). Association among maltreatment, bullying and mental health, risk behavior and sexual attraction in Chinese students. Academic pediatrics. | Outcome not of interest |
| Zhao, Q., Li, W., & Rukavina, P. B. (2017). Types and nature of parental support for overweight students to cope with weight‐related teasing. Journal of School Health, 87(10), 776-783. | Population not of interest |
| Zhao, Y. P., Lee, Y. T., Tang, Y. Q., & York, M. (2021). The Characteristics of Targets of Bullying Among Chinese Youth Attending Key Versus Non-Key Schools: A Mixed-Methods Analysis. Journal of Interpersonal Violence, 36(15-16), 6911-6930. doi:10.1177/0886260519845729 | No validated measure for PF |
| Zhao, Y., Hong, J. S., Zhao, Y., & Yang, D. (2021). Parent–Child, Teacher–Student, and Classmate Relationships and Bullying Victimization Among Adolescents in China: Implications for School Mental Health. School Mental Health, 1-11. | Population not of interest |
| Zhao, Y., Hong, J. S., Zhao, Y., & Yang, D. (2021). Parent–child, teacher–student, and classmate relationships and bullying victimization among adolescents in china: Implications for school mental health. School Mental Health: A Multidisciplinary Research and Practice Journal, No Pagination Specified-No Pagination Specified. doi:10.1007/s12310-021-09425-x | Population not of interest |
| Zhao, Y., Zhao, Y., Lee, Y. T., & Chen, L. (2020). Cumulative interpersonal relationship risk and resilience models for bullying victimization and depression in adolescents. Personality and individual differences, 155, 109706. | Population not of interest |
| Zheng, Y., Cai, D., Zhao, J. L., Yang, C., Xia, T., & Xu, Z. (2021, April). Bidirectional Relationship Between Emotional Intelligence and Perceptions of Resilience in Young Adolescents: A Twenty-Month Longitudinal Study. In Child & Youth Care Forum (Vol. 50, No. 2, pp. 363-377). Springer US. | Outcome not of interest |
| Zhou, P. Y., Dong, J. Q., Liu, J., Wen, H. B., & Wang, Z. (2023). The relationship between parent-child relationship and peer victimization: a multiple mediation model through peer relationship and depression. FRONTIERS IN BEHAVIORAL NEUROSCIENCE, 17. doi:10.3389/fnbeh.2023.1170891 | Secondary report |
| Zhou, Z., Tang, H., Tian, Y., Wei, H., Zhang, F., & Morrison, C. M. (2013). Cyberbullying and its risk factors among Chinese high school students. School psychology international, 34(6), 630-647. | No validated measure for PF |
| Zhu, Y., Chan, K. L., & Chen, J. (2018). Bullying victimization among Chinese middle school students: The role of family violence. Journal of interpersonal violence, 33(12), 1958-1977. | Outcome not of interest |
| Zhu, Y., Li, W., O’Brien, J. E., & Liu, T. (2019). Parent–child attachment moderates the associations between cyberbullying victimization and adolescents’ health/mental health problems: An exploration of cyberbullying victimization among Chinese adolescents. Journal of interpersonal violence, 0886260519854559. | Couldn't provide correlation |
| Zottis, G. A., Salum, G. A., Isolan, L. R., Manfro, G. G., & Heldt, E. (2014). Associations between child disciplinary practices and bullying behavior in adolescents. Jornal de Pediatria (Versão em Português), 90(4), 408-414. | Outcome not of interest |
| Zsila, Á., Orosz, G., Király, O., Urbán, R., Ujhelyi, A., Jármi, É., ... & Demetrovics, Z. (2018). Psychoactive substance use and problematic internet use as predictors of bullying and cyberbullying victimization. International journal of mental health and addiction, 16(2), 466-479. | Population not of interest |
| Zurcher, J. D., Holmgren, H. G., Coyne, S. M., Barlett, C. P., & Yang, C. (2018). Parenting and cyberbullying across adolescence. Cyberpsychology, Behavior, and Social Networking, 21(5), 294-303. | Outcome not of interest |
| Γεωργίου, Σ. Ν., & Φαίδωνος, Φ. (2011). Bullying and victimization at school: personal and family parameters. Psychology: the Journal of the Hellenic Psychological Society, 18(4), 484-502. | Another language |
